# Degradation Dynamics and Residue Analysis of Four Propiconazole Stereoisomers in “Fengtang” Plum during Storage by LC-MS/MS

**DOI:** 10.3390/foods12112200

**Published:** 2023-05-30

**Authors:** Pengyu Deng, Lianhong Mou, Guipeng Ou, Xin Luo, Deyu Hu, Yuping Zhang

**Affiliations:** National Key Laboratory of Green Pesticide, Key Laboratory of Green Pesticide and Agricultural Bioengineering, Ministry of Education, Center for R&D of Fine Chemicals, Guizhou University, Guiyang 550025, China; dengpengyu01@163.com (P.D.); lhmou321@163.com (L.M.); o15186147316@163.com (G.O.); 17601460021@163.com (X.L.); gzu_dyhu@126.com (D.H.)

**Keywords:** propiconazole, stereoisomers, stereoselective degradation, residues, LC−MS/MS, “Fengtang” plum

## Abstract

Herein, an accurate and sensitive method was developed for detecting four stereoisomers of propiconazole in “Fengtang” plum by LC-MS/MS. The mean recovery of four propiconazole stereoisomers ranged from 79.42 to 104.10% at three adding levels with reasonable RSD of 1.54–11.68%, and the LOD and LOQ of the four stereoisomers was 0.0005 mg/kg and 0.004 mg/kg, respectively. In addition, the residue and selective degradation of propiconazole stereoisomers in plums were investigated by storage at 20 °C and 4 °C. The half-lives of propiconazole stereoisomeric during storage were 9.49–15.40 d at 20 °C, and 21.00–28.88 d at 4 °C. The degradation of (2R,4R)-propiconazole and (2R,4S)-propiconazole in stored plums was slightly slower than that of the corresponding enantiomers (2S,4S)-propiconazole and (2S,4R)-propiconazole. The total residues of propiconazole were 0.026–0.487 mg/kg in the plum storage period, and the water washing could remove 49.35% to 54.65% of the propiconazole residue in plum. The hardness of plums treated with propiconazole was generally higher than that of control in the middle and late stages of storage. The effects of propiconazole on the total soluble solid content of plums were different at 20 °C and 4 °C. This study provides a scientific reference for the food safety evaluation of the “Fengtang” plum after the application of propiconazole during the storage period.

## 1. Introduction

“Fengtang” plum is a kind of plum cultivated in Guizhou, China, which enjoys the title of geographical landmark agricultural product [1]. It is also one of the fruit crops with the largest planting area in Guizhou, China, the “Fengtang” plum planting area as of 2019 was approximately 30,000 hm^2^, and the planting area is constantly expanding [2]. “Fengtang” plum is favored by many consumers because it is rich in vitamins, sugars, phenolics, flavonoids, and other nutrients needed by humans, the total sugar content of mature “Fengtang” plum was as high as 82.65 mg·g^−1^ FW [3,4]. “Fengtang” plum is a respiratory variable fruit, and the harvest time is concentrated in the hot season of June to July [5]. Due to the concentrated ripening period, good storage after harvest is very important to prolong the shelf-life of “Fengtang” plums. However, the postharvest “Fengtang” plums are often susceptible to pathogens such as Monilinia fructicola, Saccharomycetes, Aspergillus niger, Aspergillus oryzae, and other microorganisms, resulting in the rot or softening of plum fruit, the loss of commodity quality [6,7,8,9], and ultimately causing huge economic losses. Therefore, it is particularly important to take effective measures to prevent or reduce the decay and deterioration of the “Fengtang” plum due to microorganism invasion during storage, and one of the current effective methods is to spray appropriate concentrations of fungicides on the surface of “Fengtang” plum during storage.

Propiconazole is a triazole fungicide, which mainly affects the formation of the fungal cell wall by hindering the biosynthesis of fungal ergosterol [10,11]. Propiconazole is widely used to control plum brown rot, apple anthracnose, dragon-fruit brown rot, citrus acid rot, and grape powdery mildew because of its good control effect on fungal diseases [12]. In addition, propiconazole has a good controlling effect on the postharvest diseases of apple, pitaya, and citrus, and can significantly reduce the occurrence of fruit decay during storage [13,14,15,16]. However, research on propiconazole residues during storage is lacking. Since propiconazole is used as a postharvest antibacterial in some fruits, one has to be concerned about whether propiconazole residues are harmful to human health and whether it will degrade the quality of the fruit. Propiconazole has an acute oral toxicity LD_50_ of 1517 mg/kg bw in rats, it is a pesticide with low toxicity, is not carcinogenic, and is safe for mammals [17]. However, propiconazole disrupts the endocrine system, potentially exerting a cancer-inducing effect [18]. Propiconazole has been used in a variety of crops such as bananas, apples, and wolf-berries. Propiconazole preparations with an effective concentration of 25% are registered in apple trees and have a recommended dose of dilute with 1500 times the volume and a safe interval of 28 days [12]. Although propiconazole is a low-toxic pesticide, in order to ensure food safety, the maximum residue level (MRL) of propiconazole in a variety of plant foods has been established by different countries and organizations. For example, the MRL in plum is 0.6 mg/kg in China, and 0.01 mg/kg in the European Union [19,20]. At present, a number of methods have been established for the determination of the propiconazole residues in apples, bananas, strawberries, pears, celery, onion, wheat, soil, panax-notoginseng, tea, and other substrates. Xu and Li et al. established a gas chromatography-tandem mass spectrometry (GC-MS/MS) method for the analysis of propiconazole in bananas and apples [21,22]. Bai and Zhang et al. studied the degradation behavior and residual amount of propiconazole in celery, onion, wheat, and soil using high-performance liquid chromatography-tandem mass spectrometry (HPLC-MS/MS) [23,24]. Although propiconazole residues in a variety of substrates have been analyzed, these studies mainly focused on the residual and degradation of propiconazole at the achiral level, research on the residue analysis of propiconazole at the chiral level in plums is lacking.

**Figure 1:**
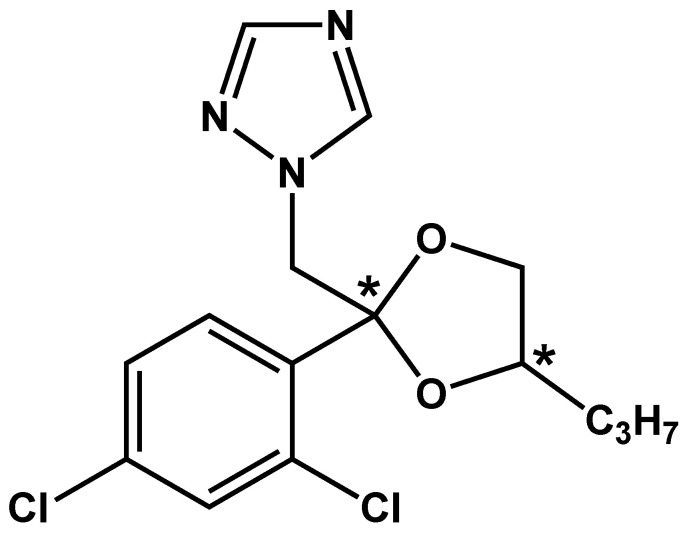
Structural formula of propiconazole (* indicates the chiral center).

Propiconazole is a chiral pesticide that contains two chiral centers (Figure 1) and corresponds to four optical isomers [25]. Different enantiomers of chiral pesticides may have different biological activities and toxicity, metabolism, degradation, and migration. The activity and environmental behavior of chiral stereoisomers are the hotspots in the current study. Tang et al. reported the stereoselective biological activity of propiconazole enantiomers against banana leaf spot pathogens (C. lunata and C. musae), the antibacterial activity of (2R,4S)-propiconazole was significantly higher than that of other stereoisomers [26]. Garrison et al. used capillary electrophoresis (CE) to explore the degradation of propiconazole and its four stereoisomers in the soil-cement phase [27]. Pan and Cheng et al. investigated the stereoselective digestion of propiconazole in paddy soil in wheat straw and grapes using supercritical fluid chromatography-tandem mass spectrometry (SFC-MS/MS) and found stereoselective degradation of the propiconazole stereoisomeric in grapes, oxygen-rich soil, and wheat straw [28,29]. Using ultra-performance liquid chromatography-tandem mass spectrometry (UPLC-MS/MS), Jiang et al. developed a method for the determination of propiconazole enantiomers in tea [30]. Establishing a sensitive and accurate method for the determination of the residues of propiconazole stereoisomers in environmental samples is crucial for reasonable evaluation of the degradation behavior of propiconazole stereoisomers in plums. At present, a chiral detection method for the determination of the four propiconazole stereoisomers in plums has not been published.

Removing pesticide residues on the surface of fruit is one of the most effective ways to reduce pesticide intake. To date, the commonly used methods for removing pesticides on the fruit’s surface include tap water cleaning, ultrasonic cleaning, fruit and vegetable cleaning agent cleaning, and peeling [31,32]. The cleaning effect of these methods is not related only to the nature of pesticides but also to the surface structures of fruit and vegetables and cleaning time [32,33]. Cleaning is the first step in the treatment of primary produce in most households. Considering the characteristics of fruit, this experiment explored the effect of tap water cleaning treatment on propiconazole residues in “Fengtang” plum.

The purpose of this study is to establish an accurate and sensitive method for the analysis of four stereoisomers of propiconazole in “Fengtang” plum. In addition, the degradation rates and residual levels of four stereoisomers of propiconazole in plums at different storage temperatures (4 °C and 20 °C) were measured by different doses of propiconazole (0.004 and 0.020 a.i. g/L). Moreover, the efficiency of removing propiconazole residues in “Fengtang” plums by water cleaning was evaluated. Finally, the firmness and total soluble solid content of “Fengtang” plums after propiconazole application was determined. To the best of our knowledge, this study reports for the first time the residual levels and dissipation rates of four propiconazole stereoisomers in stored plums. We may provide a scientific reference for food safety evaluation of the “Fengtang” plum after the application of propiconazole during the storage period.

## 2. Materials and Methods

### 2.1. Materials

The “Fengtang” plum at about 80% maturity was picked from a planting site in Anshun, Guizhou province, China, in June 2022. The analytical-grade methanol, acetonitrile, anhydrous magnesium sulfate (MgSO_4_), sodium chloride (NaCl), and other used chemistries were of analytical grade and were purchased from Platinum Strontium Titanium Chemical Products Co., Ltd. (Zunyi, China). Anpon Electrochemical Co. Ltd. (Huagonglu Huaiyin City, China) provided the standards for propiconazole (99.9% pure). Propiconazole EC (250 g/L) was purchased from Syngenta Jiangmen Crop Protection Co., Ltd. (Jiangmen, China). Watsons Food and Beverage Co. LTD (Guangzhou, China) provided the ultrapure water. Thermo Scientific (Waltham, MA, USA) provided the chromatography-grade ethyl alcohol. The 0.22 μm organic filter membrane was purchased from Sharp Technology (Shanghai, China). The vortex mixer was purchased from Qilin Medical Instrument Factory (QL-901, Haimen, China). The Haili Ultrasonic Electrical Appliance Co., Ltd. (Zhongshan, China) provided the ultrasonic instrument (KQ-100B). The centrifuge was bought from Anting Scientific Instrument Co., Ltd. (TG-10B, Shanghai, China) and the Yarong Biochemical Instrument Co., Ltd. (Shanghai, China) provided the rotary evaporator (RE-2000A).

### 2.2. Laboratory Processing

The plums were transported to the laboratory of Guizhou University. The fruits with basically the same color and size, no mechanical damage, and no pests and diseases were selected, after which they were placed overnight at room temperature to remove the field heat and set aside for use. With reference to the dosage of propiconazole spraying in the field, propiconazole solutions with effective concentrations of 0.004 and 0.020 a.i. g/L were prepared to soak plum fruits for two minutes. After drying naturally, the fruits were placed in polypropylene boxes, lidded, and stored at low temperature (4 ± 1) °C and room temperature (20 ± 1) °C, respectively. Under the low and room temperature conditions, the control groups (clear water) and different concentrations of propiconazole treatment groups were each set with three replicates, and the weight of plums in each replicate treatment group was 4 kg. The sampling time of plums stored at 20 °C was 2 h, 5, 10, 15, 20, 25, and 30 days, and the sampling time of plums stored at 4 °C was 2 h, 5, 10, 15, 20, 25, 35, 45 and 55 days. Samples were taken at regular intervals, and five plums were randomly selected from each treatment group and used to determine the plum’s hardness and total soluble solids content; eight plum samples from each treatment group were randomly selected and were chopped and stored at −20 °C in time for the subsequent the determination of propiconazole stereoisomer residues.

### 2.3. Sample Extraction

**Figure 2:**
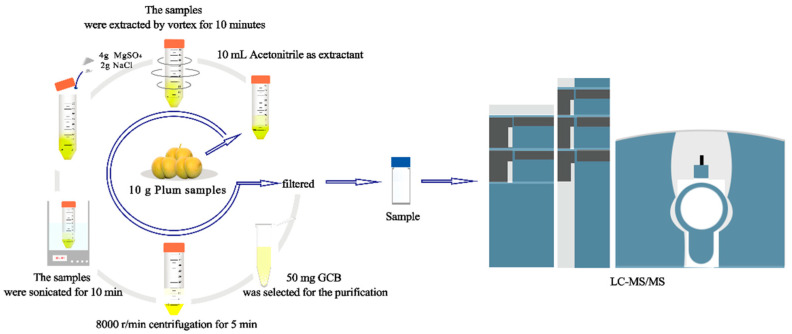
Flow chart of the methodological process.

Firstly, “Fengtang” plum samples (10 g) were weighed in a 50 mL centrifuge tube and mixed with 10 mL of acetonitrile, which was extracted by vortex for 10 min. After that, 4 g of MgSO_4_ and 1 g NaCl were added and sonicated at room temperature for 10 min. Then, the samples were centrifuged for 5 min at 8000 r/min and 1 mL supernatant was purified with 50 mg GCB. Finally, the purified extract was filtered through a 0.22 μm nylon syringe filter for the analysis of residues. The “March” and “Carmine” samples were extracted and purified in the same manner as the “Fengtang” plum samples. Figure 2 shows the flow chart of the procedures. 

### 2.4. Instrumental Methods

The instrumental methods of Tang et al. were used [26], with modifications, to determine propiconazole stereoisomer residues in plum. By using HPLC−MS/MS equipped with a Superchiral-S-OX chiral column (250 mm × 4.6 mm, 3 μm, Chiralway Biotech Co., Ltd., Shanghai, China). Ethanol containing 1%(*v*/*v*) formic acid was used as the mobile phase, the flow rate was 0.25 mL/min, and the injection volume was 10 μL. Other parameters set are shown in Table S1 in Supplementary Materials.

### 2.5. Fruit Firmness

A firmness tester (GY-5B, Aipi, Nantong, China) was purchased and used to measure the firmness of “Fengtang” plum samples. The firmness was determined from two different locations of each plum and the reading (kg/cm^2^) was recorded. About ten measurements were performed for each experimental treatment group and their average data were obtained.

### 2.6. Total Soluble Solids (TSS)

TSS was measured using a manual sugar-acid all-in-one machine (PAL-BX/ACID 11, ATAGO Technology Co., Ltd., Xiamen, China). The determination of TSS is to take 2 g of plum pulp and grind it evenly, take 1.0 mL of juice and drop it into the prism groove, and record the indicated number. TSS was expressed as a percentage (%).

### 2.7. Data Analysis

#### 2.7.1. Dissipation Kinetics

The residual amount of propiconazole enantiomers was used as the ordinate and time as the abscissa to plot the degradation curve in plum. We calculated the rate constant k corresponding to propiconazole enantiomers using the first-order kinetic equation (Equation (1)) [34]. The half-life of propiconazole enantiomers in plum was determined using Equation (2):

C_t_ = C_0_e^−kt^ (1)

t_1/2_ = ln2/k = 0.693/k (2)

Here, C_t_ stands for the concentration of the propiconazole enantiomers in the sample at time t (day), C_0_ represents the initial concentration of the propiconazole enantiomers in the sample 2 h after application, and k is the degradation rate constant [34,35]. 

#### 2.7.2. Matrix Effect

The matrix effects (ME) of the plum in this experiment were calculated according to the following Equation (3):

ME (%) = 100% × [slope_(matrix)_ − slope_(solvent)_]/slope_(solvent)_ (3)

The slopes of the calibration curves for the matrix and solvent standards are indicated here as the slope_(matrix)_ and slope_(solvent)_, respectively [35]. The three categories of the matrix effect are weak or no matrix effect, medium matrix effect, and strong matrix effect, and their corresponding values are |ME| ≤ 20%, 20% < |ME| < 50%, and |ME| ≥ 50%, respectively.

#### 2.7.3. The Enantiomer Fraction (EF) Calculated

All chiral pesticides may have stereoselective degradation, so Equations (4) and (5) were used to calculate the propiconazole enantiomeric fraction (EF). The EF value can be used to quantify the enantiomeric composition of propiconazole in plums stored at 20 °C and 4 °C.

EFa = C_(2R,4S)_/[C_(2R,4S)_ + C_(2S,4R)_] (4)

EFa = C_(2R,4R)_/[C_(2R,4R)_ + C_(2S,4S)_] (5)

Here, EFa and EFb represent the enantiomeric fraction of enantiomers cis A and trans B, and EF values were in the range of 0 to 1, and a value of EF equal to 0.5 indicated the racemic mixture. C_(2R,4S)_, C_(2S,4R)_, C_(2R,4R)_, and C_(2S,4S)_ represent the concentrations of (2R,4S)-propiconazole, (2S,4R)-propiconazole, (2R,4R)-propiconazole, and (2S,4S)-propiconazole, respectively [26,28].

### 2.8. Statistical Analysis

All values are presented as mean ± S.D. Microsoft Excel 2021 and Origin 2021 were used to sort out the experimental data and draw graphs.

## 3. Results and Discussion

### 3.1. Optimization of Extractants and Purify Agents

The extraction effects of several common solutions on propiconazole (methanol, acetonitrile, acetonitrile with 1% formic acid) from the plum matrix were examined in this experiment. According to the test results (Figure S1), at the total additional level of 0.2 mg/kg, acetonitrile 1% formic acid, methanol, and acetonitrile extracted four propiconazole stereoisomers well, with recoveries of 78.02–85.40%, 89.92–90.87%, 91.15–96.65%, and RSDs ≤ 4.59%. Because of methanol’s relatively strong polarity, the plum-extracted colors with methanol were much deeper than that with acetonitrile. Therefore, acetonitrile was chosen as the propiconazole extraction agent in this experiment.

Three commonly used dispersive solid-phase extraction sorbents (C18, PSA, GCB) in the “Fengtang” plum matrix were also investigated to evaluate the purification effect. Acetonitrile was used as an extractant, then the extract was purifiers purified with 50 mg C18, 50 mg PSA, and 50 mg GCB, respectively. The results showed that the color of the extractant purified by 50 mg GCB was the lightest, and the corresponding recovery rate was satisfied after detection. Therefore, 50 mg GCB was finally selected as the purifier in this experiment.

### 3.2. Method Verification Result

This experiment’s method of propiconazole enantiomer residue analysis in plum was validated, and the results of the experiment could be valuable and useful. Calculations were made for linearity, matrix effect, the limit of quantification (LOQ), the limit of detection (LOD), precision, and recovery. To evaluate the linearity of the method used in this experiment, 1 mL of the blank plum extract solution was dried at 45 °C before being redissolved with 1 mL of different concentrations of propiconazole standard solution (0.02–4 μg/mL), the matrix standard solution could be obtained. As shown in Table 1, the linear was good in plum (R^2^ > 0.9987, 0.02–4 μg/mL). Equation (3) was used to determine the matrix effects of plum matrices. Propiconazole enantiomers have different matrix effects in plum matrices, the matrix effects ranged from 0.55 to 0.76, indicating that plum had a strong matrix effect. Therefore, to obtain more accurate quantification data for this investigation, we use a matrix-matched standard. The lowest spiked level was designated as the LOQ, and the LOD value was determined using a signal-to-noise ratio of 3:1. According to estimates, the LODs of the propiconazole enantiomers in plum were 0.0005 mg/kg, and the LOQs were 0.004–0.006 mg/kg. 

**Figure 3:**
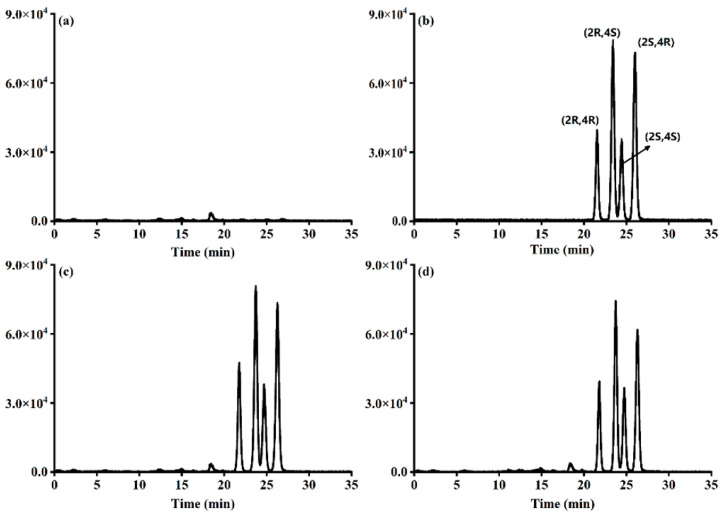
Typical LC-MS/MS chromatograms of propiconazole: (**a**) blank plum; (**b**) solvent standard solution (0.2 μg/mL); (**c**) matrix standard solution (0.2 μg/mL); (**d**) addition (0.2 mg/kg).

Intraday and interday recovery experiments were performed with five replicates per level for three consecutive days, which were used to evaluate the method’s accuracy and precision in this study. The propiconazole enantiomer mean recovery in the plum matrix ranged from 79.42 to 104.05% at various levels of addition (0.02, 0.2, and 2 mg/kg), and the relative standard deviation (RSD) was 1.54–11.68% (Table 2). The results show that our method has good accuracy and precision in plum and can successfully meet the needs of propiconazole enantiomers residue detection in plum matrices. Referring to Tang et al., under the chromatographic conditions described in this paper, the peak sequence of four stereoisomers of propiconazole was (2R,4R)-propiconazole, (2R,4S)-propiconazole, (2S,4S)-propiconazole, and (2S,4R)-propiconazole, respectively [26]. Figure 3 showed the typical chromatograms of four propiconazole stereoisomers, and the retention times were 21.77, 23.66, 24.63, and 26.25 min, respectively.

To verify the generality of this method, the recovery rates of propiconazole in the matrices of two other plum varieties (“March” and “Carmine” plum) were determined. The relevant results are provided in Table S2 in Supplementary Materials. The results showed that propiconazole has a good recovery rate in the “March” and “Carmine” plum matrices. Therefore, the proposed method could be applied to other plum varieties.

### 3.3. Dissipation Kinetics of Propiconazole Enantiomers in Plum

We investigated the degradation of four enantiomers of propiconazole in plums at 20 °C and 4 °C during plum storage. The degradation kinetics of the four propiconazole stereoisomers in plums are shown in Table 3 and Figure S2. As can be seen from the data in the table, the trends of the four stereoisomers of propiconazole in plums at 20 °C and 4 °C followed first-order kinetics. (2R,4R)-propiconazole, (2R,4S)-propiconazole, (2S,4S)-propiconazole, and (2S,4R)-propiconazole had half-lives of 12.60, 15.40, 9.49, and 13.32 d, respectively, in plums treated with propiconazole at 0.004 a.i. g/L at 20 °C; half-lives of 28.88, 23.90, 27.72, and 23.90, respectively, in plums treated with propiconazole at 0.004 a.i. g/L at 4 °C; half-lives of 12.60, 13.86, 10.34, and 13.08 d, respectively, in plums treated with propiconazole at 0.020 a.i. g/L at 20 °C; and half-lives of 24.75, 23.90, 23.90, and 21.00, respectively, in plums treated with propiconazole at 0.020 a.i. g/L at 4 °C. By comparison, we found that the half-life of the four stereoisomers was not affected by the treatment concentration. However, the storage temperature was an important factor affecting the half-life of the four stereoisomers of propiconazole, the lower the storage temperature of the plum, the longer the half-life of propiconazole enantiomers. It may be that high temperatures accelerated the volatilization of propiconazole in plums, and the high activity of enzymes at high temperatures also leads to the rapid degradation of propiconazole in plums.

**Figure 4:**
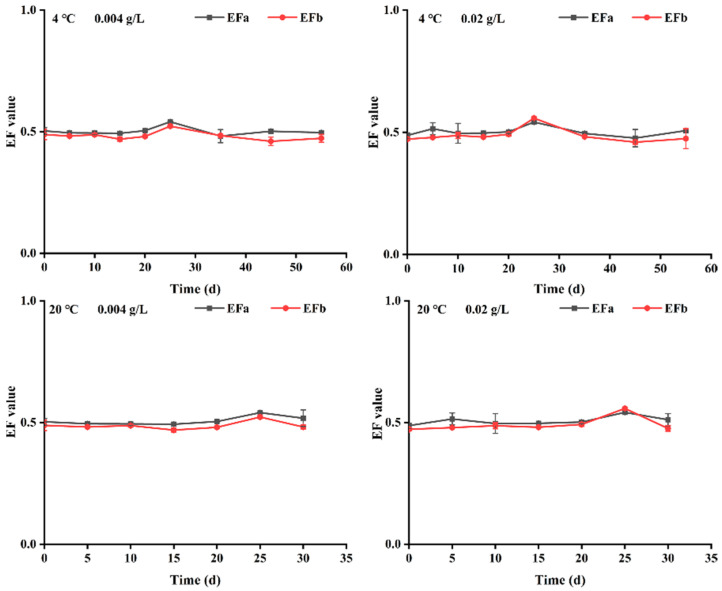
Temporal development of EFa and EFb values for the propiconazole diastereomers in plums treated with two concentrations at 20 °C and 4 °C.

EF values of two pairs of propiconazole enantiomers in stored plums were calculated according to Equations (4) and (5), respectively. Additionally, under the same temperature condition, the change trend of EF values of the two concentration treatment groups was similar as a whole (Figure 4). The EFa and EFb values were close to 0.5 for most sampling times after treatment with propiconazole at both temperatures and concentrations. At 4 °C, EFa and EFb values of plums treated with propiconazole were in the range of 0.47–0.54 and 0.45–0.55, respectively; at 20 °C, EFa and EFb values were 0.48–0.54 and 0.46–0.55, respectively. These data indicate that the degradation of two pairs of propiconazole enantiomers was slightly enantioselective in the stored plum. (2R,4R)-propiconazole and (2R,4S)-propiconazole degraded slightly slower than their corresponding enantiomers (2S,4S)-propiconazole and (2S,4R)-propiconazole in the stored plums. This result is similar to that of Tang et al., who found that (2S,4S)-propiconazole and (2S,4R)-propiconazole degraded slightly faster than their corresponding enantiomers (2R,4R)-propiconazole and (2R,4S)-propiconazole in banana leaves [26].

### 3.4. Final Residues of Propiconazole Enantiomers in Plum

In this experiment, the residues of propiconazole enantiomers in the plums during storage at 20 °C and 4 °C are shown Table 4. The initial total residues of propiconazole in plums in propiconazole −0.004, 0.020 treatment groups were 0.274 and 0.487 mg/kg, respectively. The contents of four stereoisomers in plums decreased gradually with the extension of storage time and a high concentration of application and low temperature would lead to a large residue of propiconazole in plums. For example, at the 20th day of storage, the residues of (2R,4R)-propiconazole, (2R,4S)-propiconazole, (2S,4S)-propiconazole, and (2S,4R)-propiconazole were 0.020, 0.021, 0.020, and 0.018 mg/kg, respectively, in plums treated with propiconazole at 0.004 a.i. g/L at 20 °C; the residues were 0.040, 0.045, 0.044, and 0.044 mg/kg, respectively, in plums treated with propiconazole at 0.004 a.i. g/L at 4 °C; the residues were 0.040, 0.042, 0.042, and 0.038 mg/kg, respectively, in plums treated with propiconazole at 0.020 a.i. g /L at 20 °C; and the residues were 0.094, 0.103, 0.097, and 0.102 mg/kg, respectively, in plums treated with propiconazole at 0.020 a.i. g /L at 4 °C. Due to the enantioselective degradation of the two pairs of enantiomers being slight, the residues of (2R,4R)-propiconazole and (2S,4S)-propiconazole were approximately the same at most sampling points. Moreover, the residues of (2R,4S)-propiconazole and (2S,4R)-propiconazole were also approximately the same. At the end of the storage period, the total residues of propiconazole in plums at 20 °C and 4 °C temperatures were 0.026–0.108 mg/kg and 0.086–0.153 mg/kg, respectively. Compared with the MRL value, the results were less than the MRL (0.6 mg/kg). The LC chromatograms of some samples are shown in the supporting information in Figures S3–S34.

### 3.5. Effect of Cleaning on Propiconazole Total Residue in Plum

Plums were collected to be washed with tap water at the end of the storage period to investigate the effect of cleaning on propiconazole residues. Plums treated with different concentrations of propiconazole were rinsed with running water for 5 to 7 s with a gentle hand rub, and residual propiconazole in cleaned plums and unwashed plums was detected. As can be seen from Table 5, the initial residues were 0.086 and 0.154 mg/kg, respectively, and the residues after cleaning were 0.039 and 0.078 mg/kg, respectively, which obviously reduced the residue in plum after washing. The removal rate of total residual propiconazole in plums is 49.35–54.65%. Therefore, cleaning is an effective way to reduce pesticide intake.

### 3.6. Changes in the Firmness of Plums

**Figure 5:**
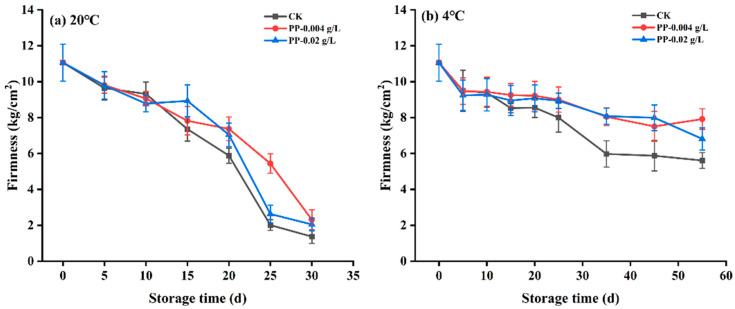
Effects of different treatments on the firmness of “Fengtang” plum during storage at 20 °C (**a**) and 4 °C (**b**). (CK is the control group, and PP is propiconazole, n = 3).

Fruit gradually softened during storage and preservation; therefore, higher hardness values during storage indicate good quality during storage [36]. As shown in Figure 5, the fruit firmness of each treatment group of samples decreased with storage time under both room temperature (20 °C) and low temperature (4 °C). After 10 days of storage at 4 °C, plums were harder than those at 20 °C. This also implies that low temperatures may postpone the softening of the fruit to some extent and it can be seen from the figure that with the increase in storage time, the hardness of plums treated with propiconazole was significantly higher than that of the control group at both temperatures, indicating that propiconazole treatment had a better impact on the hardness retention of “Fengtang” plums.

### 3.7. Changes in the Total Soluble Solids Content of Plums

**Figure 6:**
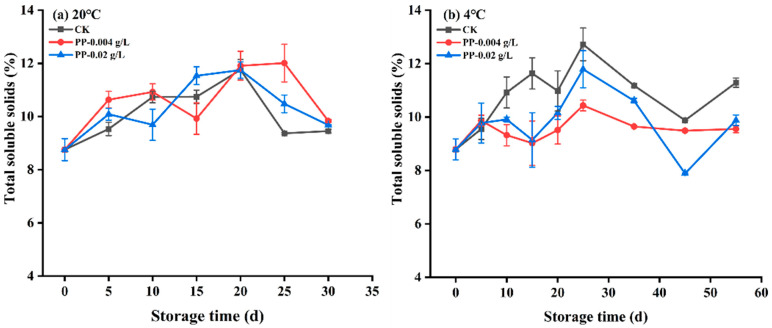
Effects of different treatments on the total soluble solids content of “Fengtang” plum during storage at 20 °C (**a**) and 4 °C (**b**). (CK is the control group, and PP is propiconazole, n = 3).

The total soluble solids (TSS) content (mainly including soluble sugar) in fruit samples can directly reflect the ripening degree and fruit quality [37,38]. During storage, the TSS content increases as the fruit matures and decreases during aging [38]. Therefore, TSS content can reflect whether a storage process can delay fruit ripening and prolong fruit storage time. As shown in Figure 6, under room-temperature (20 °C) and low-temperature (4 °C) storage, the TSS content of plums in each treatment group increased initially and then decreased somewhat throughout the storage period. Moreover, at 20 °C, the TSS content in the control group was 8.79–11.75%, and the content in the propiconazole treatment groups was in the range of 8.79–12.01%, there was little difference in the TSS content range between the control group and treatment group. At 4 °C, the TSS content was obviously lower in plums treated with propiconazole than in the control group at 10–55 days of storage, this may be due to the inhibition of plums ripening with propiconazole at low temperatures.

## 4. Conclusions

This study establishes an accurate and sensitive analytical technique for detecting the four stereoisomers of propiconazole in plums. This technique uses LC-MS/MS on the chiral level and is suitable for determining the residues of four propiconazole stereoisomers in different kinds of plums. The half-lives of the four stereoisomers in the stored plums were 9.49–15.40 days at 20 °C and 21.00–28.88 days at 4 °C. The four stereoisomers showed an extremely low level of stereoselective degradation in “Fengtan” plum. The residues of the four propiconazole stereoisomeric in plums decreased gradually with the storage time, and the residues at 20 °C were significantly lower than those at 4 °C. The residue at the two doses did not exceed the MRL, and the residues of propiconazole were removed by cleaning. The firmness and TSS content showed that propiconazole treatment can effectively maintain the firmness of plums to a certain extent and might have an inhibitory effect on postharvest ripening. We further explored the impact of propiconazole and other chemical fungicides on the quality of stored “Fengtan” plum, such as vitamin content, titratable acid content, and moisture content. Some effective and safe chemical fungicides that extend the shelf life of “Fengtan” plum are recommended. These fungicides are of great significance because they improve the economic value of “Fengtang” plums and increase the income of local farmers in Guizhou, China.

## Figures and Tables

**Table 1:** Linear equation, correlation coefficient (R^2^), ME, detection limit, and quantification limit of propiconazole enantiomer in “Fengtang” plum matrix.

Matrices	Analytes	Linear Equation	SD *	Correlation R^2^	Concentration (µg/mL) ^a^	Matrix Effects (ME)	LOD ^b^ (mg/kg)	LOQ ^b^ (mg/kg) ^b^
Acetonitrile	(2R,4R)-propiconazole	y = 3.92 × 10^6^x − 55468	1.42 × 10^5^	0.9958	0.02–4			
	(2R,4S)-propiconazole	y = 9.21 × 10^6^x − 37816	2.08 × 10^5^	0.9993				
	(2S,4S)-propiconazole	y = 3.97 × 10^6^x − 47049	2.02 × 10^4^	0.9982				
	(2S,4R)-propiconazole	y = 9.32 × 10^6^x − 78100	5.03 × 10^4^	0.9986				
Fengtang plum	(2R,4R)-propiconazole	y = 6.89 × 10^6^x − 123123	2.23 × 10^5^	0.9991	0.02–4	0.76	0.0005	0.004
	(2R,4S)-propiconazole	y = 1.54 × 10^7^x − 311511	5.31 × 10^5^	0.9989		0.67	0.0005	0.006
	(2S,4S)-propiconazole	y = 6.15 × 10^6^x − 121224	9.11 × 10^4^	0.9987		0.55	0.0005	0.004
	(2S,4R)-propiconazole	y = 1.56 × 10^7^x − 307315	4.36 × 10^5^	0.9991		0.67	0.0005	0.006

^a^ The sum of (2R,2R)-propiconazole, (2R,4S)-propiconazole, (2S,4S)-propiconazole and (2S,4R)-propiconazole. The ratio of (2R,2R)-propiconazole, (2R,4S)-propiconazole, (2S,4S)-propiconazole, and (2S,4R)-propiconazole was 0.20:0.30:0.20:0.30 in the standard solution of propiconazole. ^b^ Single stereoisomer of propiconazole. * Denotes the SD value of the coefficient of the linear equation.

**Table 2:** Recovery rates and relative standard deviations (intraday and interday) of propiconazole enantiomers in plums.

		Average Addition Recovery Rate (%), Intraday RSD (%, n = 5)						
Analytes	Add Level (mg/kg) ^a^	Day 1		Day 2		Day 3		Interday RSD (%, n = 15)
(2R,4R)-propiconazole	0.02	91.16	4.71	92.13	1.17	79.42	3.64	7.23
	0.20	96.01	3.17	76.27	4.65	99.41	4.69	11.68
	2.00	92.88	4.29	81.23	1.83	98.12	1.90	8.07
(2R,4S)-propiconazole	0.02	94.19	6.71	95.03	1.98	82.81	4.51	7.58
	0.20	96.58	4.22	81.11	3.12	100.27	5.45	10.12
	2.00	97.85	3.89	88.23	1.16	101.66	2.34	6.31
(2S,4S)-propiconazole	0.02	92.28	5.83	96.86	1.36	81.19	4.69	8.21
	0.20	95.62	3.98	86.59	4.31	104.10	3.40	8.22
	2.00	93.06	4.74	91.60	1.61	100.38	2.47	5.01
(2S,4R)-propiconazole	0.02	91.73	4.04	95.00	1.51	80.12	6.84	8.16
	0.20	97.29	2.57	82.41	1.71	104.05	4.94	9.90
	2.00	95.66	4.15	86.02	1.54	98.25	1.64	6.09

^a^ The sum of (2R,2R)-propiconazole, (2R,4S)-propiconazole, (2S,4S)-propiconazole, and (2S,4R)-propiconazole.

**Table 3:** The degradation equation of propiconazole enantiomers in plum.

		Storage Temperature 20 °C				Storage Temperature 4 °C			
Treatment Group (g/L)	Analytes	Regressive Function	R^2^	T_1/2_ (d)	SD (d)	Regressive Function	R^2^	T_1/2_ (d)	SD (d)
0.004	(2R,4R)-propiconazole	C_t_ = 0.0548e^−0.055t^	0.7491	12.60	0.58	C_t_ = 0.0636e^−0.024t^	0.9007	28.88	2.69
	(2R,4S)-propiconazole	C_t_ = 0.0542e^−0.045t^	0.7532	15.40	0.63	C_t_ = 0.0751e^−0.029t^	0.9365	23.90	2.35
	(2S,4S)-propiconazole	C_t_ = 0.0683e^−0.073t^	0.7588	9.49	0.61	C_t_ = 0.0623e^−0.025t^	0.7455	27.72	2.72
	(2S,4R)-propiconazole	C_t_ = 0.0537e^−0.052t^	0.7535	13.32	0.94	C_t_ = 0.0742e^−0.029t^	0.9026	23.90	1.72
0.020	(2R,4R)-propiconazole	C_t_ = 0.1330e^−0.055t^	0.7489	12.60	0.96	C_t_ = 0.1553e^−0.028t^	0.9455	24.75	1.20
	(2R,4S)-propiconazole	C_t_ = 0.1347e^−0.05t^	0.7339	13.86	1.08	C_t_ = 0.1637e^−0.029t^	0.8988	23.90	3.17
	(2S,4S)-propiconazole	C_t_ = 0.165e^−0.067t^	0.7923	10.34	0.46	C_t_ = 0.1658e^−0.029t^	0.9395	23.90	2.33
	(2S,4R)-propiconazole	C_t_ = 0.1372e^−0.053t^	0.7685	13.08	1.14	C_t_ = 0.1701e^−0.033t^	0.9156	21.00	0.88

**Table 4:** Residues of propiconazole stereoisomers in plums during storage (n = 3).

Storage Temperature	Treatment Group (g/L)	Time (Days)	(2R,4R) (mg/kg)	(2R,4S) (mg/kg)	(2S,4S) (mg/kg)	(2S,4R) (mg/kg)	Total Value (mg/kg)
20 °C	0.004	10	0.031 ± 0.002	0.033 ± 0.002	0.035 ± 0.002	0.030 ± 0.002	0.129
		20	0.020 ± 0.001	0.021 ± 0.001	0.020 ± 0.003	0.018 ± 0.001	0.079
		30	0.006 ± 0.002	0.009 ± 0.003	0.004 ± 0.001	0.009 ± 0.002	0.026
	0.020	10	0.078 ± 0.007	0.109 ± 0.007	0.093 ± 0.005	0.106 ± 0.006	0.386
		20	0.040 ± 0.002	0.042 ± 0.003	0.042 ± 0.003	0.038 ± 0.003	0.163
		30	0.026 ± 0.014	0.035 ± 0.017	0.018 ± 0.010	0.030 ± 0.014	0.108
4 °C	0.004	10	0.058 ± 0.003	0.064 ± 0.005	0.061 ± 0.004	0.065 ± 0.004	0.247
		20	0.040 ± 0.008	0.045 ± 0.008	0.044 ± 0.008	0.044 ± 0.008	0.173
		55	0.020 ± 0.002	0.018 ± 0.002	0.030 ± 0.003	0.018 ± 0.002	0.086
	0.020	10	0.134 ± 0.010	0.131 ± 0.009	0.142 ± 0.014	0.134 ± 0.020	0.540
		20	0.094 ± 0.007	0.103 ± 0.010	0.097 ± 0.006	0.102 ± 0.009	0.397
		55	0.046 ± 0.014	0.041 ± 0.013	0.038 ± 0.011	0.029 ± 0.014	0.153

**Table 5:** Effect of cleaning on propiconazole enantiomers residues in plum.

Treatment Group (g/L)	Samples	(2R,4R) (mg/kg)	(2R,4S) (mg/kg)	(2S,4S) (mg/kg)	(2S,4R) (mg/kg)	Total Value (mg/kg)	Reduction Ratio (%)
0.004	Unprocessed	0.020 ± 0.002	0.018 ± 0.002	0.030 ± 0.003	0.018 ± 0.002	0.086	
	Cleaning	0.011 ± 0.001	0.009 ± 0.004	0.011 ± 0.003	0.008 ± 0.001	0.039	54.65
0.020	Unprocessed	0.046 ± 0.014	0.041 ± 0.013	0.038 ± 0.011	0.029 ± 0.014	0.154	
	Cleaning	0.019 ± 0.010	0.019 ± 0.001	0.021 ± 0.012	0.019 ± 0.008	0.078	49.35

## Data Availability

Data is contained within the article (or supplementary material).

## Supplementary Materials

The following supporting information can be downloaded at: https://www.mdpi.com/article/10.3390/foods12112200/s1.
