# Supplementary material for: Degradation Dynamics and Residue Analysis of Four Propiconazole Stereoisomers in “Fengtang” Plum during Storage by LC-MS/MS
Source: Foods. 2023 May 30;12(11):2200. doi: 10.3390/foods12112200 (PMC10253140; doi:10.3390/foods12112200)
Supplement: Supplementary file 1 [file foods-12-02200-s001.zip › foods-2400936-supplementary.pdf]

# SUPPORTTING INFORMATION

# Degradation Dynamics and Residue Analysis of Four Propiconazole Stereoisomers in "Fengtang" Plum during Storage by LC-MS/MS

**Pengyu Deng[1], Lianhong Mou [1], Guipeng Ou [1], Xin Luo [1], Deyu Hu [1], Yuping Zhang [1*]**

[1] National Key Laboratory of Green Pesticide, Key Laboratory of Green Pesticide and Agricultural Bioengineering, Ministry of Education, Center for R&D of Fine Chemicals of Guizhou University, Guiyang 550025, China

* Correspondence: zhangyupinggz@163.com; Tel.: (+86)851 88292090

Table of Content
Table _1_SuppInfo.

**Table S1.** The parameters of the instruments for propiconazole

| Parameter Name | Information |
|---|---|
| Ion source | ESI |
| Quantitative ion pair | 342.0/159.1 m/z |
| Qualitative ion pair | 342.0/69.1 m/z |
| Column temperature | 35°C |
| Ion source temperature | 500°C |
| Ion sprayer voltage | 5500 V |
| Ion source gas (GS1) | 55.0 psi |
| Ion source gas (GS2) | 55.0 psi |
| Curtain Gas | 35.0 psi |
| Scan mode | Positive |
| Scan patterns | Multiple-scanning |

Table of Content
Table _2_SuppInfo.

**Table S2**. Recovery rates and relative standard deviations of propiconazole enantiomers in "March" and "Carmine" plum

| Plum variety | Analytes | Add level (mg/kg) [a] | Recovery rate (%) | | | | | | RSD (%, n = 5) |
|---|---|---|---|---|---|---|---|---|---|
| | | | 1 | 2 | 3 | 4 | 5 | Average | |
| March plum | (2R,4R)-propiconazole | 0.02 | 94.08 | 101.56 | 87.85 | 84.74 | 84.11 | 90.47 | 7.35 |
| | | 0.20 | 98.96 | 98.96 | 88.31 | 87.06 | 89.56 | 92.57 | 5.90 |
| | | 2.00 | 96.57 | 98.86 | 97.14 | 93.14 | 88.57 | 94.86 | 4.08 |
| | (2R,4S)-propiconazole | 0.02 | 93.24 | 95.37 | 91.81 | 96.09 | 98.93 | 95.09 | 2.74 |
| | | 0.20 | 102.7 | 102.0 | 96.03 | 94.54 | 99.01 | 98.86 | 3.59 |
| | | 2.00 | 96.53 | 102.8 | 100.3 | 90.22 | 88.96 | 95.77 | 6.09 |
| | (2S,4S)-propiconazole | 0.02 | 86.21 | 87.59 | 81.38 | 82.76 | 91.03 | 85.79 | 3.86 |
| | | 0.20 | 94.35 | 94.35 | 99.19 | 103.2 | 100.0 | 98.23 | 3.84 |
| | | 2.00 | 96.40 | 105.0 | 104.3 | 91.37 | 93.53 | 98.13 | 6.24 |
| | (2S,4R)- propiconazole | 0.02 | 92.50 | 95.63 | 78.13 | 91.88 | 86.88 | 89.00 | 6.84 |
| | | 0.20 | 96.53 | 93.06 | 93.06 | 83.33 | 92.36 | 91.67 | 4.93 |
| | | 2.00 | 96.30 | 103.1 | 103.7 | 94.44 | 91.98 | 97.90 | 5.25 |
| Carmine plum | (2R,4R)-propiconazole | 0.02 | 84.98 | 83.50 | 92.36 | 96.06 | 93.84 | 90.15 | 5.58 |
| | | 0.20 | 92.65 | 102.94 | 87.50 | 105.15 | 96.32 | 96.91 | 7.27 |
| | | 2.00 | 101.9 | 103.4 | 105.6 | 100.0 | 94.97 | 101.1 | 4.01 |
| | (2R,4S)-propiconazole | 0.02 | 90.28 | 88.19 | 81.94 | 97.22 | 98.61 | 91.25 | 6.83 |
| | | 0.20 | 85.99 | 94.27 | 105.1 | 106.4 | 100.0 | 98.34 | 8.40 |
| | | 2.00 | 93.60 | 101.2 | 98.84 | 100.0 | 97.09 | 98.14 | 2.95 |
| | (2S,4S)-propiconazole | 0.02 | 91.60 | 94.66 | 88.55 | 90.84 | 82.44 | 89.62 | 4.57 |
| | | 0.20 | 83.69 | 91.49 | 99.29 | 97.87 | 86.52 | 91.77 | 6.83 |
| | | 2.00 | 102.0 | 101.9 | 102.6 | 95.42 | 94.77 | 99.35 | 3.89 |
| | (2S,4R)-propiconazole | 0.02 | 91.41 | 90.18 | 100.6 | 97.55 | 83.44 | 92.64 | 6.71 |
| | | 0.20 | 88.89 | 99.31 | 104.9 | 102.1 | 95.83 | 98.19 | 6.18 |
| | | 2.00 | 100.6 | 96.63 | 103.4 | 101.1 | 98.31 | 100.0 | 2.60 |

[a] The sum of (2R,2R)-propiconazole, (2R,4S)-propiconazole, (2S,4S)-propiconazole, and (2S,4R)-propiconazole.

Figure Caption
Figure_1_SuppInfo.

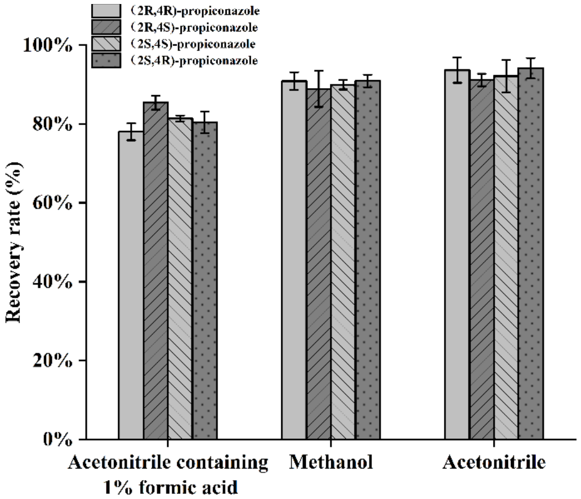


**Figure S1** Effects of different extractants on recoveries of propiconazole stereoisomers

Figure Caption
Figure_2_SuppInfo.

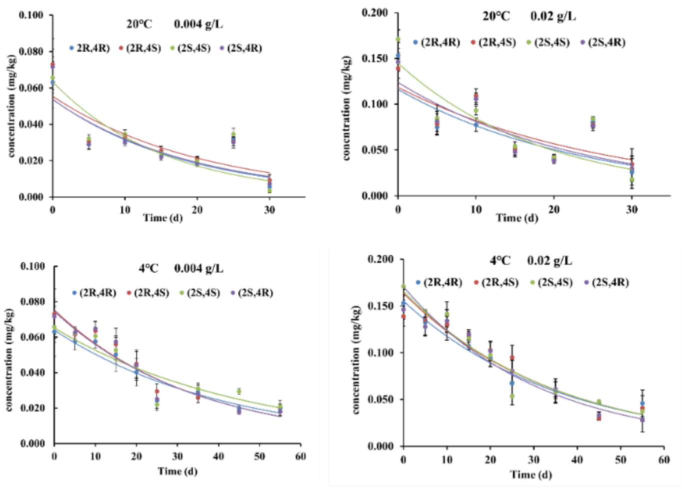


**Figure S2.** Digestion dynamics curves of propiconazole stereoisomers in plums at 4 and 20 °C

Figure Caption
Figure_3_SuppInfo.

| | | | |
|---|---|---|---|
| **Data File** | 20220822 BHZ-YP-4du.wiff | **Result Table** | 4-0.004-0d-1.rdb |
| **Acquisition Date** | 8/23/2022 5:22:46 AM | **Algorithm Used** | MQL |
| **Acquisition Method** | BHZ  method.dam | **Instrument Name** | AB SCIEX Triple Quad 4500 |
| **Project** | DPY | **Processing Method** | *No data for Processing Method* |

**Sample Name:** 0.004-0d-1 **Vial #:** 26

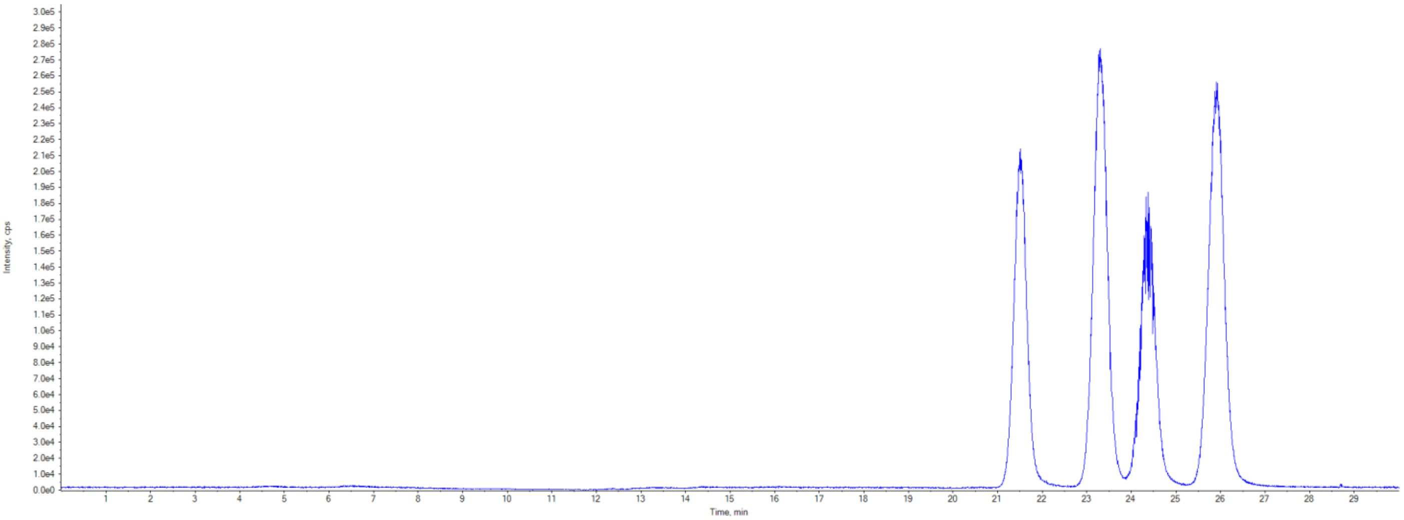


| | | | |
|---|---|---|---|
| **Sample Name** | 0.004-0d-1 | **Injection Vial** | 26 |
| **Data File** | 20220822 BHZ-YP-4du.wiff | **Injection Volume** | 10 |
| **Acquisition Date** | 8/23/2022 5:22:46 AM | **Algorithm Used** | MQL |
| **Acquisition Method** | BHZ  method.dam | **Sample Type** | Unknown |
| **Instrument Name** | AB SCIEX Triple Quad 4500 | **Result Table** | 4-0.004-0d-1.rdb |
| **Sample ID** | *No data for Sample ID* | **Dilution Factor** | 1.00 |
| **Sample Comment** | *No data for Sample Comment* | **Weight to Volume** | 0.00 |

Approved By (Date and Initials):_______________________________________________.

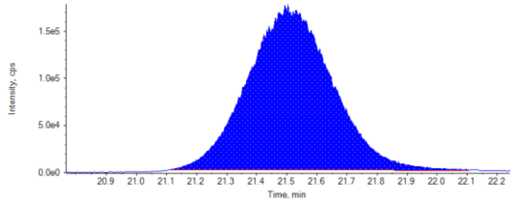


| **Compound Name:** | F1 **(**342.000/159.100 Da**)** |
|---|---|
| Expected RT: | 21.5 |
| Actual RT: | 21.5 |
| Equation: | At least 2 points are required to calculate regression. |
| Area Counts: | 3.56e+006 |
| ISTD Area Counts: | N/A |
| Amount: | 0.00 (ng/mL) |

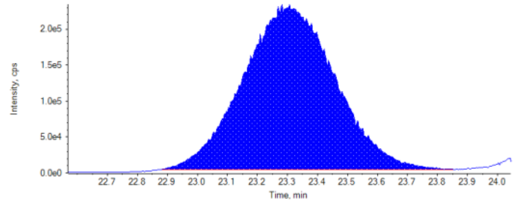


| **Compound Name:** | F2 **(**342.000/159.100 Da**)** |
|---|---|
| Expected RT: | 23.3 |
| Actual RT: | 23.3 |
| Equation: | At least 2 points are required to calculate regression. |
| Area Counts: | 5.00e+006 |
| ISTD Area Counts: | N/A |
| Amount: | 0.00 (ng/mL) |

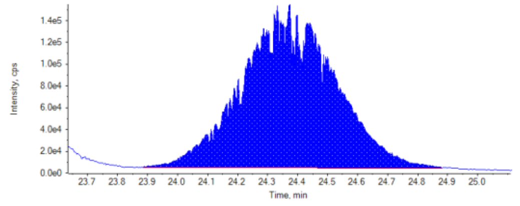


| **Compound Name:** | F3 **(**342.000/159.100 Da**)** |
|---|---|
| Expected RT: | 24.4 |
| Actual RT: | 24.4 |
| Equation: | At least 2 points are required to calculate regression. |
| Area Counts: | 3.10e+006 |
| ISTD Area Counts: | N/A |
| Amount: | 0.00 (ng/mL) |

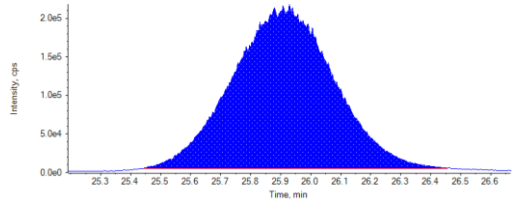


| **Compound Name:** | F4 **(**342.000/159.100 Da**)** |
|---|---|
| Expected RT: | 25.9 |
| Actual RT: | 25.9 |
| Equation: | At least 2 points are required to calculate regression. |
| Area Counts: | 5.22e+006 |
| ISTD Area Counts: | N/A |
| Amount: | 0.00 (ng/mL) |

Figure S3 The LC chromatogram of plums treated with 0.004 a.i. g/L propiconazole at 0 d

Figure Caption
Figure_4_SuppInfo.

| | | | |
|---|---|---|---|
| **Data File** | 20220822 BHZ-YP-4du.wiff | **Result Table** | 4-0.02-0d-3.rdb |
| **Acquisition Date** | 8/22/2022 5:50:38 PM | **Algorithm Used** | MQL |
| **Acquisition Method** | BHZ method.dam | **Instrument Name** | AB SCIEX Triple Quad 4500 |
| **Project** | DPY | **Processing Method** | *No data for Processing Method* |

**Sample Name:** 0.02-0d-3 **Vial #:** 4

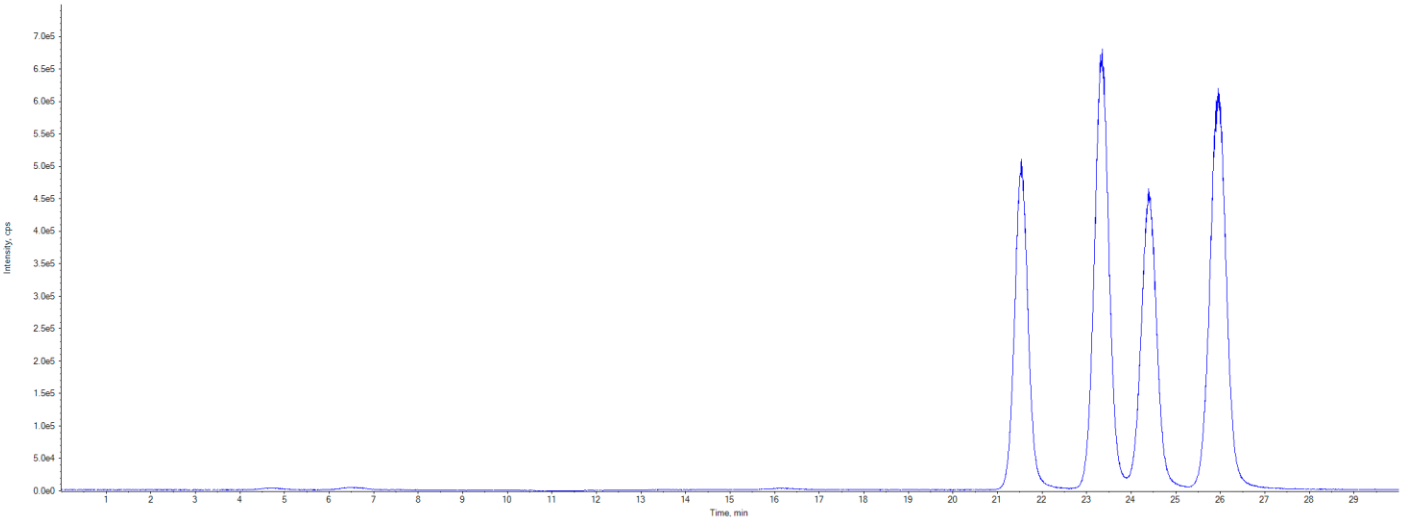


| | | | |
|---|---|---|---|
| **Sample Name** | 0.02-0d-3 | **Injection Vial** | 4 |
| **Data File** | 20220822 BHZ-YP-4du.wiff | **Injection Volume** | 10 |
| **Acquisition Date** | 8/22/2022 5:50:38 PM | **Algorithm Used** | MQL |
| **Acquisition Method** | BHZ method.dam | **Sample Type** | Unknown |
| **Instrument Name** | AB SCIEX Triple Quad 4500 | **Result Table** | 4-0.02-0d-3.rdb |
| **Sample ID** | *No data for Sample ID* | **Dilution Factor** | 1.00 |
| **Sample Comment** | *No data for Sample Comment* | **Weight to Volume** | 0.00 |

Approved By (Date and Initials):_______________________________________________.

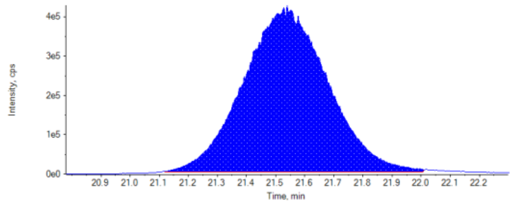


| **Compound Name:** | F1 **(**342.000/159.100 Da**)** |
|---|---|
| Expected RT: | 21.5 |
| Actual RT: | 21.5 |
| Equation: | At least 2 points are required to calculate regression. |
| Area Counts: | 8.80e+006 |
| ISTD Area Counts: | N/A |
| Amount: | 0.00 (ng/mL) |

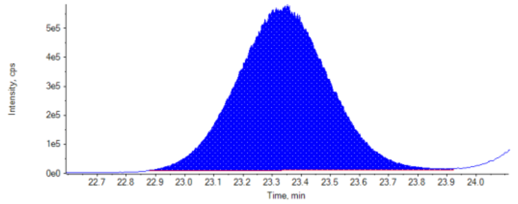


| | |
|---|---|
| **Compound Name:** | F2 **(**342.000/159.100 Da**)** |
| Expected RT: | 23.4 |
| Actual RT: | 23.4 |
| Equation: | At least 2 points are required to calculate regression. |
| Area Counts: | 1.26e+007 |
| ISTD Area Counts: | N/A |
| Amount: | 0.00 (ng/mL) |

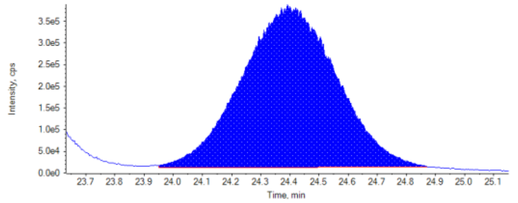


| | |
|---|---|
| **Compound Name:** | F3 **(**342.000/159.100 Da**)** |
| Expected RT: | 24.4 |
| Actual RT: | 24.4 |
| Equation: | At least 2 points are required to calculate regression. |
| Area Counts: | 8.60e+006 |
| ISTD Area Counts: | N/A |
| Amount: | 0.00 (ng/mL) |

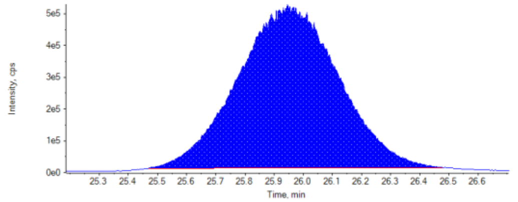


| | |
|---|---|
| **Compound Name:** | F4 **(**342.000/159.100 Da**)** |
| Expected RT: | 25.9 |
| Actual RT: | 25.9 |
| Equation: | At least 2 points are required to calculate regression. |
| Area Counts: | 1.31e+007 |
| ISTD Area Counts: | N/A |
| Amount: | 0.00 (ng/mL) |

Figure S4        Figure S4 The LC chromatogram of plums treated with 0.020 a.i. g/L propiconazole at 0 d

Figure Caption
Figure_5_SuppInfo.

| | | | |
|---|---|---|---|
| **Data File** | 20220820 BHZ-YP-20du.wiff | **Result Table** | 20-0.004-5d-2.rdb |
| **Acquisition Date** | 8/21/2022 2:34:46 PM | **Algorithm Used** | MQL |
| **Acquisition Method** | BHZ  method.dam | **Instrument Name** | AB SCIEX Triple Quad 4500 |
| **Project** | DPY | **Processing Method** | *No data for Processing Method* |

**Sample Name:** 0.004-5d-2 **Vial #:** 56

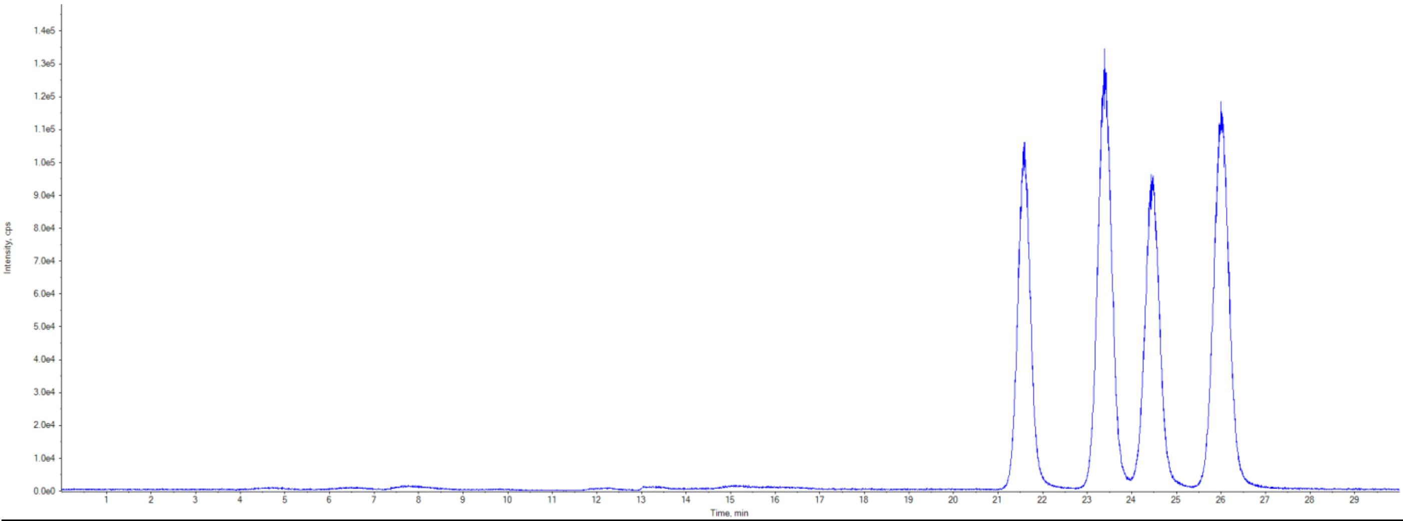


| | | | |
|---|---|---|---|
| **Sample Name** | 0.004-5d-2 | **Injection Vial** | 56 |
| **Data File** | 20220820 BHZ-YP-20du.wiff | **Injection Volume** | 10 |
| **Acquisition Date** | 8/21/2022 2:34:46 PM | **Algorithm Used** | MQL |
| **Acquisition Method** | BHZ  method.dam | **Sample Type** | Unknown |
| **Instrument Name** | AB SCIEX Triple Quad 4500 | **Result Table** | 20-0.004-5d-2.rdb |
| **Sample ID** | *No data for Sample ID* | **Dilution Factor** | 1.00 |
| **Sample Comment** | *No data for Sample Comment* | **Weight to Volume** | 0.00 |

Approved By (Date and Initials):________________________________________.

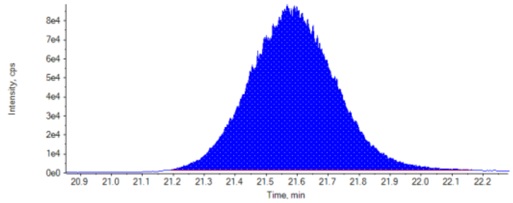


| **Compound Name:** | F1 **(**342.000/159.100 Da**)** |
|---|---|
| Expected RT: | 21.6 |
| Actual RT: | 21.6 |
| Equation: | At least 2 points are required to calculate regression. |
| Area Counts: | 1.72e+006 |
| ISTD Area Counts: | N/A |
| Amount: | 0.00 (ng/mL) |

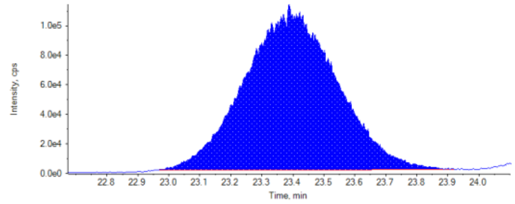


| **Compound Name:** | F2 (342.000/159.100 Da) |
|---|---|
| Expected RT: | 23.4 |
| Actual RT: | 23.4 |
| Equation: | At least 2 points are required to calculate regression. |
| Area Counts: | 2.31e+006 |
| ISTD Area Counts: | N/A |
| Amount: | 0.00 (ng/mL) |

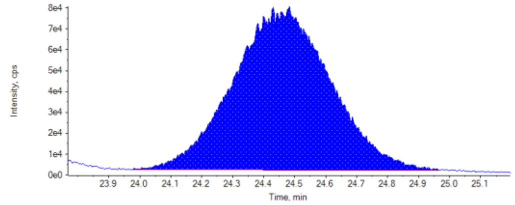


| **Compound Name:** | F3 (342.000/159.100 Da) |
|---|---|
| Expected RT: | 24.5 |
| Actual RT: | 24.5 |
| Equation: | At least 2 points are required to calculate regression. |
| Area Counts: | 1.72e+006 |
| ISTD Area Counts: | N/A |
| Amount: | 0.00 (ng/mL) |

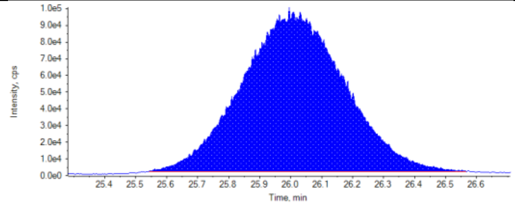


| **Compound Name:** | F4 (342.000/159.100 Da) |
|---|---|
| Expected RT: | 26.0 |
| Actual RT: | 26.0 |
| Equation: | At least 2 points are required to calculate regression. |
| Area Counts: | 2.33e+006 |
| ISTD Area Counts: | N/A |
| Amount: | 0.00 (ng/mL) |

Figure S5 The LC chromatogram of plums treated with 0.004 a.i. g/L propiconazole at 5 d (20℃)

Figure Caption
Figure_6_SuppInfo.

| | | | |
|---|---|---|---|
| **Data File** | 20220820 BHZ-YP-20du.wiff | **Result Table** | 20-0.004-10d-1.rdb |
| **Acquisition Date** | 8/21/2022 12:28:49 PM | **Algorithm Used** | MQL |
| **Acquisition Method** | BHZ method.dam | **Instrument Name** | AB SCIEX Triple Quad 4500 |
| **Project** | DPY | **Processing Method** | *No data for Processing Method* |

**Sample Name:** 0.004-10d-1 **Vial #:** 52

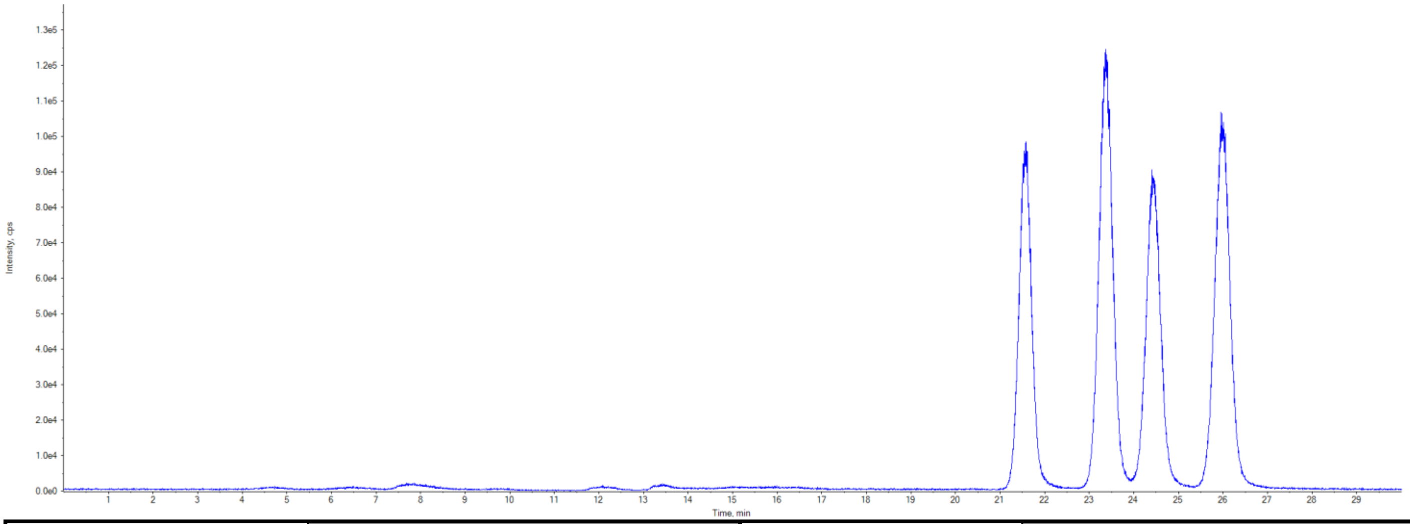


| | | | |
|---|---|---|---|
| **Sample Name** | 0.004-10d-1 | **Injection Vial** | 52 |
| **Data File** | 20220820 BHZ-YP-20du.wiff | **Injection Volume** | 10 |
| **Acquisition Date** | 8/21/2022 12:28:49 PM | **Algorithm Used** | MQL |
| **Acquisition Method** | BHZ method.dam | **Sample Type** | Unknown |
| **Instrument Name** | AB SCIEX Triple Quad 4500 | **Result Table** | 20-0.004-10d-1.rdb |
| **Sample ID** | *No data for Sample ID* | **Dilution Factor** | 1.00 |
| **Sample Comment** | *No data for Sample Comment* | **Weight to Volume** | 0.00 |

Approved By (Date and Initials):________________________________________________.

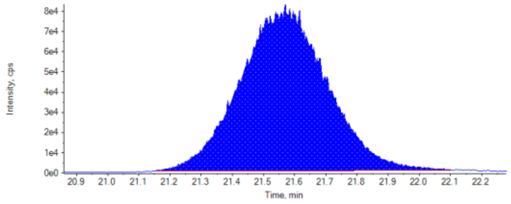


| | |
|---|---|
| **Compound Name:** | F1 **(**342.000/159.100 Da**)** |
| Expected RT: | 21.6 |
| Actual RT: | 21.6 |
| Equation: | At least 2 points are required to calculate regression. |
| Area Counts: | 1.61e+006 |
| ISTD Area Counts: | N/A |
| Amount: | 0.00 (ng/mL) |

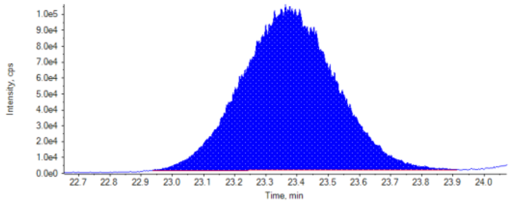


| **Compound Name:** | F2 **(**342.000/159.100 Da**)** |
|---|---|
| Expected RT: | 23.4 |
| Actual RT: | 23.4 |
| Equation: | At least 2 points are required to calculate regression. |
| Area Counts: | 2.19e+006 |
| ISTD Area Counts: | N/A |
| Amount: | 0.00 (ng/mL) |

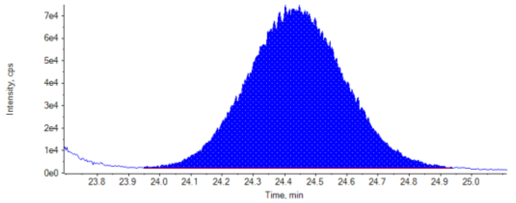


| **Compound Name:** | F3 **(**342.000/159.100 Da**)** |
|---|---|
| Expected RT: | 24.4 |
| Actual RT: | 24.4 |
| Equation: | At least 2 points are required to calculate regression. |
| Area Counts: | 1.60e+006 |
| ISTD Area Counts: | N/A |
| Amount: | 0.00 (ng/mL) |

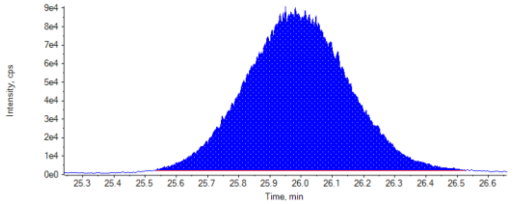


| **Compound Name:** | F4 **(**342.000/159.100 Da**)** |
|---|---|
| Expected RT: | 25.9 |
| Actual RT: | 25.9 |
| Equation: | At least 2 points are required to calculate regression. |
| Area Counts: | 2.08e+006 |
| ISTD Area Counts: | N/A |
| Amount: | 0.00 (ng/mL) |

Figure S6 The LC chromatogram of plums treated with 0.004 a.i. g/L propiconazole at 10 d (20℃)

Figure Caption
Figure_7_SuppInfo.

| | | | |
|---|---|---|---|
| **Data File** | 20220820 BHZ-YP-20du.wiff | **Result Table** | 20-0.004-15d-3.rdb |
| **Acquisition Date** | 8/21/2022 11:57:21 AM | **Algorithm Used** | MQL |
| **Acquisition Method** | BHZ method.dam | **Instrument Name** | AB SCIEX Triple Quad 4500 |
| **Project** | DPY | **Processing Method** | *No data for Processing Method* |

**Sample Name:** 0.004-15d-3 **Vial #:** 51

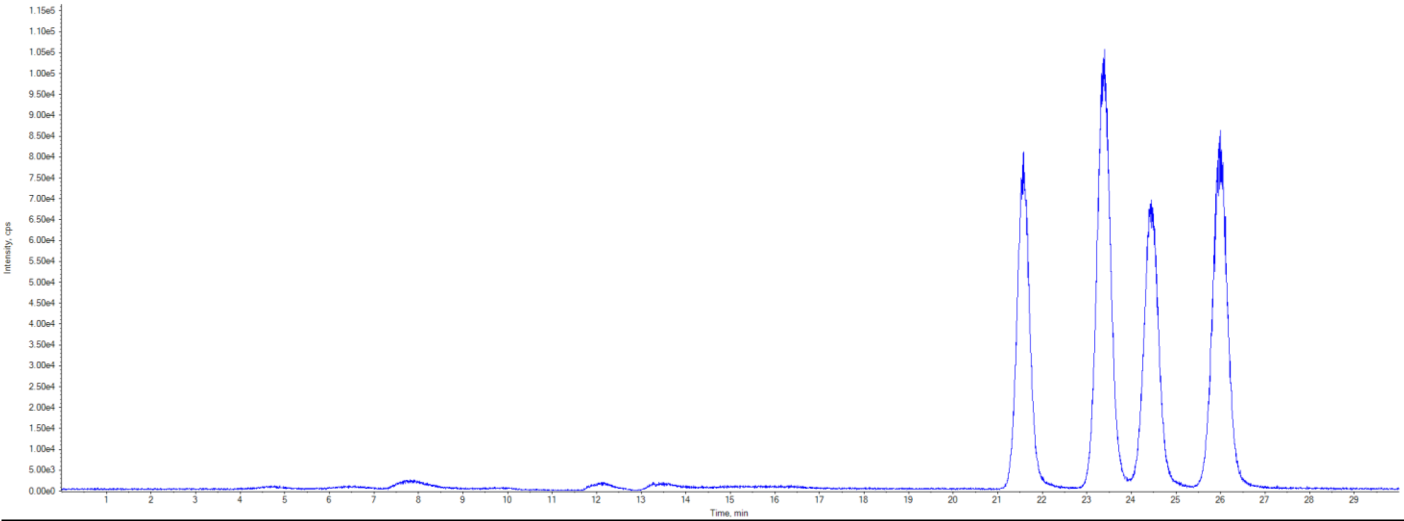


| | | | |
|---|---|---|---|
| **Sample Name** | 0.004-15d-3 | **Injection Vial** | 51 |
| **Data File** | 20220820 BHZ-YP-20du.wiff | **Injection Volume** | 10 |
| **Acquisition Date** | 8/21/2022 11:57:21 AM | **Algorithm Used** | MQL |
| **Acquisition Method** | BHZ method.dam | **Sample Type** | Unknown |
| **Instrument Name** | AB SCIEX Triple Quad 4500 | **Result Table** | 20-0.004-15d-3.rdb |
| **Sample ID** | *No data for Sample ID* | **Dilution Factor** | 1.00 |
| **Sample Comment** | *No data for Sample Comment* | **Weight to Volume** | 0.00 |

Approved By (Date and Initials):______________________________________________.

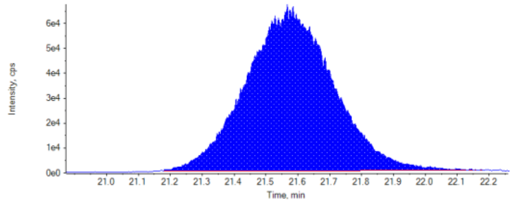


| | |
|---|---|
| **Compound Name:** | F1 **(**342.000/159.100 Da**)** |
| Expected RT: | 21.6 |
| Actual RT: | 21.6 |
| Equation: | At least 2 points are required to calculate regression. |
| Area Counts: | 1.28e+006 |
| ISTD Area Counts: | N/A |
| Amount: | 0.00 (ng/mL) |

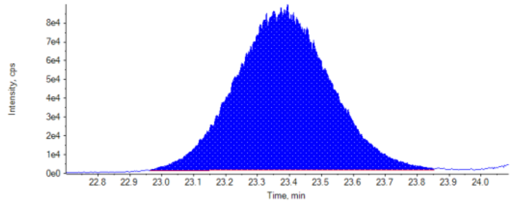


| Compound Name: | F2 (342.000/159.100 Da) |
|---|---|
| Expected RT: | 23.4 |
| Actual RT: | 23.4 |
| Equation: | At least 2 points are required to calculate regression. |
| Area Counts: | 1.81e+006 |
| ISTD Area Counts: | N/A |
| Amount: | 0.00 (ng/mL) |

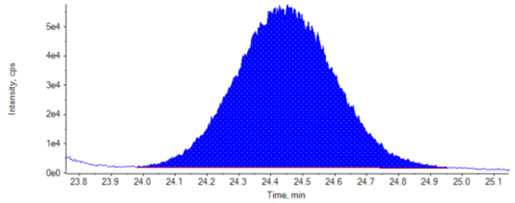


| Compound Name: | F3 (342.000/159.100 Da) |
|---|---|
| Expected RT: | 24.5 |
| Actual RT: | 24.5 |
| Equation: | At least 2 points are required to calculate regression. |
| Area Counts: | 1.24e+006 |
| ISTD Area Counts: | N/A |
| Amount: | 0.00 (ng/mL) |

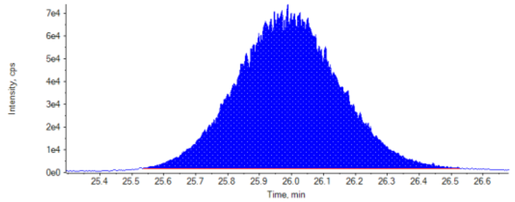


| Compound Name: | F4 (342.000/159.100 Da) |
|---|---|
| Expected RT: | 26.0 |
| Actual RT: | 26.0 |
| Equation: | At least 2 points are required to calculate regression. |
| Area Counts: | 1.61e+006 |
| ISTD Area Counts: | N/A |
| Amount: | 0.00 (ng/mL) |

Figure S7 The LC chromatogram of plums treated with 0.004 a.i. g/L propiconazole at 15 d (20℃)

Figure Caption
Figure_8_SuppInfo.

| | | | |
|---|---|---|---|
| **Data File** | 20220820 BHZ-YP-20du.wiff | **Result Table** | 20-0.004-20d-2.rdb |
| **Acquisition Date** | 8/21/2022 10:23:01 AM | **Algorithm Used** | MQL |
| **Acquisition Method** | BHZ method.dam | **Instrument Name** | AB SCIEX Triple Quad 4500 |
| **Project** | DPY | **Processing Method** | *No data for Processing Method* |

**Sample Name:** 0.004-20d-3 **Vial #:** 48

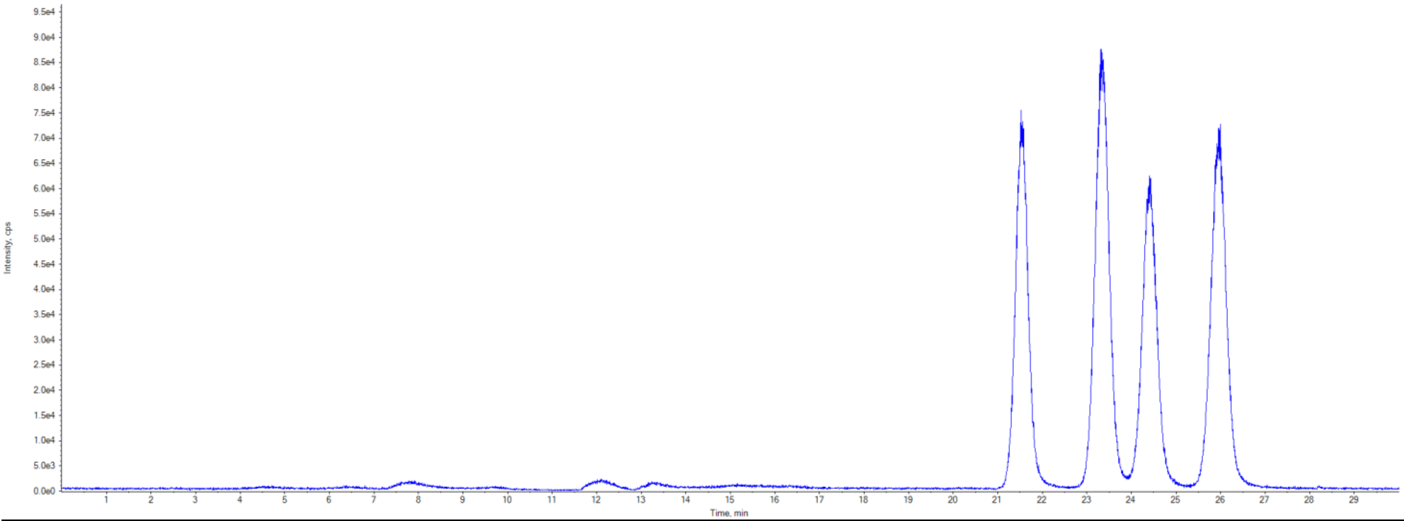

| | | | |
|---|---|---|---|
| **Sample Name** | 0.004-20d-3 | **Injection Vial** | 48 |
| **Data File** | 20220820 BHZ-YP-20du.wiff | **Injection Volume** | 10 |
| **Acquisition Date** | 8/21/2022 10:23:01 AM | **Algorithm Used** | MQL |
| **Acquisition Method** | BHZ method.dam | **Sample Type** | Unknown |
| **Instrument Name** | AB SCIEX Triple Quad 4500 | **Result Table** | 20-0.004-20d-2.rdb |
| **Sample ID** | *No data for Sample ID* | **Dilution Factor** | 1.00 |
| **Sample Comment** | *No data for Sample Comment* | **Weight to Volume** | 0.00 |

Approved By (Date and Initials):________________________________________.

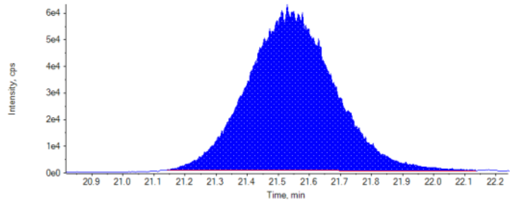

| | |
|---|---|
| **Compound Name:** | F1 **(**342.000/159.100 Da**)** |
| Expected RT: | 21.5 |
| Actual RT: | 21.5 |
| Equation: | At least 2 points are required to calculate regression. |
| Area Counts: | 1.12e+006 |
| ISTD Area Counts: | N/A |
| Amount: | 0.00 (ng/mL) |

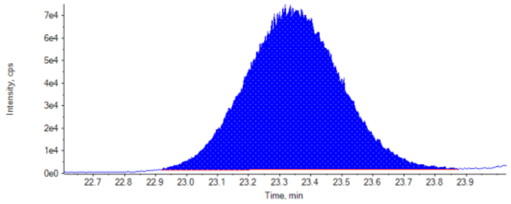


| **Compound Name:** | F2 **(**342.000/159.100 Da**)** |
|---|---|
| Expected RT: | 23.3 |
| Actual RT: | 23.3 |
| Equation: | At least 2 points are required to calculate regression. |
| Area Counts: | 1.48e+006 |
| ISTD Area Counts: | N/A |
| Amount: | 0.00 (ng/mL) |

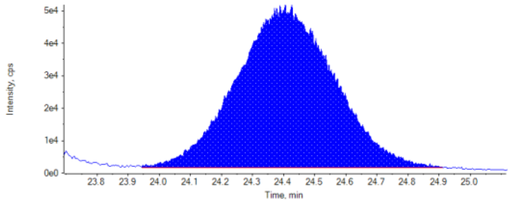


| **Compound Name:** | F3 **(**342.000/159.100 Da**)** |
|---|---|
| Expected RT: | 24.4 |
| Actual RT: | 24.4 |
| Equation: | At least 2 points are required to calculate regression. |
| Area Counts: | 1.03e+006 |
| ISTD Area Counts: | N/A |
| Amount: | 0.00 (ng/mL) |

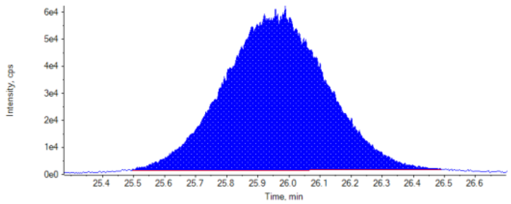


| **Compound Name:** | F4 **(**342.000/159.100 Da**)** |
|---|---|
| Expected RT: | 26.0 |
| Actual RT: | 26.0 |
| Equation: | At least 2 points are required to calculate regression. |
| Area Counts: | 1.30e+006 |
| ISTD Area Counts: | N/A |
| Amount: | 0.00 (ng/mL) |

Figure S8 The LC chromatogram of plums treated with 0.004 a.i. g/L propiconazole at 20 d (20℃)

Created with No data for MQ Created With
Printed:No data for MQ Report Generation Date

Figure Caption
Figure_9_SuppInfo.

| | | | |
|---|---|---|---|
| **Data File** | 20220820 BHZ-YP-20du.wiff | **Result Table** | 20-0.004-25d-2.rdb |
| **Acquisition Date** | 8/21/2022 8:17:11 AM | **Algorithm Used** | IntelliQuan |
| **Acquisition Method** | BHZ  method.dam | **Instrument Name** | AB SCIEX Triple Quad 4500 |
| **Project** | DPY | **Processing Method** | No data for Processing Method |

**Sample Name:** 0.004-25d-2 **Vial #:** 44

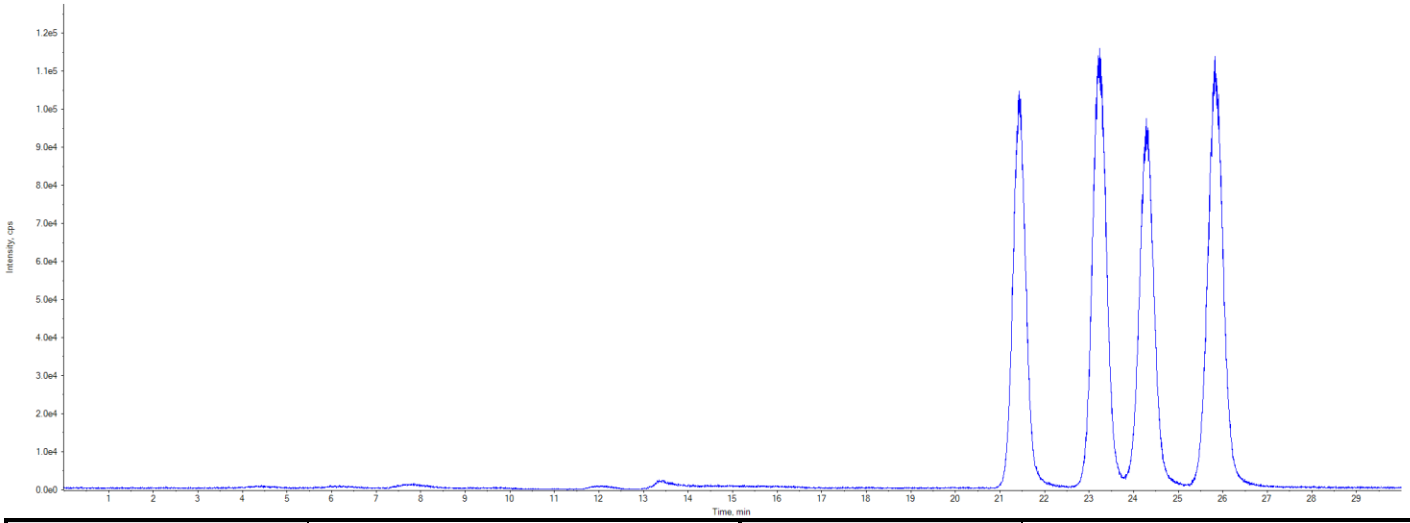


| | | | |
|---|---|---|---|
| **Sample Name** | 0.004-25d-2 | **Injection Vial** | 44 |
| **Data File** | 20220820 BHZ-YP-20du.wiff | **Injection Volume** | 10 |
| **Acquisition Date** | 8/21/2022 8:17:11 AM | **Algorithm Used** | IntelliQuan |
| **Acquisition Method** | BHZ  method.dam | **Sample Type** | Unknown |
| **Instrument Name** | AB SCIEX Triple Quad 4500 | **Result Table** | 20-0.004-25d-2.rdb |
| **Sample ID** | No data for Sample ID | **Dilution Factor** | 1.00 |
| **Sample Comment** | No data for Sample Comment | **Weight to Volume** | 0.00 |

Approved By (Date and Initials):_______________________________________________.

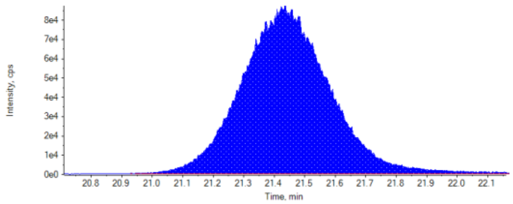


| | |
|---|---|
| **Compound Name:** | F1 **(**342.000/159.100 Da**)** |
| Expected RT: | 21.4 |
| Actual RT: | 21.4 |
| Equation: | At least 2 points are required to calculate regression. |
| Area Counts: | 1.73e+006 |
| ISTD Area Counts: | N/A |
| Amount: | 0.00 (ng/mL) |

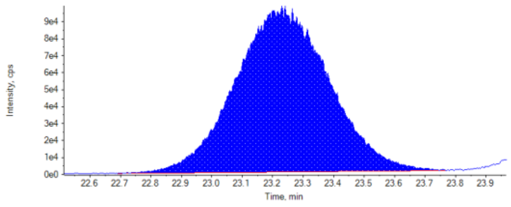


| **Compound Name:** | F2 **(**342.000/159.100 Da**)** |
|---|---|
| Expected RT: | 23.2 |
| Actual RT: | 23.2 |
| Equation: | At least 2 points are required to calculate regression. |
| Area Counts: | 2.07e+006 |
| ISTD Area Counts: | N/A |
| Amount: | 0.00 (ng/mL) |

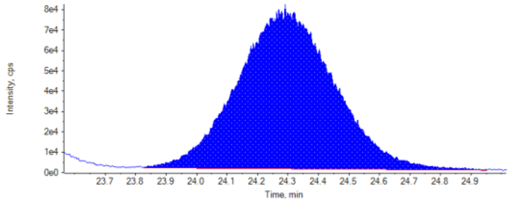


| **Compound Name:** | F3 **(**342.000/159.100 Da**)** |
|---|---|
| Expected RT: | 24.3 |
| Actual RT: | 24.3 |
| Equation: | At least 2 points are required to calculate regression. |
| Area Counts: | 1.72e+006 |
| ISTD Area Counts: | N/A |
| Amount: | 0.00 (ng/mL) |

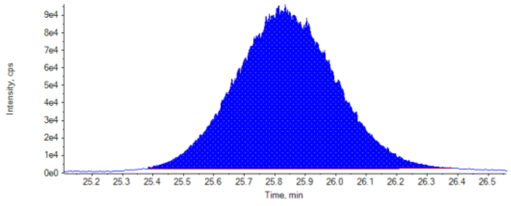


| **Compound Name:** | F4 **(**342.000/159.100 Da**)** |
|---|---|
| Expected RT: | 25.8 |
| Actual RT: | 25.8 |
| Equation: | At least 2 points are required to calculate regression. |
| Area Counts: | 2.24e+006 |
| ISTD Area Counts: | N/A |
| Amount: | 0.00 (ng/mL) |

Figure S9 plums treated with 0.004 a.i. g/L propiconazole were sampled on 25d when stored at 20℃

Created with No data for MQ Created With
Printed:No data for MQ Report Generation Date

Figure Caption
Figure_10_SuppInfo.

| Data File | 20220820 BHZ-YP-20du.wiff | Result Table | 20-0.004-30d-2.rdb |
|---|---|---|---|
| Acquisition Date | 8/21/2022 6:42:48 AM | Algorithm Used | MQL |
| Acquisition Method | BHZ  method.dam | Instrument Name | AB SCIEX Triple Quad 4500 |
| Project | DPY | Processing Method | No data for Processing Method |

**Sample Name:** 0.004-30d-2 **Vial #:** 41

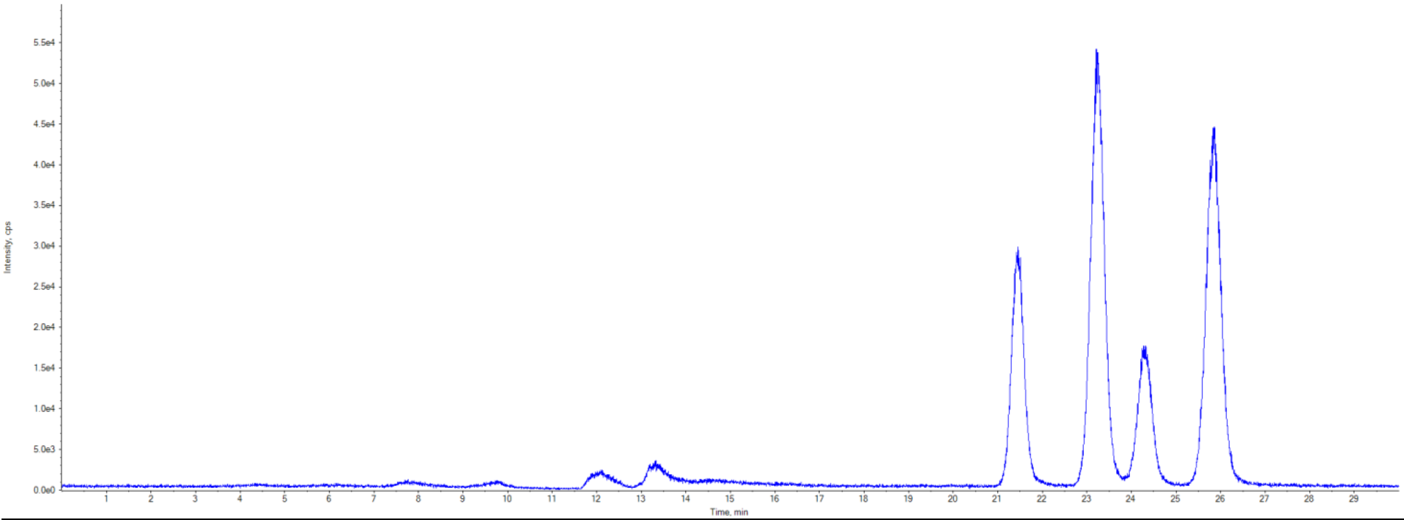


| Sample Name | 0.004-30d-2 | Injection Vial | 41 |
|---|---|---|---|
| Data File | 20220820 BHZ-YP-20du.wiff | Injection Volume | 10 |
| Acquisition Date | 8/21/2022 6:42:48 AM | Algorithm Used | MQL |
| Acquisition Method | BHZ  method.dam | Sample Type | Unknown |
| Instrument Name | AB SCIEX Triple Quad 4500 | Result Table | 20-0.004-30d-2.rdb |
| Sample ID | No data for Sample ID | Dilution Factor | 1.00 |
| Sample Comment | No data for Sample Comment | Weight to Volume | 0.00 |

Approved By (Date and Initials):________________________________________________.

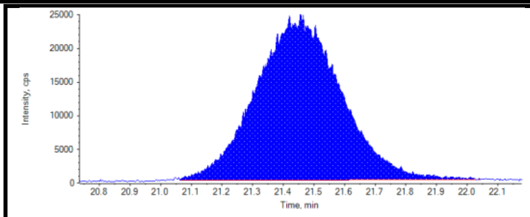


| **Compound Name:** | F1 **(**342.000/159.100 Da**)** |
|---|---|
| Expected RT: | 21.5 |
| Actual RT: | 21.5 |
| Equation: | At least 2 points are required to calculate regression. |
| Area Counts: | 4.80e+005 |
| ISTD Area Counts: | N/A |
| Amount: | 0.00 (ng/mL) |

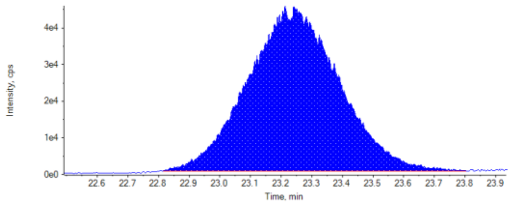


| Compound Name: | F2 (342.000/159.100 Da) |
|---|---|
| Expected RT: | 23.2 |
| Actual RT: | 23.2 |
| Equation: | At least 2 points are required to calculate regression. |
| Area Counts: | 9.44e+005 |
| ISTD Area Counts: | N/A |
| Amount: | 0.00 (ng/mL) |

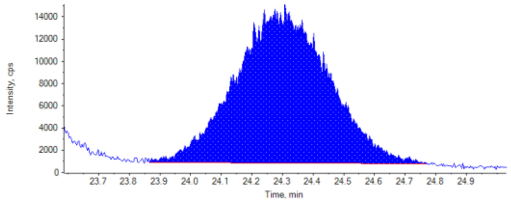


| Compound Name: | F3 (342.000/159.100 Da) |
|---|---|
| Expected RT: | 24.3 |
| Actual RT: | 24.3 |
| Equation: | At least 2 points are required to calculate regression. |
| Area Counts: | 3.03e+005 |
| ISTD Area Counts: | N/A |
| Amount: | 0.00 (ng/mL) |

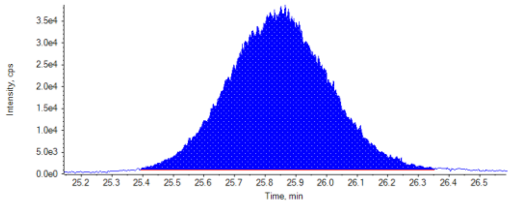


| Compound Name: | F4 (342.000/159.100 Da) |
|---|---|
| Expected RT: | 25.9 |
| Actual RT: | 25.9 |
| Equation: | At least 2 points are required to calculate regression. |
| Area Counts: | 8.79e+005 |
| ISTD Area Counts: | N/A |
| Amount: | 0.00 (ng/mL) |

Figure S10 The LC chromatogram of plums treated with 0.004 a.i. g/L propiconazole at 30 d (20°C)

Figure Caption
Figure_11_SuppInfo.

Created with No data for MQ Created With
Printed:No data for MQ Report Generation Date

| | | | |
|---|---|---|---|
| **Data File** | 20220820 BHZ-YP-20du.wiff | **Result Table** | 20-0.02-5d-1.rdb |
| **Acquisition Date** | 8/21/2022 1:28:15 AM | **Algorithm Used** | MQL |
| **Acquisition Method** | BHZ method.dam | **Instrument Name** | AB SCIEX Triple Quad 4500 |
| **Project** | DPY | **Processing Method** | No data for Processing Method |

**Sample Name:** 0.02-5d-1 **Vial #:** 31

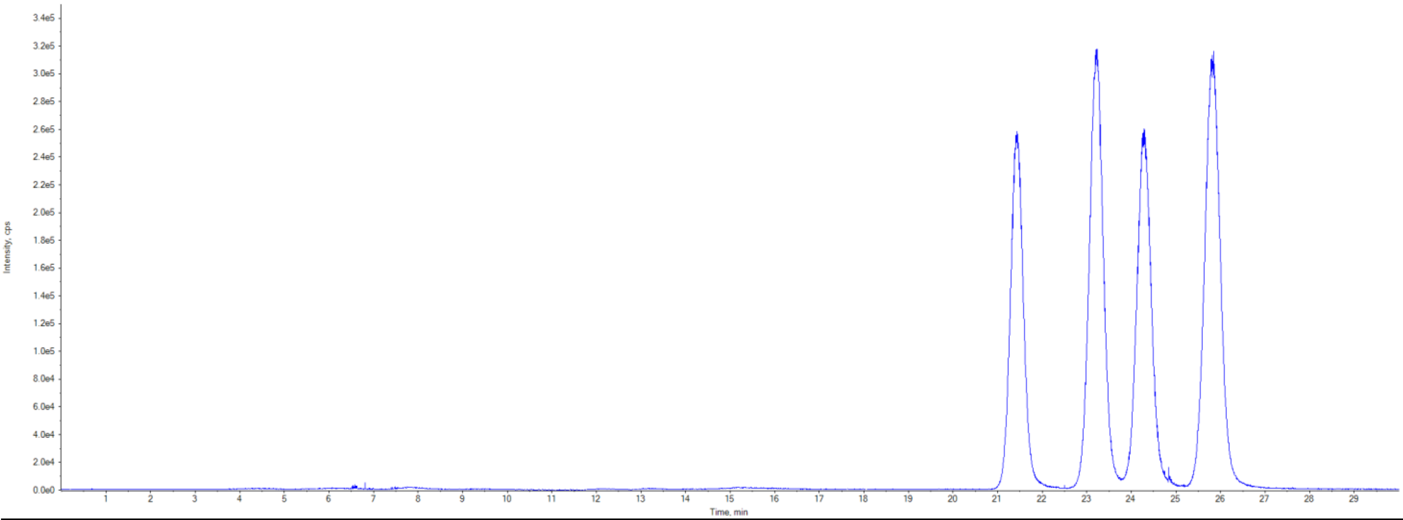


| | | | |
|---|---|---|---|
| **Sample Name** | 0.02-5d-1 | **Injection Vial** | 31 |
| **Data File** | 20220820 BHZ-YP-20du.wiff | **Injection Volume** | 10 |
| **Acquisition Date** | 8/21/2022 1:28:15 AM | **Algorithm Used** | MQL |
| **Acquisition Method** | BHZ method.dam | **Sample Type** | Unknown |
| **Instrument Name** | AB SCIEX Triple Quad 4500 | **Result Table** | 20-0.02-5d-1.rdb |
| **Sample ID** | No data for Sample ID | **Dilution Factor** | 1.00 |
| **Sample Comment** | No data for Sample Comment | **Weight to Volume** | 0.00 |

Approved By (Date and Initials):________________________________________________.

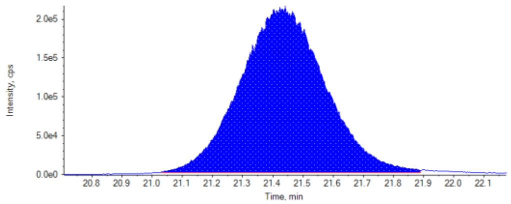


| | |
|---|---|
| **Compound Name:** | F1 **(**342.000/159.100 Da**)** |
| Expected RT: | 21.4 |
| Actual RT: | 21.4 |
| Equation: | At least 2 points are required to calculate regression. |
| Area Counts: | 4.33e+006 |
| ISTD Area Counts: | N/A |
| Amount: | 0.00 (ng/mL) |

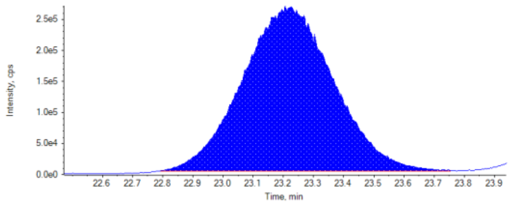


| **Compound Name:** | F2 **(**342.000/159.100 Da**)** |
|---|---|
| Expected RT: | 23.2 |
| Actual RT: | 23.2 |
| Equation: | At least 2 points are required to calculate regression. |
| Area Counts: | 5.70e+006 |
| ISTD Area Counts: | N/A |
| Amount: | 0.00 (ng/mL) |

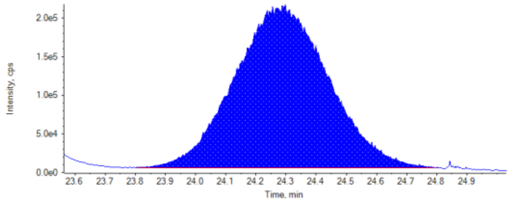


| **Compound Name:** | F3 **(**342.000/159.100 Da**)** |
|---|---|
| Expected RT: | 24.3 |
| Actual RT: | 24.3 |
| Equation: | At least 2 points are required to calculate regression. |
| Area Counts: | 4.58e+006 |
| ISTD Area Counts: | N/A |
| Amount: | 0.00 (ng/mL) |

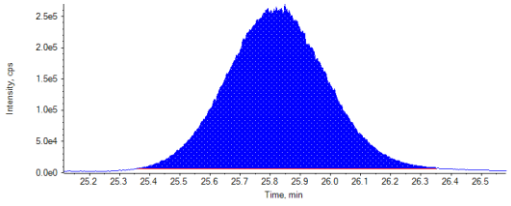


| **Compound Name:** | F4 **(**342.000/159.100 Da**)** |
|---|---|
| Expected RT: | 25.8 |
| Actual RT: | 25.8 |
| Equation: | At least 2 points are required to calculate regression. |
| Area Counts: | 6.39e+006 |
| ISTD Area Counts: | N/A |
| Amount: | 0.00 (ng/mL) |

Figure S11 The LC chromatogram of plums treated with 0.020 a.i. g/L propiconazole at 5 d (20°C)

Created with No data for MQ Created With
Printed:No data for MQ Report Generation Date

Figure Caption
Figure_12_SuppInfo.

| | | | |
|---|---|---|---|
| **Data File** | 20220820 BHZ-YP-20du.wiff | **Result Table** | 20-0.02-10d-1.rdb |
| **Acquisition Date** | 8/20/2022 11:53:52 PM | **Algorithm Used** | MQL |
| **Acquisition Method** | BHZ method.dam | **Instrument Name** | AB SCIEX Triple Quad 4500 |
| **Project** | DPY | **Processing Method** | No data for Processing Method |

**Sample Name:** 0.02-10d-1 **Vial #:** 28

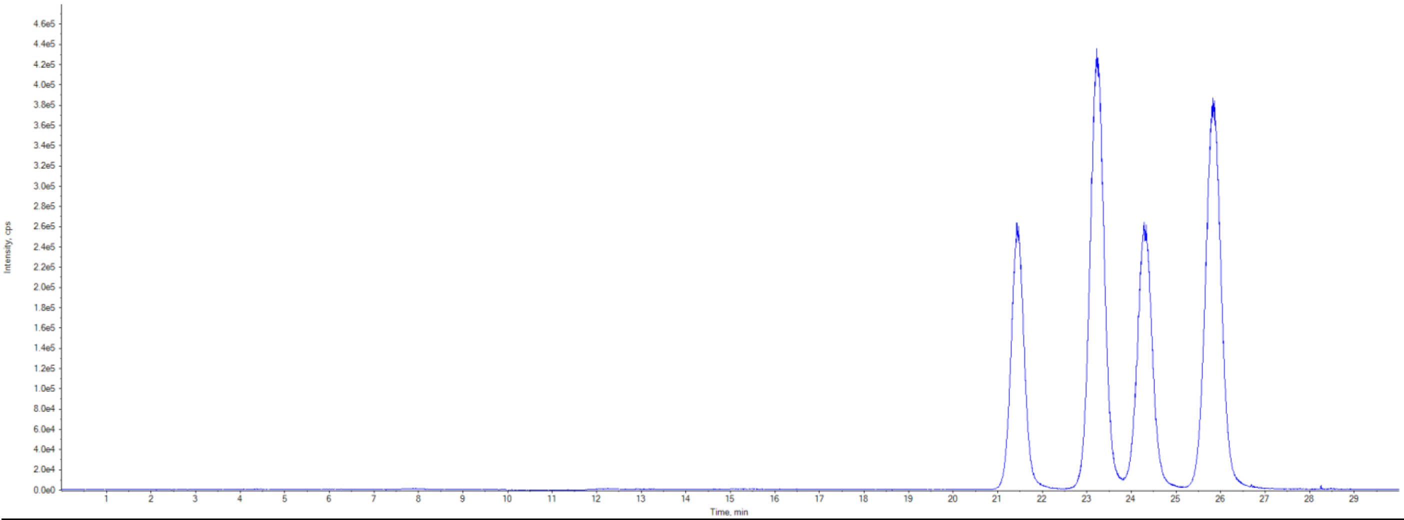


| | | | |
|---|---|---|---|
| **Sample Name** | 0.02-10d-1 | **Injection Vial** | 28 |
| **Data File** | 20220820 BHZ-YP-20du.wiff | **Injection Volume** | 10 |
| **Acquisition Date** | 8/20/2022 11:53:52 PM | **Algorithm Used** | MQL |
| **Acquisition Method** | BHZ method.dam | **Sample Type** | Unknown |
| **Instrument Name** | AB SCIEX Triple Quad 4500 | **Result Table** | 20-0.02-10d-1.rdb |
| **Sample ID** | No data for Sample ID | **Dilution Factor** | 1.00 |
| **Sample Comment** | No data for Sample Comment | **Weight to Volume** | 0.00 |

Approved By (Date and Initials):___________________________________________________.

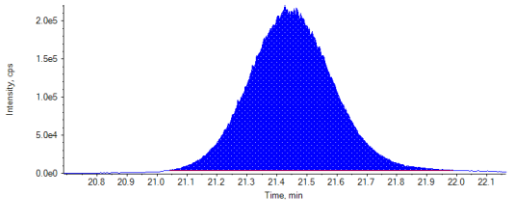


| | |
|---|---|
| **Compound Name:** | F1 **(**342.000/159.100 Da**)** |
| Expected RT: | 21.4 |
| Actual RT: | 21.4 |
| Equation: | At least 2 points are required to calculate regression. |
| Area Counts: | 4.42e+006 |
| ISTD Area Counts: | N/A |
| Amount: | 0.00 (ng/mL) |

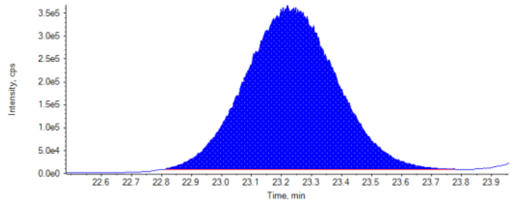


| **Compound Name:** | F2 **(**342.000/159.100 Da**)** |
|---|---|
| Expected RT: | 23.2 |
| Actual RT: | 23.2 |
| Equation: | At least 2 points are required to calculate regression. |
| Area Counts: | 7.83e+006 |
| ISTD Area Counts: | N/A |
| Amount: | 0.00 (ng/mL) |

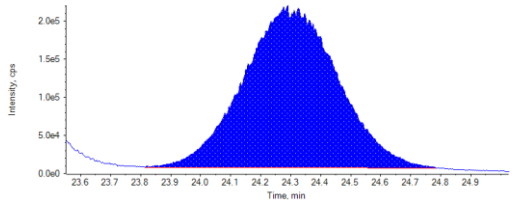


| **Compound Name:** | F3 **(**342.000/159.100 Da**)** |
|---|---|
| Expected RT: | 24.3 |
| Actual RT: | 24.3 |
| Equation: | At least 2 points are required to calculate regression. |
| Area Counts: | 4.74e+006 |
| ISTD Area Counts: | N/A |
| Amount: | 0.00 (ng/mL) |

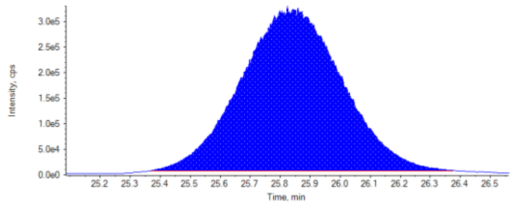


| **Compound Name:** | F4 **(**342.000/159.100 Da**)** |
|---|---|
| Expected RT: | 25.8 |
| Actual RT: | 25.8 |
| Equation: | At least 2 points are required to calculate regression. |
| Area Counts: | 7.83e+006 |
| ISTD Area Counts: | N/A |
| Amount: | 0.00 (ng/mL) |

Figure S12 The LC chromatogram of plums treated with 0.020 a.i. g/L propiconazole at 10 d (20℃)

Created with No data for MQ Created With
Printed:No data for MQ Report Generation Date

Figure Caption
Figure_13_SuppInfo.

| | | | |
|---|---|---|---|
| **Data File** | 20220820 BHZ-YP-20du.wiff | **Result Table** | 20-0.02-15d-3.rdb |
| **Acquisition Date** | 8/20/2022 11:22:24 PM | **Algorithm Used** | MQL |
| **Acquisition Method** | BHZ method.dam | **Instrument Name** | AB SCIEX Triple Quad 4500 |
| **Project** | DPY | **Processing Method** | No data for Processing Method |

**Sample Name:** 0.02-15d-3 **Vial #:** 27

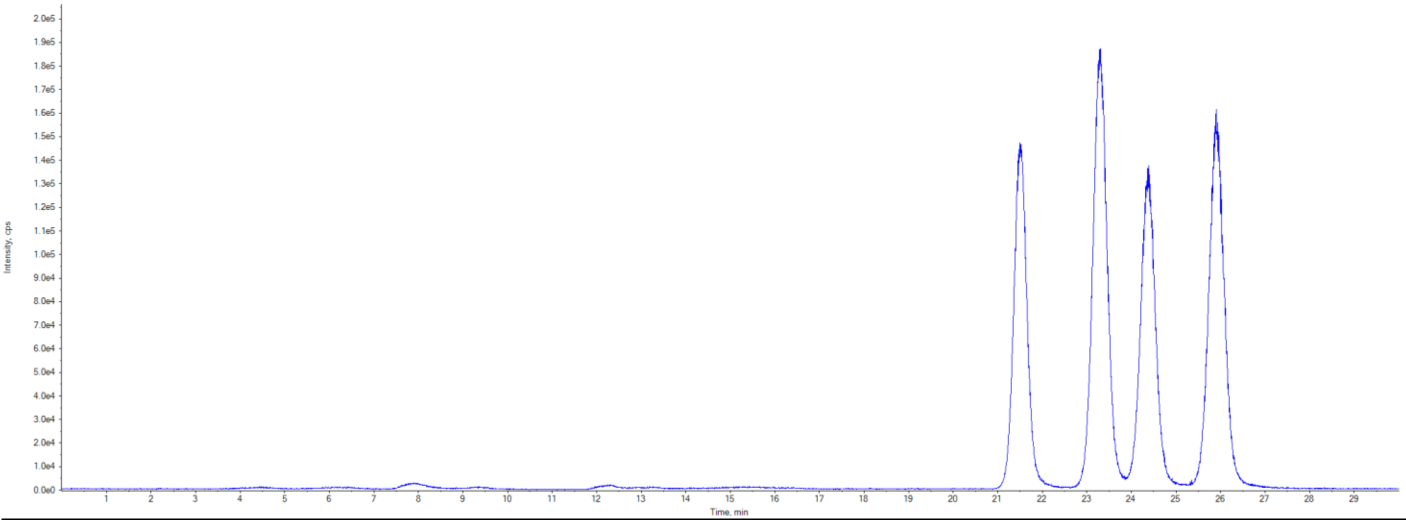


| | | | |
|---|---|---|---|
| **Sample Name** | 0.02-15d-3 | **Injection Vial** | 27 |
| **Data File** | 20220820 BHZ-YP-20du.wiff | **Injection Volume** | 10 |
| **Acquisition Date** | 8/20/2022 11:22:24 PM | **Algorithm Used** | MQL |
| **Acquisition Method** | BHZ method.dam | **Sample Type** | Unknown |
| **Instrument Name** | AB SCIEX Triple Quad 4500 | **Result Table** | 20-0.02-15d-3.rdb |
| **Sample ID** | No data for Sample ID | **Dilution Factor** | 1.00 |
| **Sample Comment** | No data for Sample Comment | **Weight to Volume** | 0.00 |

Approved By (Date and Initials):______________________________________________.

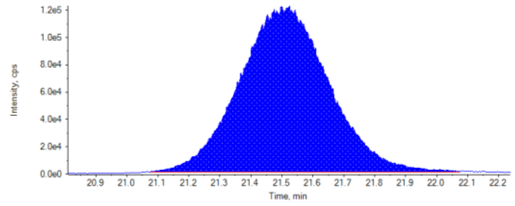


| | |
|---|---|
| **Compound Name:** | F1 **(**342.000/159.100 Da**)** |
| Expected RT: | 21.5 |
| Actual RT: | 21.5 |
| Equation: | At least 2 points are required to calculate regression. |
| Area Counts: | 2.52e+006 |
| ISTD Area Counts: | N/A |
| Amount: | 0.00 (ng/mL) |

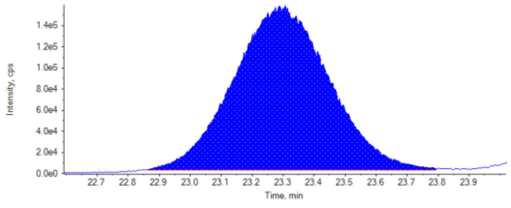


| **Compound Name:** | F2 **(**342.000/159.100 Da**)** |
|---|---|
| Expected RT: | 23.3 |
| Actual RT: | 23.3 |
| Equation: | At least 2 points are required to calculate regression. |
| Area Counts: | 3.41e+006 |
| ISTD Area Counts: | N/A |
| Amount: | 0.00 (ng/mL) |

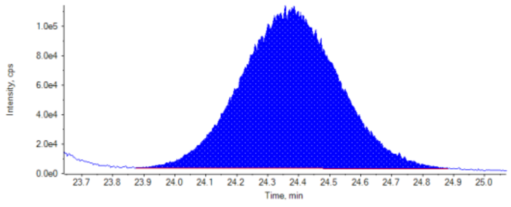


| **Compound Name:** | F3 **(**342.000/159.100 Da**)** |
|---|---|
| Expected RT: | 24.4 |
| Actual RT: | 24.4 |
| Equation: | At least 2 points are required to calculate regression. |
| Area Counts: | 2.44e+006 |
| ISTD Area Counts: | N/A |
| Amount: | 0.00 (ng/mL) |

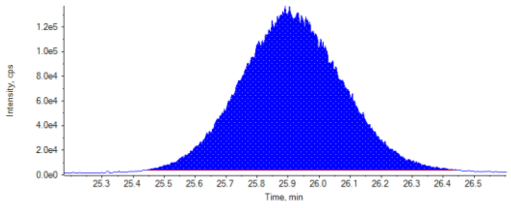


| **Compound Name:** | F4 **(**342.000/159.100 Da**)** |
|---|---|
| Expected RT: | 25.9 |
| Actual RT: | 25.9 |
| Equation: | At least 2 points are required to calculate regression. |
| Area Counts: | 3.14e+006 |
| ISTD Area Counts: | N/A |
| Amount: | 0.00 (ng/mL) |

Figure S13 The LC chromatogram of plums treated with 0.020 a.i. g/L propiconazole at 15 d (20°C)

Created with No data for MQ Created With
Printed:No data for MQ Report Generation Date

Figure Caption
Figure_14_SuppInfo.

| | | | |
|---|---|---|---|
| **Data File** | 20220820 BHZ-YP-20du.wiff | **Result Table** | 20-0.02-20d-1.rdb |
| **Acquisition Date** | 8/20/2022 8:45:06 PM | **Algorithm Used** | MQL |
| **Acquisition Method** | BHZ  method.dam | **Instrument Name** | AB SCIEX Triple Quad 4500 |
| **Project** | DPY | **Processing Method** | No data for Processing Method |

**Sample Name:** 0.02-20d-1 **Vial #:** 22

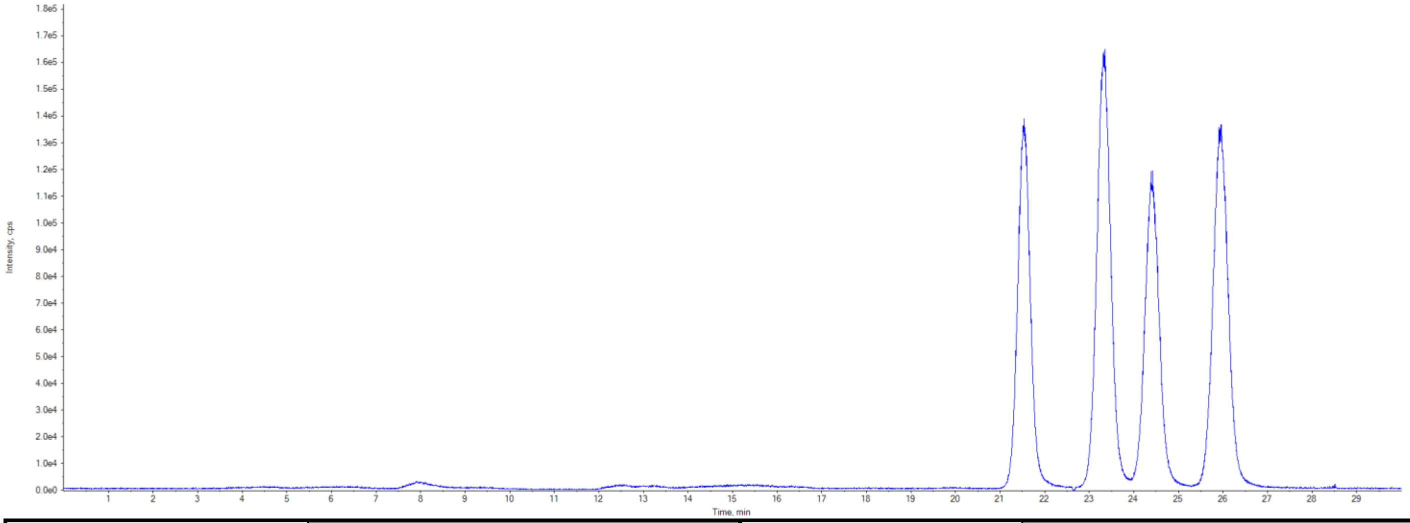


| | | | |
|---|---|---|---|
| **Sample Name** | 0.02-20d-1 | **Injection Vial** | 22 |
| **Data File** | 20220820 BHZ-YP-20du.wiff | **Injection Volume** | 10 |
| **Acquisition Date** | 8/20/2022 8:45:06 PM | **Algorithm Used** | MQL |
| **Acquisition Method** | BHZ  method.dam | **Sample Type** | Unknown |
| **Instrument Name** | AB SCIEX Triple Quad 4500 | **Result Table** | 20-0.02-20d-1.rdb |
| **Sample ID** | No data for Sample ID | **Dilution Factor** | 1.00 |
| **Sample Comment** | No data for Sample Comment | **Weight to Volume** | 0.00 |

Approved By (Date and Initials):________________________________________________________.

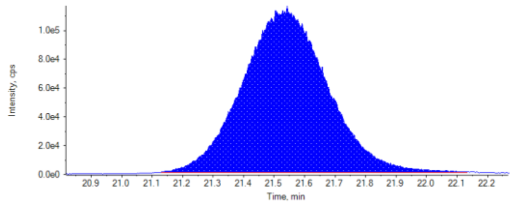


| **Compound Name:** | F1 **(**342.000/159.100 Da**)** |
|---|---|
| Expected RT: | 21.5 |
| Actual RT: | 21.5 |
| Equation: | At least 2 points are required to calculate regression. |
| Area Counts: | 2.25e+006 |
| ISTD Area Counts: | N/A |
| Amount: | 0.00 (ng/mL) |

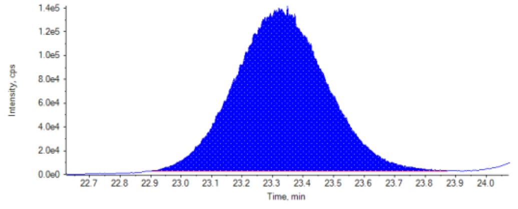


| **Compound Name:** | F2 **(**342.000/159.100 Da**)** |
|---|---|
| Expected RT: | 23.3 |
| Actual RT: | 23.3 |
| Equation: | At least 2 points are required to calculate regression. |
| Area Counts: | 2.93e+006 |
| ISTD Area Counts: | N/A |
| Amount: | 0.00 (ng/mL) |

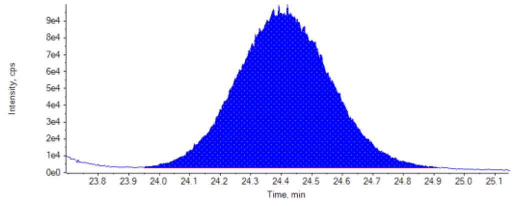


| **Compound Name:** | F3 **(**342.000/159.100 Da**)** |
|---|---|
| Expected RT: | 24.4 |
| Actual RT: | 24.4 |
| Equation: | At least 2 points are required to calculate regression. |
| Area Counts: | 2.08e+006 |
| ISTD Area Counts: | N/A |
| Amount: | 0.00 (ng/mL) |

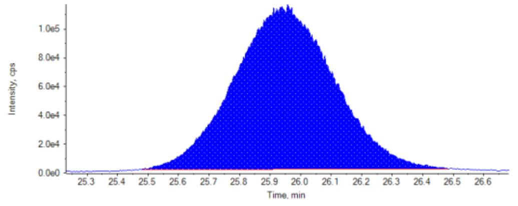


| **Compound Name:** | F4 **(**342.000/159.100 Da**)** |
|---|---|
| Expected RT: | 26.0 |
| Actual RT: | 26.0 |
| Equation: | At least 2 points are required to calculate regression. |
| Area Counts: | 2.73e+006 |
| ISTD Area Counts: | N/A |
| Amount: | 0.00 (ng/mL) |

Figure S14 The LC chromatogram of plums treated with 0.020 a.i. g/L propiconazole at 20 d (20°C)

Created with No data for MQ Created With
Printed:No data for MQ Report Generation Date

Figure Caption
Figure_15_SuppInfo.

| Data File | 20220820 BHZ-YP-20du.wiff | Result Table | 20-0.02-25d-2.rdb |
|---|---|---|---|
| Acquisition Date | 8/20/2022 7:42:11 PM | Algorithm Used | MQL |
| Acquisition Method | BHZ method.dam | Instrument Name | AB SCIEX Triple Quad 4500 |
| Project | DPY | Processing Method | No data for Processing Method |

**Sample Name:** 0.02-25d-2 **Vial #:** 20

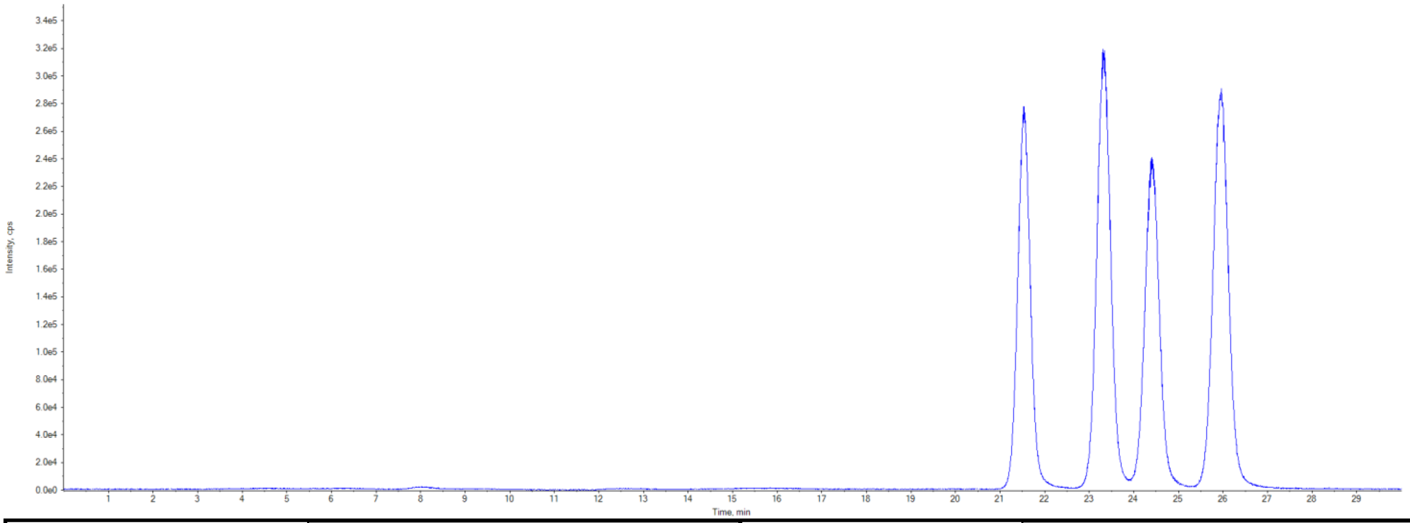


| Sample Name | 0.02-25d-2 | Injection Vial | 20 |
|---|---|---|---|
| Data File | 20220820 BHZ-YP-20du.wiff | Injection Volume | 10 |
| Acquisition Date | 8/20/2022 7:42:11 PM | Algorithm Used | MQL |
| Acquisition Method | BHZ method.dam | Sample Type | Unknown |
| Instrument Name | AB SCIEX Triple Quad 4500 | Result Table | 20-0.02-25d-2.rdb |
| Sample ID | No data for Sample ID | Dilution Factor | 1.00 |
| Sample Comment | No data for Sample Comment | Weight to Volume | 0.00 |

Approved By (Date and Initials):____________________________________________.

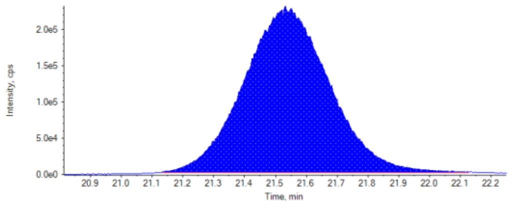


| Compound Name: | F1 (342.000/159.100 Da) |
|---|---|
| Expected RT: | 21.5 |
| Actual RT: | 21.5 |
| Equation: | At least 2 points are required to calculate regression. |
| Area Counts: | 4.54e+006 |
| ISTD Area Counts: | N/A |
| Amount: | 0.00 (ng/mL) |

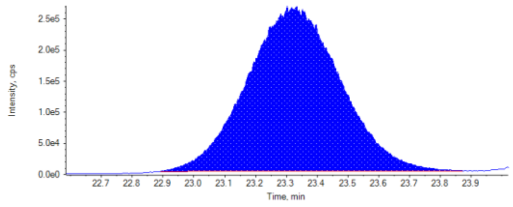


| Compound Name: | F2 (342.000/159.100 Da) |
|---|---|
| Expected RT: | 23.3 |
| Actual RT: | 23.3 |
| Equation: | At least 2 points are required to calculate regression. |
| Area Counts: | 5.68e+006 |
| ISTD Area Counts: | N/A |
| Amount: | 0.00 (ng/mL) |

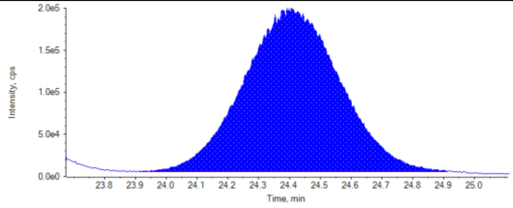


| Compound Name: | F3 (342.000/159.100 Da) |
|---|---|
| Expected RT: | 24.4 |
| Actual RT: | 24.4 |
| Equation: | At least 2 points are required to calculate regression. |
| Area Counts: | 4.33e+006 |
| ISTD Area Counts: | N/A |
| Amount: | 0.00 (ng/mL) |

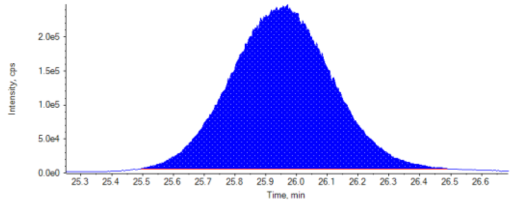


| Compound Name: | F4 (342.000/159.100 Da) |
|---|---|
| Expected RT: | 26.0 |
| Actual RT: | 26.0 |
| Equation: | At least 2 points are required to calculate regression. |
| Area Counts: | 5.87e+006 |
| ISTD Area Counts: | N/A |
| Amount: | 0.00 (ng/mL) |

Figure S15 The LC chromatogram of plums treated with 0.020 a.i. g/L propiconazole at 25 d (20℃)

Created with No data for MQ Created With
Printed:No data for MQ Report Generation Date

Figure Caption
Figure_16_SuppInfo.

| | | | |
|---|---|---|---|
| **Data File** | 20220820 BHZ-YP-20du.wiff | **Result Table** | 20-0.02-30d-2.rdb |
| **Acquisition Date** | 8/20/2022 6:07:48 PM | **Algorithm Used** | MQL |
| **Acquisition Method** | BHZ method.dam | **Instrument Name** | AB SCIEX Triple Quad 4500 |
| **Project** | DPY | **Processing Method** | No data for Processing Method |

**Sample Name:** 0.02-30d-2 **Vial #:** 17

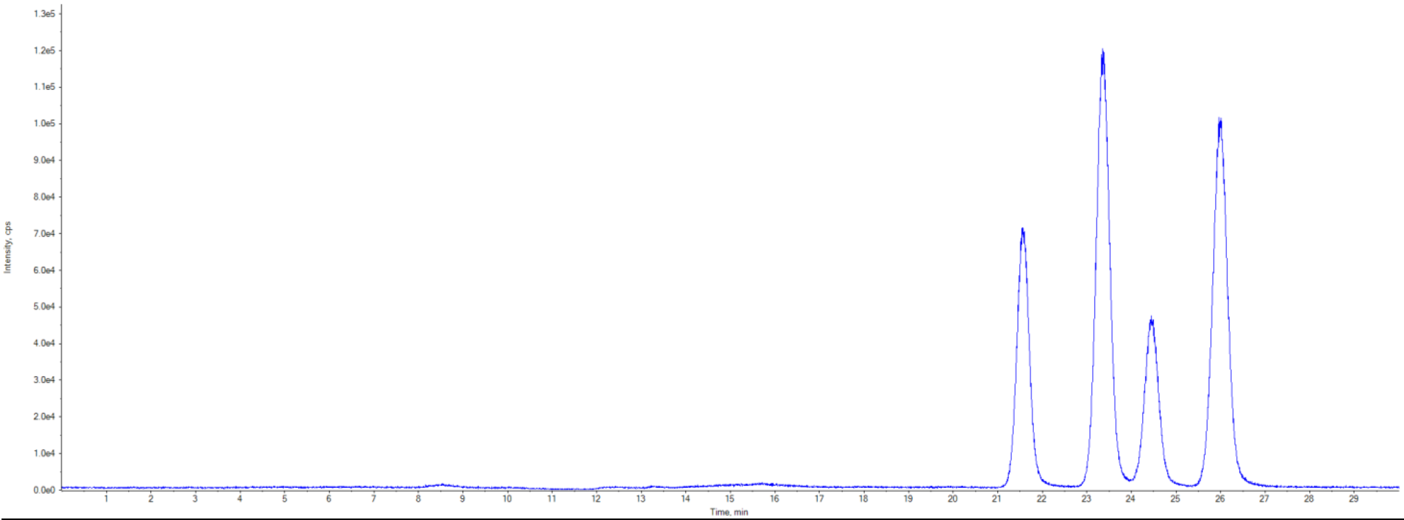


| | | | |
|---|---|---|---|
| **Sample Name** | 0.02-30d-2 | **Injection Vial** | 17 |
| **Data File** | 20220820 BHZ-YP-20du.wiff | **Injection Volume** | 10 |
| **Acquisition Date** | 8/20/2022 6:07:48 PM | **Algorithm Used** | MQL |
| **Acquisition Method** | BHZ method.dam | **Sample Type** | Unknown |
| **Instrument Name** | AB SCIEX Triple Quad 4500 | **Result Table** | 20-0.02-30d-2.rdb |
| **Sample ID** | No data for Sample ID | **Dilution Factor** | 1.00 |
| **Sample Comment** | No data for Sample Comment | **Weight to Volume** | 0.00 |

Approved By (Date and Initials):________________________________________________.

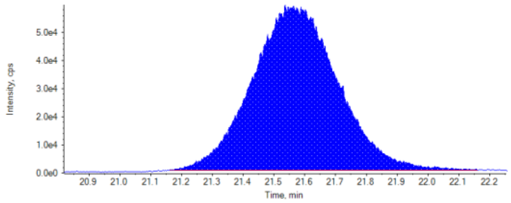


| | |
|---|---|
| **Compound Name:** | F1 **(**342.000/159.100 Da**)** |
| Expected RT: | 21.5 |
| Actual RT: | 21.5 |
| Equation: | At least 2 points are required to calculate regression. |
| Area Counts: | 1.18e+006 |
| ISTD Area Counts: | N/A |
| Amount: | 0.00 (ng/mL) |

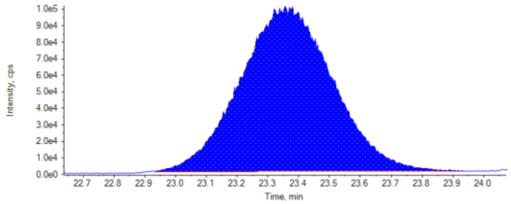


| Compound Name: | F2 (342.000/159.100 Da) |
|---|---|
| Expected RT: | 23.4 |
| Actual RT: | 23.4 |
| Equation: | At least 2 points are required to calculate regression. |
| Area Counts: | 2.12e+006 |
| ISTD Area Counts: | N/A |
| Amount: | 0.00 (ng/mL) |

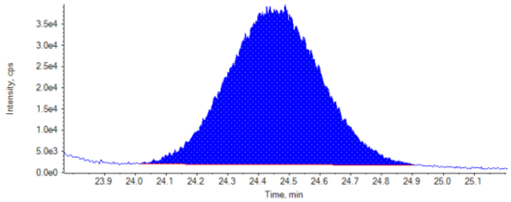


| Compound Name: | F3 (342.000/159.100 Da) |
|---|---|
| Expected RT: | 24.5 |
| Actual RT: | 24.5 |
| Equation: | At least 2 points are required to calculate regression. |
| Area Counts: | 8.11e+005 |
| ISTD Area Counts: | N/A |
| Amount: | 0.00 (ng/mL) |

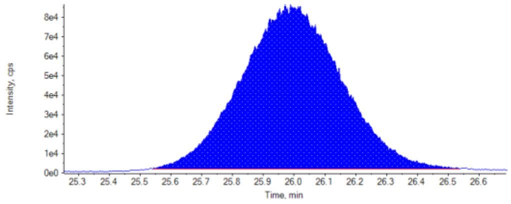


| Compound Name: | F4 (342.000/159.100 Da) |
|---|---|
| Expected RT: | 26.0 |
| Actual RT: | 26.0 |
| Equation: | At least 2 points are required to calculate regression. |
| Area Counts: | 2.00e+006 |
| ISTD Area Counts: | N/A |
| Amount: | 0.00 (ng/mL) |

Figure S16 The LC chromatogram of plums treated with 0.020 a.i. g/L propiconazole at 30 d (20°C)

Created with No data for MQ Created With
Printed:No data for MQ Report Generation Date

Figure Caption
Figure_17_SuppInfo.

| Data File | 20220822 BHZ-YP-4du.wiff | Result Table | 4-0.004-5d-3.rdb |
|---|---|---|---|
| Acquisition Date | 8/23/2022 4:51:18 AM | Algorithm Used | MQL |
| Acquisition Method | BHZ method.dam | Instrument Name | AB SCIEX Triple Quad 4500 |
| Project | DPY | Processing Method | No data for Processing Method |

**Sample Name:** 0.004-5d-3 **Vial #:** 25

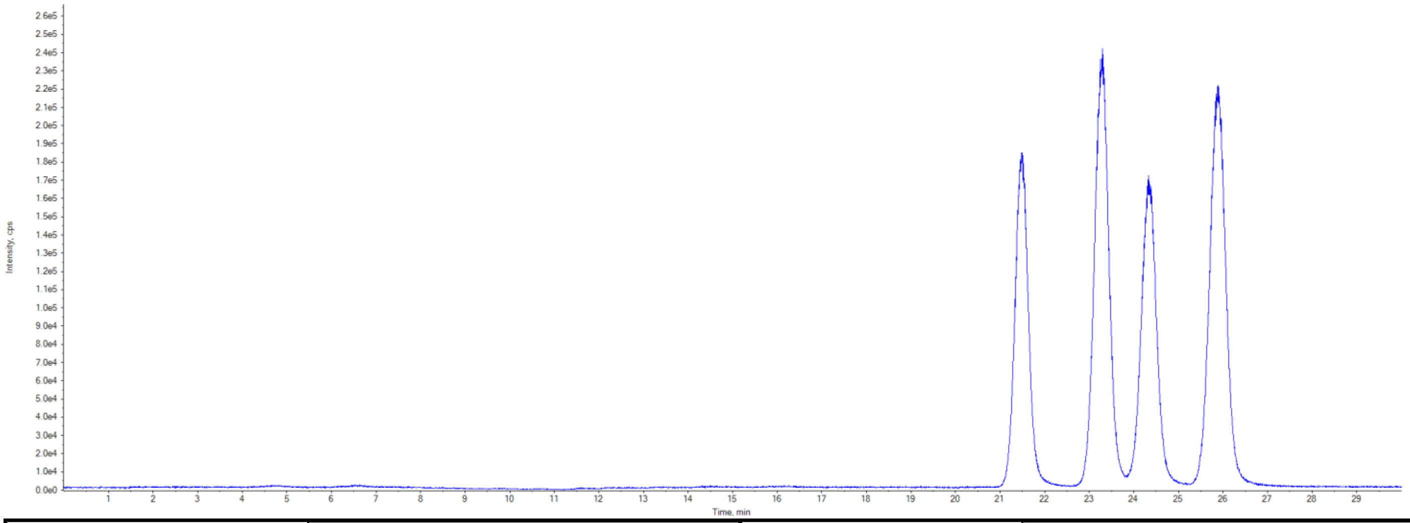

| Sample Name | 0.004-5d-3 | Injection Vial | 25 |
|---|---|---|---|
| Data File | 20220822 BHZ-YP-4du.wiff | Injection Volume | 10 |
| Acquisition Date | 8/23/2022 4:51:18 AM | Algorithm Used | MQL |
| Acquisition Method | BHZ method.dam | Sample Type | Unknown |
| Instrument Name | AB SCIEX Triple Quad 4500 | Result Table | 4-0.004-5d-3.rdb |
| Sample ID | No data for Sample ID | Dilution Factor | 1.00 |
| Sample Comment | No data for Sample Comment | Weight to Volume | 0.00 |

Approved By (Date and Initials):________________________________________.

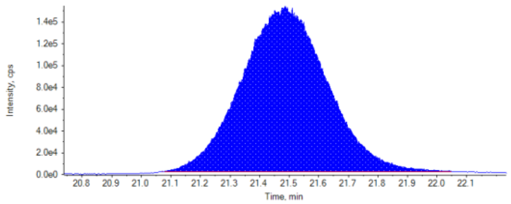

| Compound Name: | F1 (342.000/159.100 Da) |
|---|---|
| Expected RT: | 21.5 |
| Actual RT: | 21.5 |
| Equation: | At least 2 points are required to calculate regression. |
| Area Counts: | 3.21e+006 |
| ISTD Area Counts: | N/A |
| Amount: | 0.00 (ng/mL) |

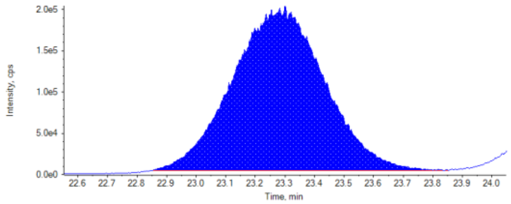


| Compound Name: | F2 (342.000/159.100 Da) |
|---|---|
| Expected RT: | 23.3 |
| Actual RT: | 23.3 |
| Equation: | At least 2 points are required to calculate regression. |
| Area Counts: | 4.38e+006 |
| ISTD Area Counts: | N/A |
| Amount: | 0.00 (ng/mL) |

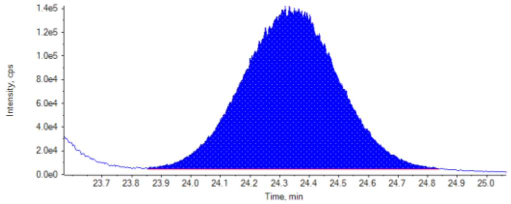


| Compound Name: | F3 (342.000/159.100 Da) |
|---|---|
| Expected RT: | 24.3 |
| Actual RT: | 24.3 |
| Equation: | At least 2 points are required to calculate regression. |
| Area Counts: | 3.16e+006 |
| ISTD Area Counts: | N/A |
| Amount: | 0.00 (ng/mL) |

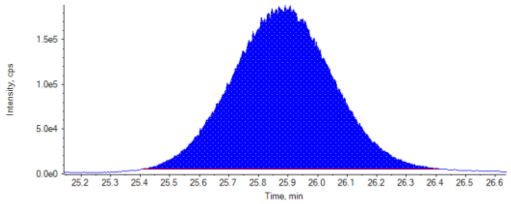


| Compound Name: | F4 (342.000/159.100 Da) |
|---|---|
| Expected RT: | 25.9 |
| Actual RT: | 25.9 |
| Equation: | At least 2 points are required to calculate regression. |
| Area Counts: | 4.64e+006 |
| ISTD Area Counts: | N/A |
| Amount: | 0.00 (ng/mL) |

Figure S17 The LC chromatogram of plums treated with 0.004 a.i. g/L propiconazole at 5 d (4℃)

Created with *No data for MQ Created With*
Printed:*No data for MQ Report Generation Date*

Figure Caption
Figure_18_SuppInfo.

| | | | |
|---|---|---|---|
| **Data File** | 20220822 BHZ-YP-4du.wiff | **Result Table** | 4-0.004-10d-3.rdb |
| **Acquisition Date** | 8/23/2022 3:16:55 AM | **Algorithm Used** | MQL |
| **Acquisition Method** | BHZ method.dam | **Instrument Name** | AB SCIEX Triple Quad 4500 |
| **Project** | DPY | **Processing Method** | *No data for Processing Method* |

**Sample Name:** 0.004-10d-3 **Vial #:** 22

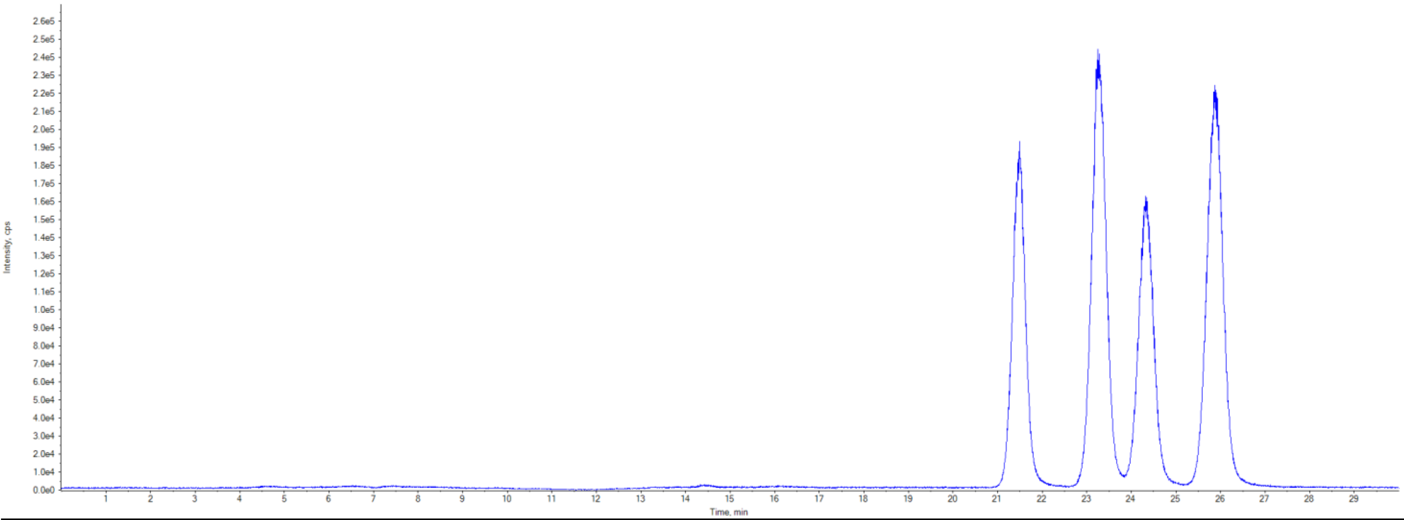


| | | | |
|---|---|---|---|
| **Sample Name** | 0.004-10d-3 | **Injection Vial** | 22 |
| **Data File** | 20220822 BHZ-YP-4du.wiff | **Injection Volume** | 10 |
| **Acquisition Date** | 8/23/2022 3:16:55 AM | **Algorithm Used** | MQL |
| **Acquisition Method** | BHZ method.dam | **Sample Type** | Unknown |
| **Instrument Name** | AB SCIEX Triple Quad 4500 | **Result Table** | 4-0.04-10d-3.rdb |
| **Sample ID** | *No data for Sample ID* | **Dilution Factor** | 1.00 |
| **Sample Comment** | *No data for Sample Comment* | **Weight to Volume** | 0.00 |

Approved By (Date and Initials):_______________________________________________.

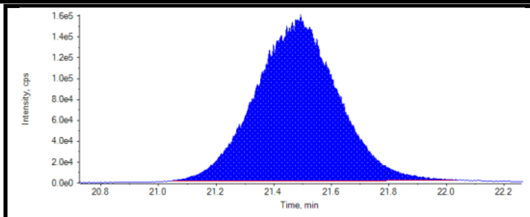


| | |
|---|---|
| **Compound Name:** | F1 **(**342.000/159.100 Da**)** |
| Expected RT: | 21.5 |
| Actual RT: | 21.5 |
| Equation: | At least 2 points are required to calculate regression. |
| Area Counts: | 3.20e+006 |
| ISTD Area Counts: | N/A |
| Amount: | 0.00 (ng/mL) |

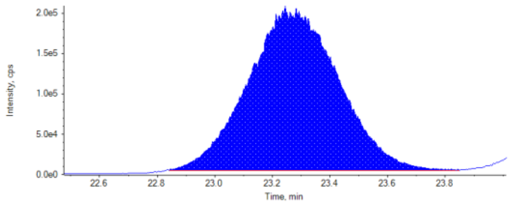


| **Compound Name:** | F2 **(**342.000/159.100 Da**)** |
|---|---|
| Expected RT: | 23.2 |
| Actual RT: | 23.2 |
| Equation: | At least 2 points are required to calculate regression. |
| Area Counts: | 4.49e+006 |
| ISTD Area Counts: | N/A |
| Amount: | 0.00 (ng/mL) |

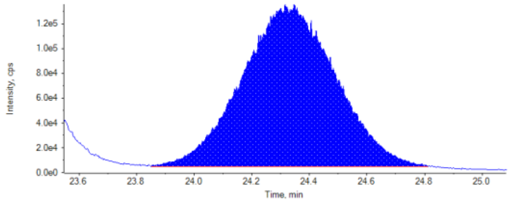


| **Compound Name:** | F3 **(**342.000/159.100 Da**)** |
|---|---|
| Expected RT: | 24.3 |
| Actual RT: | 24.3 |
| Equation: | At least 2 points are required to calculate regression. |
| Area Counts: | 3.00e+006 |
| ISTD Area Counts: | N/A |
| Amount: | 0.00 (ng/mL) |

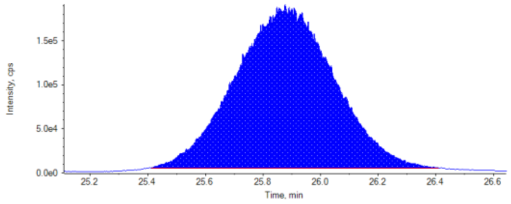


| **Compound Name:** | F4 **(**342.000/159.100 Da**)** |
|---|---|
| Expected RT: | 25.9 |
| Actual RT: | 25.9 |
| Equation: | At least 2 points are required to calculate regression. |
| Area Counts: | 4.72e+006 |
| ISTD Area Counts: | N/A |
| Amount: | 0.00 (ng/mL) |

Figure S18 The LC chromatogram of plums treated with 0.004 a.i. g/L propiconazole at 10 d (4℃)

Figure Caption
Figure_19_SuppInfo.

| | | | |
|---|---|---|---|
| **Data File** | 20220822 BHZ-YP-4du.wiff | **Result Table** | 4-0.004-15d-1.rdb |
| **Acquisition Date** | 8/23/2022 12:39:37 AM | **Algorithm Used** | MQL |
| **Acquisition Method** | BHZ method.dam | **Instrument Name** | AB SCIEX Triple Quad 4500 |
| **Project** | DPY | **Processing Method** | No data for Processing Method |

**Sample Name:** 0.004-15d-1 **Vial #:** 17

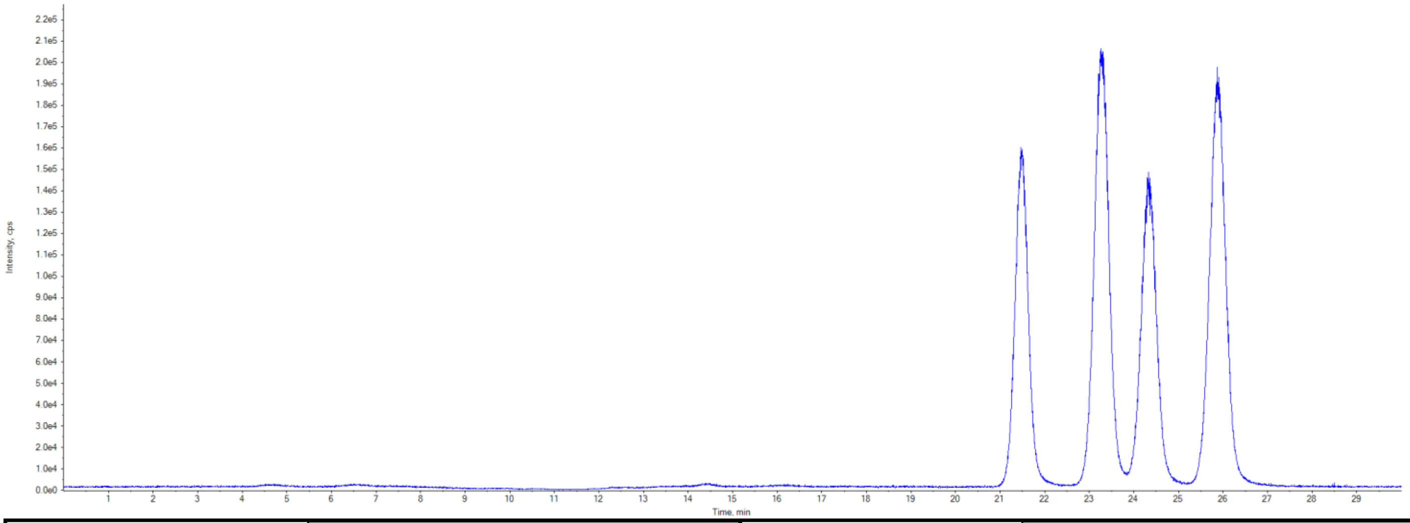


| | | | |
|---|---|---|---|
| **Sample Name** | 0.004-15d-1 | **Injection Vial** | 17 |
| **Data File** | 20220822 BHZ-YP-4du.wiff | **Injection Volume** | 10 |
| **Acquisition Date** | 8/23/2022 12:39:37 AM | **Algorithm Used** | MQL |
| **Acquisition Method** | BHZ method.dam | **Sample Type** | Unknown |
| **Instrument Name** | AB SCIEX Triple Quad 4500 | **Result Table** | 4-0.004-15d-1.rdb |
| **Sample ID** | No data for Sample ID | **Dilution Factor** | 1.00 |
| **Sample Comment** | No data for Sample Comment | **Weight to Volume** | 0.00 |

Approved By (Date and Initials):______________________________.

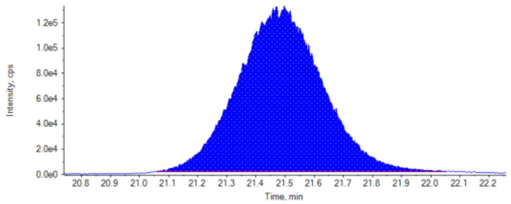


| | |
|---|---|
| **Compound Name:** | F1 **(**342.000/159.100 Da**)** |
| Expected RT: | 21.5 |
| Actual RT: | 21.5 |
| Equation: | At least 2 points are required to calculate regression. |
| Area Counts: | 2.76e+006 |
| ISTD Area Counts: | N/A |
| Amount: | 0.00 (ng/mL) |

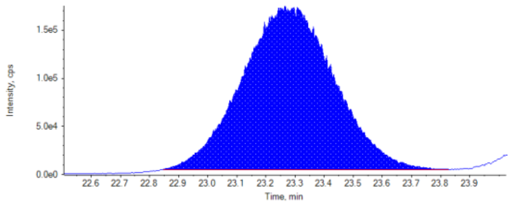


| **Compound Name:** | F2 **(**342.000/159.100 Da**)** |
|---|---|
| Expected RT: | 23.3 |
| Actual RT: | 23.3 |
| Equation: | At least 2 points are required to calculate regression. |
| Area Counts: | 3.87e+006 |
| ISTD Area Counts: | N/A |
| Amount: | 0.00 (ng/mL) |

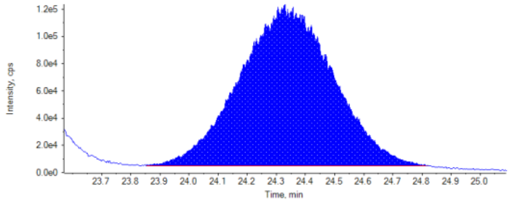


| **Compound Name:** | F3 **(**342.000/159.100 Da**)** |
|---|---|
| Expected RT: | 24.3 |
| Actual RT: | 24.3 |
| Equation: | At least 2 points are required to calculate regression. |
| Area Counts: | 2.72e+006 |
| ISTD Area Counts: | N/A |
| Amount: | 0.00 (ng/mL) |

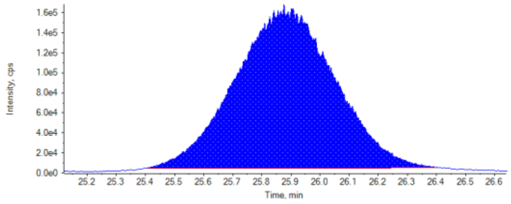


| **Compound Name:** | F4 **(**342.000/159.100 Da**)** |
|---|---|
| Expected RT: | 25.9 |
| Actual RT: | 25.9 |
| Equation: | At least 2 points are required to calculate regression. |
| Area Counts: | 4.09e+006 |
| ISTD Area Counts: | N/A |
| Amount: | 0.00 (ng/mL) |

Figure S19 The LC chromatogram of plums treated with 0.004 a.i. g/L propiconazole at 15 d (4℃)

Created with No data for MQ Created With
Printed:No data for MQ Report Generation Date

Figure Caption
Figure_20_SuppInfo.

| | | | |
|---|---|---|---|
| **Data File** | 20220822 BHZ-YP-4du.wiff | **Result Table** | 4-0.004-20d-2.rdb |
| **Acquisition Date** | 8/22/2022 11:36:42 PM | **Algorithm Used** | MQL |
| **Acquisition Method** | BHZ method.dam | **Instrument Name** | AB SCIEX Triple Quad 4500 |
| **Project** | DPY | **Processing Method** | No data for Processing Method |

**Sample Name:** 0.004-20d-2 **Vial #:** 15

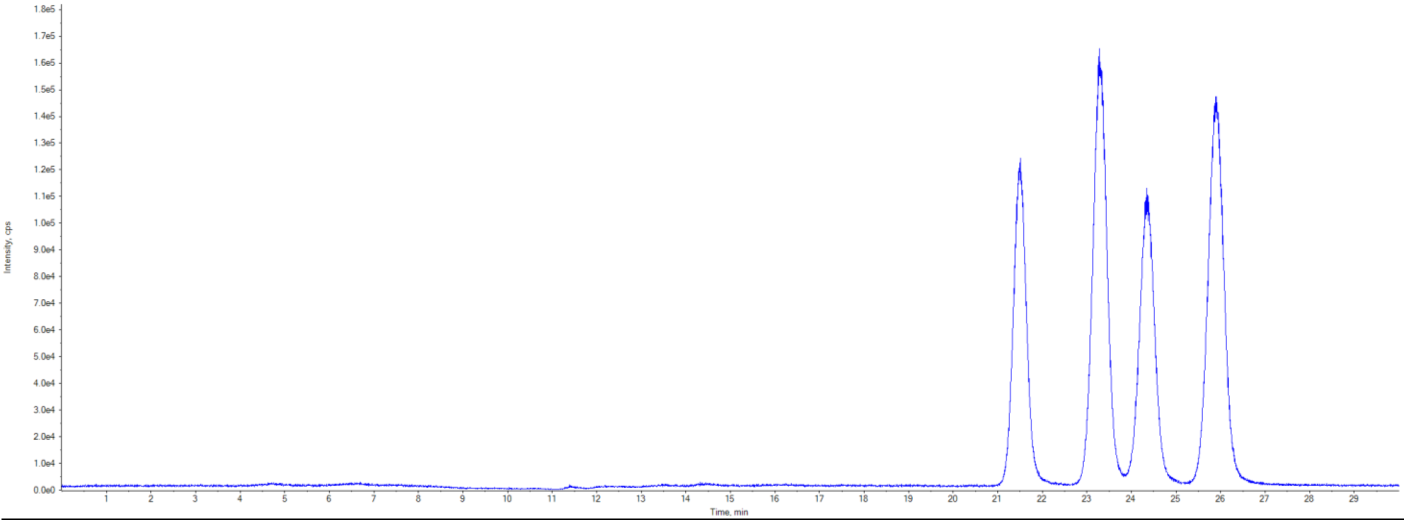

| | | | |
|---|---|---|---|
| **Sample Name** | 0.004-20d-2 | **Injection Vial** | 15 |
| **Data File** | 20220822 BHZ-YP-4du.wiff | **Injection Volume** | 10 |
| **Acquisition Date** | 8/22/2022 11:36:42 PM | **Algorithm Used** | MQL |
| **Acquisition Method** | BHZ method.dam | **Sample Type** | Unknown |
| **Instrument Name** | AB SCIEX Triple Quad 4500 | **Result Table** | 4-0.004-20d-2.rdb |
| **Sample ID** | No data for Sample ID | **Dilution Factor** | 1.00 |
| **Sample Comment** | No data for Sample Comment | **Weight to Volume** | 0.00 |

Approved By (Date and Initials):______________________________________________.

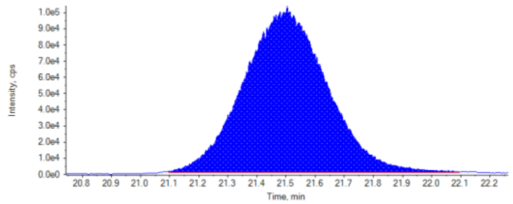

| | |
|---|---|
| **Compound Name:** | F1 **(**342.000/159.100 Da**)** |
| Expected RT: | 21.5 |
| Actual RT: | 21.5 |
| Equation: | At least 2 points are required to calculate regression. |
| Area Counts: | 2.07e+006 |
| ISTD Area Counts: | N/A |
| Amount: | 0.00 (ng/mL) |

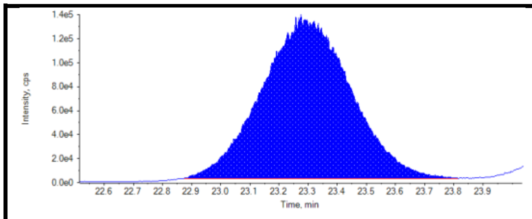


| **Compound Name:** | F2 **(**342.000/159.100 Da**)** |
|---|---|
| Expected RT: | 23.3 |
| Actual RT: | 23.3 |
| Equation: | At least 2 points are required to calculate regression. |
| Area Counts: | 2.93e+006 |
| ISTD Area Counts: | N/A |
| Amount: | 0.00 (ng/mL) |

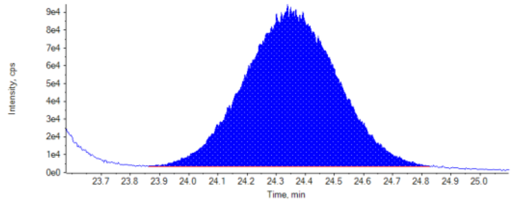


| **Compound Name:** | F3 **(**342.000/159.100 Da**)** |
|---|---|
| Expected RT: | 24.3 |
| Actual RT: | 24.3 |
| Equation: | At least 2 points are required to calculate regression. |
| Area Counts: | 2.01e+006 |
| ISTD Area Counts: | N/A |
| Amount: | 0.00 (ng/mL) |

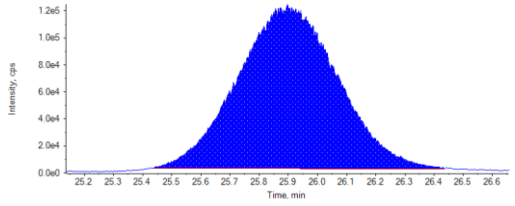


| **Compound Name:** | F4 **(**342.000/159.100 Da**)** |
|---|---|
| Expected RT: | 25.9 |
| Actual RT: | 25.9 |
| Equation: | At least 2 points are required to calculate regression. |
| Area Counts: | 3.03e+006 |
| ISTD Area Counts: | N/A |
| Amount: | 0.00 (ng/mL) |

Figure S20 The LC chromatogram of plums treated with 0.004 a.i. g/L propiconazole at 20 d (4℃)

Created with No data for MQ Created With
Printed:No data for MQ Report Generation Date

Figure Caption
Figure_21_SuppInfo.

| | | | |
|---|---|---|---|
| **Data File** | 20220822 BHZ-YP-4du.wiff | **Result Table** | 4-0.004-25d-2.rdb |
| **Acquisition Date** | 8/22/2022 10:02:19 PM | **Algorithm Used** | MQL |
| **Acquisition Method** | BHZ method.dam | **Instrument Name** | AB SCIEX Triple Quad 4500 |
| **Project** | DPY | **Processing Method** | No data for Processing Method |

**Sample Name:** 0.004-25d-2 **Vial #:** 12

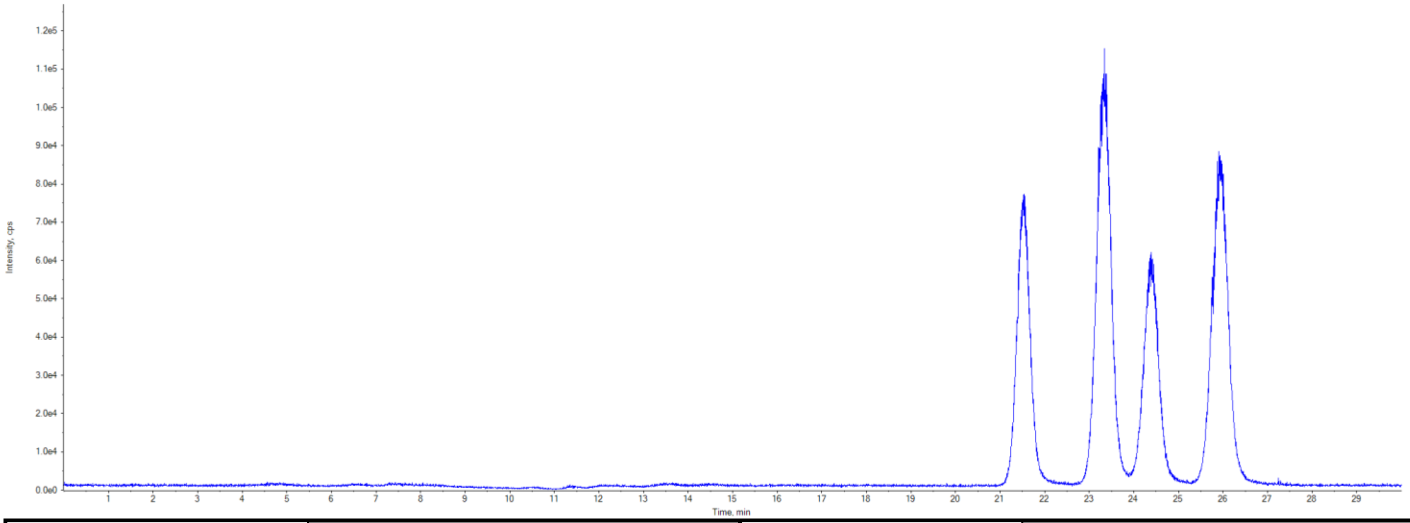


| | | | |
|---|---|---|---|
| **Sample Name** | 0.004-25d-2 | **Injection Vial** | 12 |
| **Data File** | 20220822 BHZ-YP-4du.wiff | **Injection Volume** | 10 |
| **Acquisition Date** | 8/22/2022 10:02:19 PM | **Algorithm Used** | MQL |
| **Acquisition Method** | BHZ method.dam | **Sample Type** | Unknown |
| **Instrument Name** | AB SCIEX Triple Quad 4500 | **Result Table** | 4-0.004-25d-2.rdb |
| **Sample ID** | No data for Sample ID | **Dilution Factor** | 1.00 |
| **Sample Comment** | No data for Sample Comment | **Weight to Volume** | 0.00 |

Approved By (Date and Initials):_______________________________________________.

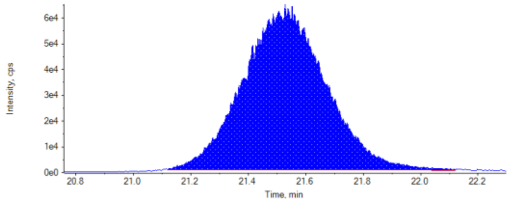


| | |
|---|---|
| **Compound Name:** | F1 **(**342.000/159.100 Da**)** |
| Expected RT: | 21.5 |
| Actual RT: | 21.5 |
| Equation: | At least 2 points are required to calculate regression. |
| Area Counts: | 1.28e+006 |
| ISTD Area Counts: | N/A |
| Amount: | 0.00 (ng/mL) |

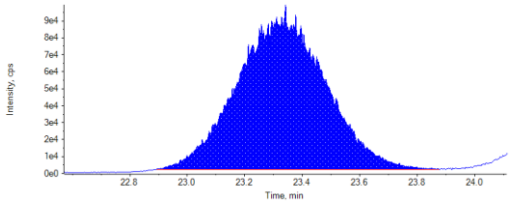


| Compound Name: | F2 (342.000/159.100 Da) |
|---|---|
| Expected RT: | 23.3 |
| Actual RT: | 23.3 |
| Equation: | At least 2 points are required to calculate regression. |
| Area Counts: | 2.00e+006 |
| ISTD Area Counts: | N/A |
| Amount: | 0.00 (ng/mL) |

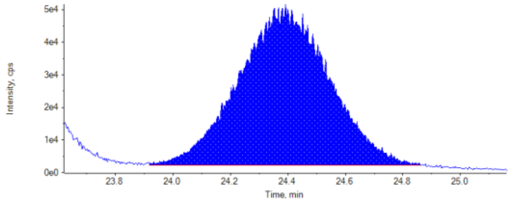


| Compound Name: | F3 (342.000/159.100 Da) |
|---|---|
| Expected RT: | 24.4 |
| Actual RT: | 24.4 |
| Equation: | At least 2 points are required to calculate regression. |
| Area Counts: | 1.09e+006 |
| ISTD Area Counts: | N/A |
| Amount: | 0.00 (ng/mL) |

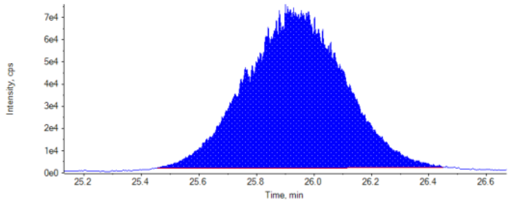


| Compound Name: | F4 (342.000/159.100 Da) |
|---|---|
| Expected RT: | 25.9 |
| Actual RT: | 25.9 |
| Equation: | At least 2 points are required to calculate regression. |
| Area Counts: | 1.79e+006 |
| ISTD Area Counts: | N/A |
| Amount: | 0.00 (ng/mL) |

Figure S21 The LC chromatogram of plums treated with 0.004 a.i. g/L propiconazole at 25 d (4℃)

Created with No data for MQ Created With
Printed:No data for MQ Report Generation Date

Figure Caption
Figure_22_SuppInfo.

| | | | |
|---|---|---|---|
| **Data File** | 20220822 BHZ-YP-4du.wiff | **Result Table** | 4-0.004-35d-3.rdb |
| **Acquisition Date** | 8/22/2022 7:25:01 PM | **Algorithm Used** | MQL |
| **Acquisition Method** | BHZ  method.dam | **Instrument Name** | AB SCIEX Triple Quad 4500 |
| **Project** | DPY | **Processing Method** | No data for Processing Method |

**Sample Name:** 0.004-35d-3 **Vial #:** 7

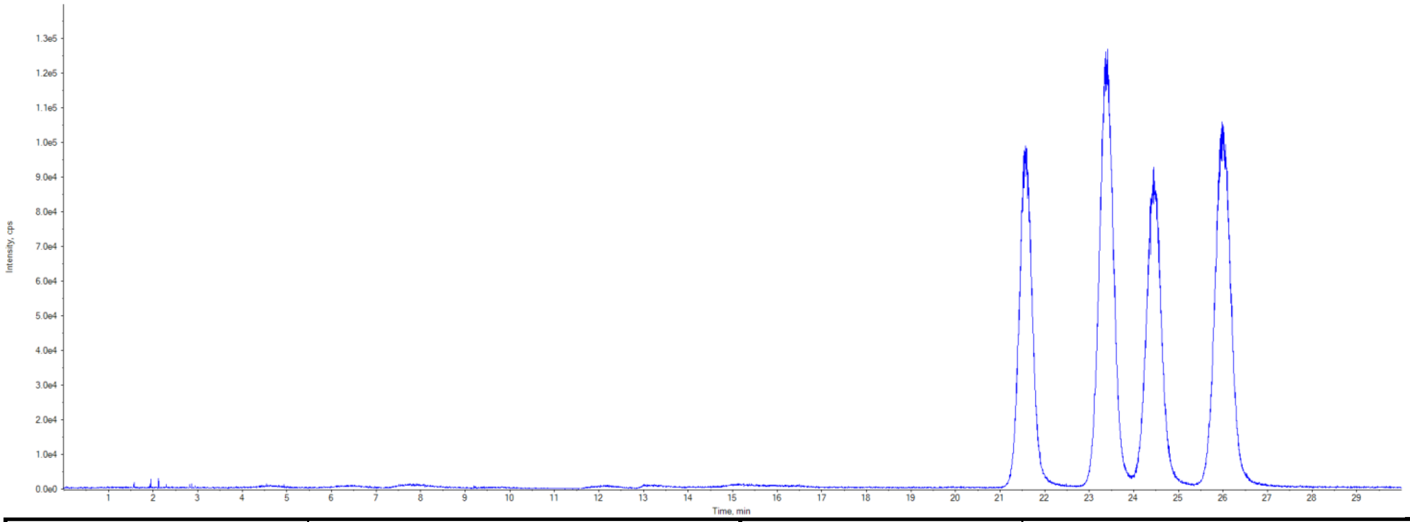


| | | | |
|---|---|---|---|
| **Sample Name** | 0.004-35d-3 | **Injection Vial** | 7 |
| **Data File** | 20220822 BHZ-YP-4du.wiff | **Injection Volume** | 10 |
| **Acquisition Date** | 8/22/2022 7:25:01 PM | **Algorithm Used** | MQL |
| **Acquisition Method** | BHZ  method.dam | **Sample Type** | Unknown |
| **Instrument Name** | AB SCIEX Triple Quad 4500 | **Result Table** | 4-0.004-35d-3.rdb |
| **Sample ID** | No data for Sample ID | **Dilution Factor** | 1.00 |
| **Sample Comment** | No data for Sample Comment | **Weight to Volume** | 0.00 |

Approved By (Date and Initials):____________________________________________.

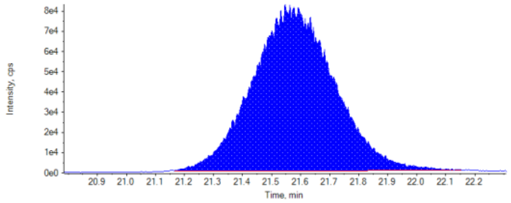


| | |
|---|---|
| **Compound Name:** | F1 **(**342.000/159.100 Da**)** |
| Expected RT: | 21.5 |
| Actual RT: | 21.5 |
| Equation: | At least 2 points are required to calculate regression. |
| Area Counts: | 1.63e+006 |
| ISTD Area Counts: | N/A |
| Amount: | 0.00 (ng/mL) |

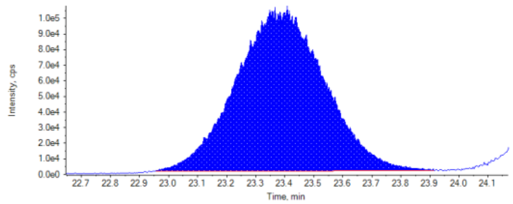


| **Compound Name:** | F2 **(**342.000/159.100 Da**)** |
|---|---|
| Expected RT: | 23.4 |
| Actual RT: | 23.4 |
| Equation: | At least 2 points are required to calculate regression. |
| Area Counts: | 2.23e+006 |
| ISTD Area Counts: | N/A |
| Amount: | 0.00 (ng/mL) |

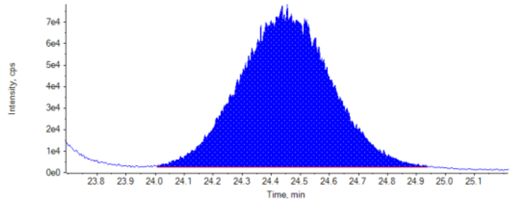


| **Compound Name:** | F3 **(**342.000/159.100 Da**)** |
|---|---|
| Expected RT: | 24.5 |
| Actual RT: | 24.5 |
| Equation: | At least 2 points are required to calculate regression. |
| Area Counts: | 1.64e+006 |
| ISTD Area Counts: | N/A |
| Amount: | 0.00 (ng/mL) |

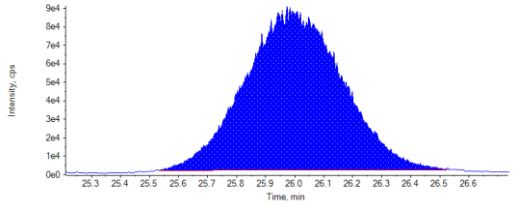


| **Compound Name:** | F4 **(**342.000/159.100 Da**)** |
|---|---|
| Expected RT: | 26.0 |
| Actual RT: | 26.0 |
| Equation: | At least 2 points are required to calculate regression. |
| Area Counts: | 2.16e+006 |
| ISTD Area Counts: | N/A |
| Amount: | 0.00 (ng/mL) |

Figure S22 The LC chromatogram of plums treated with 0.004 a.i. g/L propiconazole at 35 d (4℃)

Created with No data for MQ Created With
Printed:No data for MQ Report Generation Date

Figure Caption
Figure_23_SuppInfo.

| | | | |
|---|---|---|---|
| **Data File** | BHZ 20220911.wiff | **Result Table** | 4-0.004-45d-3.rdb |
| **Acquisition Date** | 9/11/2022 8:18:00 PM | **Algorithm Used** | MQL |
| **Acquisition Method** | BHZ  method.dam | **Instrument Name** | AB SCIEX Triple Quad 4500 |
| **Project** | DPY | **Processing Method** | No data for Processing Method |

**Sample Name:** 4-0.004-45d-3 **Vial #:** 23

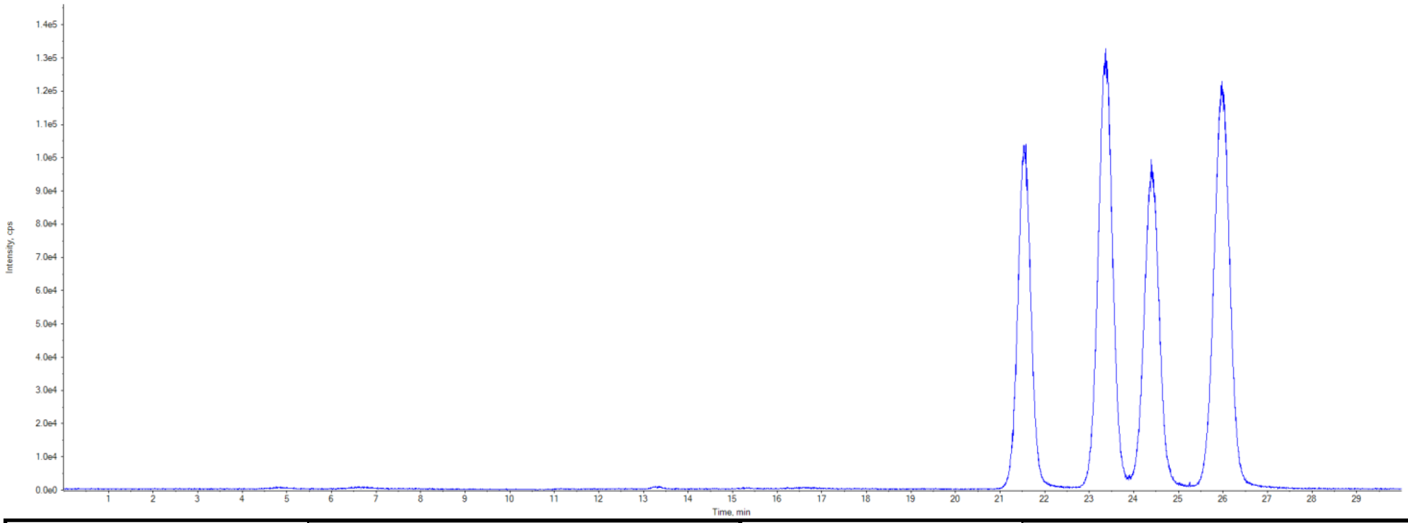


| | | | |
|---|---|---|---|
| **Sample Name** | 4-0.004-45d-3 | **Injection Vial** | 23 |
| **Data File** | BHZ 20220911.wiff | **Injection Volume** | 10 |
| **Acquisition Date** | 9/11/2022 8:18:00 PM | **Algorithm Used** | MQL |
| **Acquisition Method** | BHZ  method.dam | **Sample Type** | Unknown |
| **Instrument Name** | AB SCIEX Triple Quad 4500 | **Result Table** | 4-0.004-45d-3.rdb |
| **Sample ID** | No data for Sample ID | **Dilution Factor** | 1.00 |
| **Sample Comment** | No data for Sample Comment | **Weight to Volume** | 0.00 |

Approved By (Date and Initials):______________________________________________.

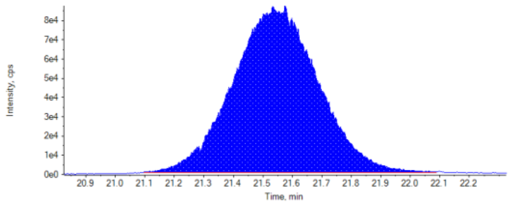


| | |
|---|---|
| **Compound Name:** | F1 **(**342.000/159.100 Da**)** |
| Expected RT: | 21.6 |
| Actual RT: | 21.6 |
| Equation: | At least 2 points are required to calculate regression. |
| Area Counts: | 1.79e+006 |
| ISTD Area Counts: | N/A |
| Amount: | 0.00 (ng/mL) |

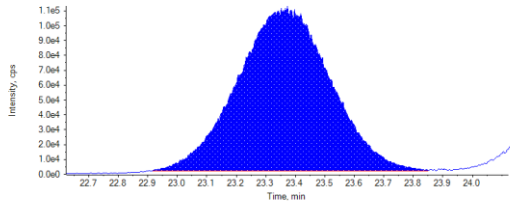


| **Compound Name:** | F2 **(**342.000/159.100 Da**)** |
|---|---|
| Expected RT: | 23.4 |
| Actual RT: | 23.4 |
| Equation: | At least 2 points are required to calculate regression. |
| Area Counts: | 2.45e+006 |
| ISTD Area Counts: | N/A |
| Amount: | 0.00 (ng/mL) |

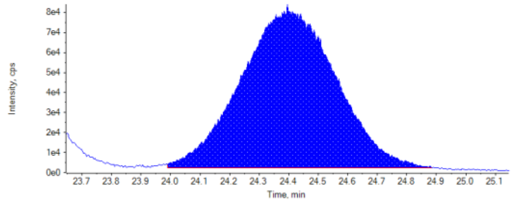


| **Compound Name:** | F3 **(**342.000/159.100 Da**)** |
|---|---|
| Expected RT: | 24.4 |
| Actual RT: | 24.4 |
| Equation: | At least 2 points are required to calculate regression. |
| Area Counts: | 1.82e+006 |
| ISTD Area Counts: | N/A |
| Amount: | 0.00 (ng/mL) |

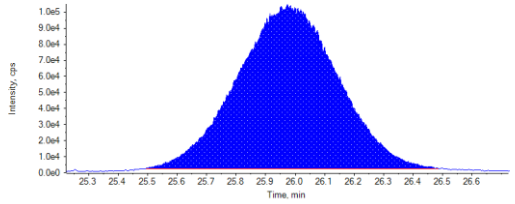


| **Compound Name:** | F4 **(**342.000/159.100 Da**)** |
|---|---|
| Expected RT: | 26.0 |
| Actual RT: | 26.0 |
| Equation: | At least 2 points are required to calculate regression. |
| Area Counts: | 2.55e+006 |
| ISTD Area Counts: | N/A |
| Amount: | 0.00 (ng/mL) |

Figure 23 The LC chromatogram of plums treated with 0.004 a.i. g/L propiconazole at 45 d (4℃)

Created with No data for MQ Created With
Printed:No data for MQ Report Generation Date

Figure Caption
Figure_24_SuppInfo.

| Data File | BHZ 20220911.wiff | Result Table | 4-0.004-55d-3.rdb |
|---|---|---|---|
| Acquisition Date | 9/11/2022 6:43:40 PM | Algorithm Used | MQL |
| Acquisition Method | BHZ  method.dam | Instrument Name | AB SCIEX Triple Quad 4500 |
| Project | DPY | Processing Method | No data for Processing Method |

**Sample Name:** 4-0.004-55d-3 **Vial #:** 20

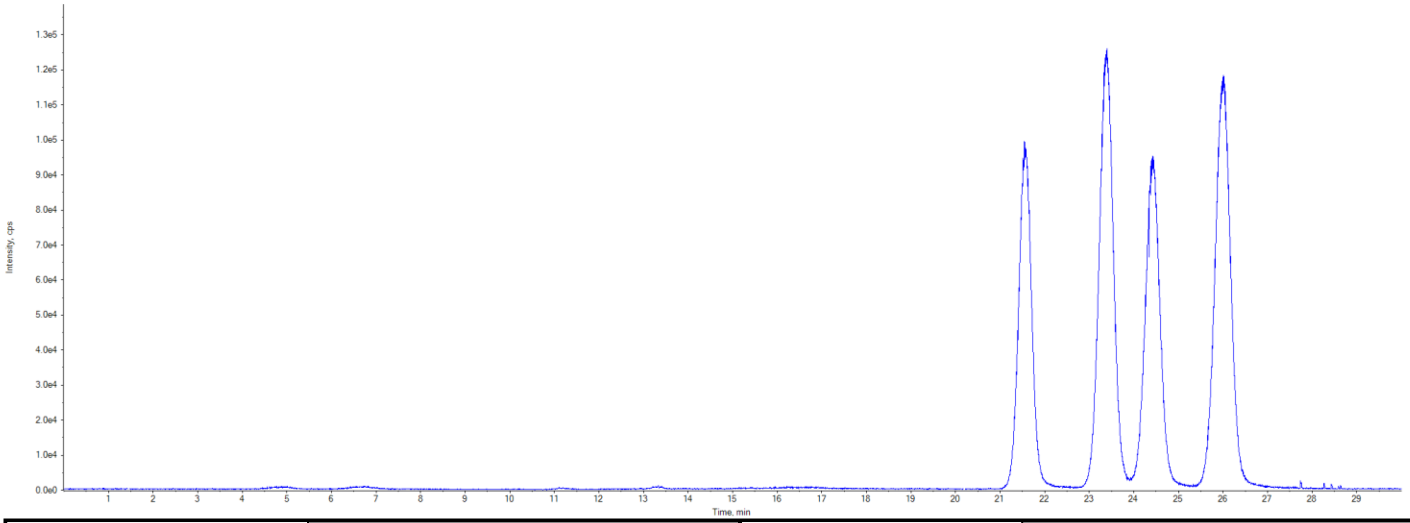


| Sample Name | 4-0.004-55d-3 | Injection Vial | 20 |
|---|---|---|---|
| Data File | BHZ 20220911.wiff | Injection Volume | 10 |
| Acquisition Date | 9/11/2022 6:43:40 PM | Algorithm Used | MQL |
| Acquisition Method | BHZ  method.dam | Sample Type | Unknown |
| Instrument Name | AB SCIEX Triple Quad 4500 | Result Table | 4-0.004-55d-3.rdb |
| Sample ID | No data for Sample ID | Dilution Factor | 1.00 |
| Sample Comment | No data for Sample Comment | Weight to Volume | 0.00 |

Approved By (Date and Initials):________________________________________________.

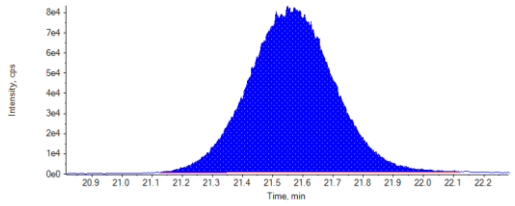


| Compound Name: | F1 (342.000/159.100 Da) |
|---|---|
| Expected RT: | 21.5 |
| Actual RT: | 21.5 |
| Equation: | At least 2 points are required to calculate regression. |
| Area Counts: | 1.67e+006 |
| ISTD Area Counts: | N/A |
| Amount: | 0.00 (ng/mL) |

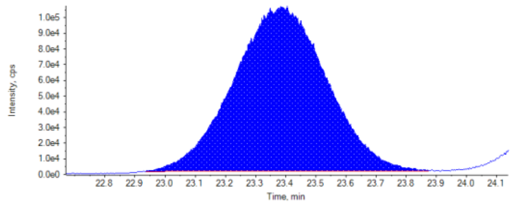


| Compound Name: | F2 (342.000/159.100 Da) |
|---|---|
| Expected RT: | 23.4 |
| Actual RT: | 23.4 |
| Equation: | At least 2 points are required to calculate regression. |
| Area Counts: | 2.31e+006 |
| ISTD Area Counts: | N/A |
| Amount: | 0.00 (ng/mL) |

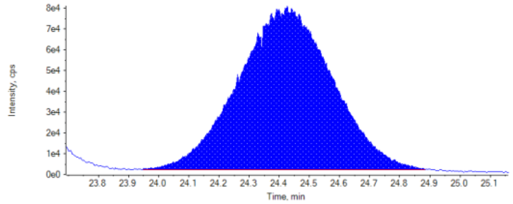


| Compound Name: | F3 (342.000/159.100 Da) |
|---|---|
| Expected RT: | 24.4 |
| Actual RT: | 24.4 |
| Equation: | At least 2 points are required to calculate regression. |
| Area Counts: | 1.74e+006 |
| ISTD Area Counts: | N/A |
| Amount: | 0.00 (ng/mL) |

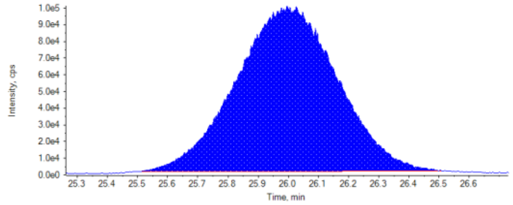


| Compound Name: | F4 (342.000/159.100 Da) |
|---|---|
| Expected RT: | 26.0 |
| Actual RT: | 26.0 |
| Equation: | At least 2 points are required to calculate regression. |
| Area Counts: | 2.42e+006 |
| ISTD Area Counts: | N/A |
| Amount: | 0.00 (ng/mL) |

Figure S24 The LC chromatogram of plums treated with 0.004 a.i. g/L propiconazole at 55 d (4℃)

Created with No data for MQ Created With
Printed:No data for MQ Report Generation Date

Figure Caption
Figure_25_SuppInfo.

| | | | |
|---|---|---|---|
| **Data File** | 20220822 BHZ-YP-4du.wiff | **Result Table** | 4-0.02-5d-2.rdb |
| **Acquisition Date** | 8/22/2022 3:44:47 PM | **Algorithm Used** | MQL |
| **Acquisition Method** | BHZ method.dam | **Instrument Name** | AB SCIEX Triple Quad 4500 |
| **Project** | DPY | **Processing Method** | No data for Processing Method |

**Sample Name:** 0.02-5d-2 **Vial #:** 104

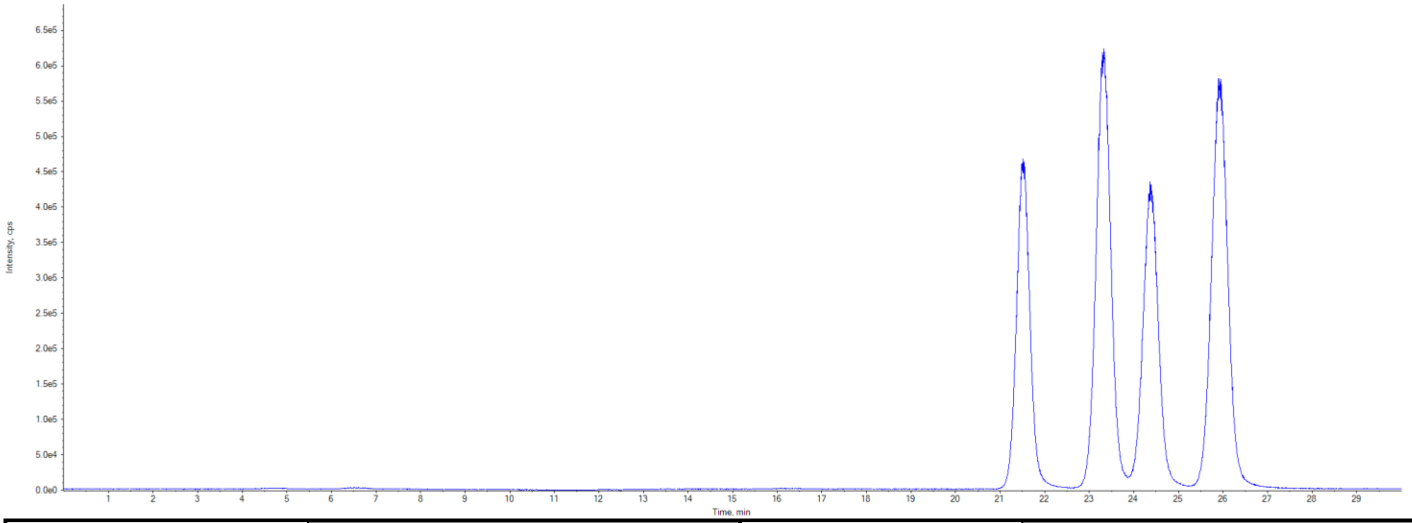


| | | | |
|---|---|---|---|
| **Sample Name** | 0.02-5d-2 | **Injection Vial** | 104 |
| **Data File** | 20220822 BHZ-YP-4du.wiff | **Injection Volume** | 10 |
| **Acquisition Date** | 8/22/2022 3:44:47 PM | **Algorithm Used** | MQL |
| **Acquisition Method** | BHZ method.dam | **Sample Type** | Unknown |
| **Instrument Name** | AB SCIEX Triple Quad 4500 | **Result Table** | 4-0.02-5d-2.rdb |
| **Sample ID** | No data for Sample ID | **Dilution Factor** | 1.00 |
| **Sample Comment** | No data for Sample Comment | **Weight to Volume** | 0.00 |

Approved By (Date and Initials):______________________________________________.

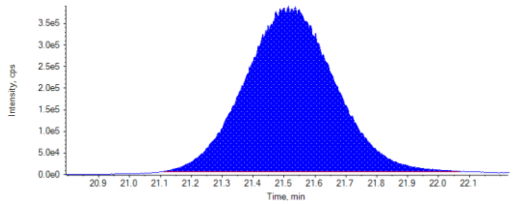


| | |
|---|---|
| **Compound Name:** | F1 **(**342.000/159.100 Da**)** |
| Expected RT: | 21.5 |
| Actual RT: | 21.5 |
| Equation: | At least 2 points are required to calculate regression. |
| Area Counts: | 8.15e+006 |
| ISTD Area Counts: | N/A |
| Amount: | 0.00 (ng/mL) |

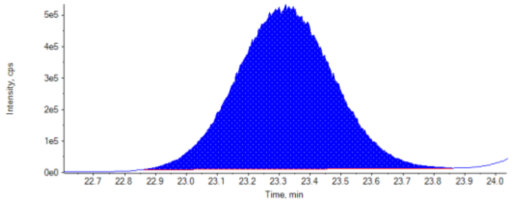


| **Compound Name:** | F2 **(**342.000/159.100 Da**)** |
|---|---|
| Expected RT: | 23.3 |
| Actual RT: | 23.3 |
| Equation: | At least 2 points are required to calculate regression. |
| Area Counts: | 1.17e+007 |
| ISTD Area Counts: | N/A |
| Amount: | 0.00 (ng/mL) |

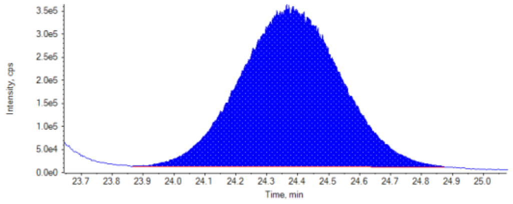


| **Compound Name:** | F3 **(**342.000/159.100 Da**)** |
|---|---|
| Expected RT: | 24.4 |
| Actual RT: | 24.4 |
| Equation: | At least 2 points are required to calculate regression. |
| Area Counts: | 8.09e+006 |
| ISTD Area Counts: | N/A |
| Amount: | 0.00 (ng/mL) |

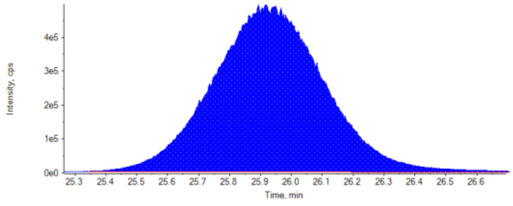


| **Compound Name:** | F4 **(**342.000/159.100 Da**)** |
|---|---|
| Expected RT: | 26.0 |
| Actual RT: | 25.9 |
| Equation: | At least 2 points are required to calculate regression. |
| Area Counts: | 1.24e+007 |
| ISTD Area Counts: | N/A |
| Amount: | 0.00 (ng/mL) |

Figure S25 The LC chromatogram of plums treated with 0.020 a.i. g/L propiconazole at 5 d (4℃)

Figure Caption
Figure_26_SuppInfo.

| | | | |
|---|---|---|---|
| **Data File** | 20220822 BHZ-YP-4du.wiff | **Result Table** | 4-0.02-10d-3.rdb |
| **Acquisition Date** | 8/22/2022 2:41:55 PM | **Algorithm Used** | MQL |
| **Acquisition Method** | BHZ method.dam | **Instrument Name** | AB SCIEX Triple Quad 4500 |
| **Project** | DPY | **Processing Method** | No data for Processing Method |

**Sample Name:** 0.02-10d-3 **Vial #:** 102

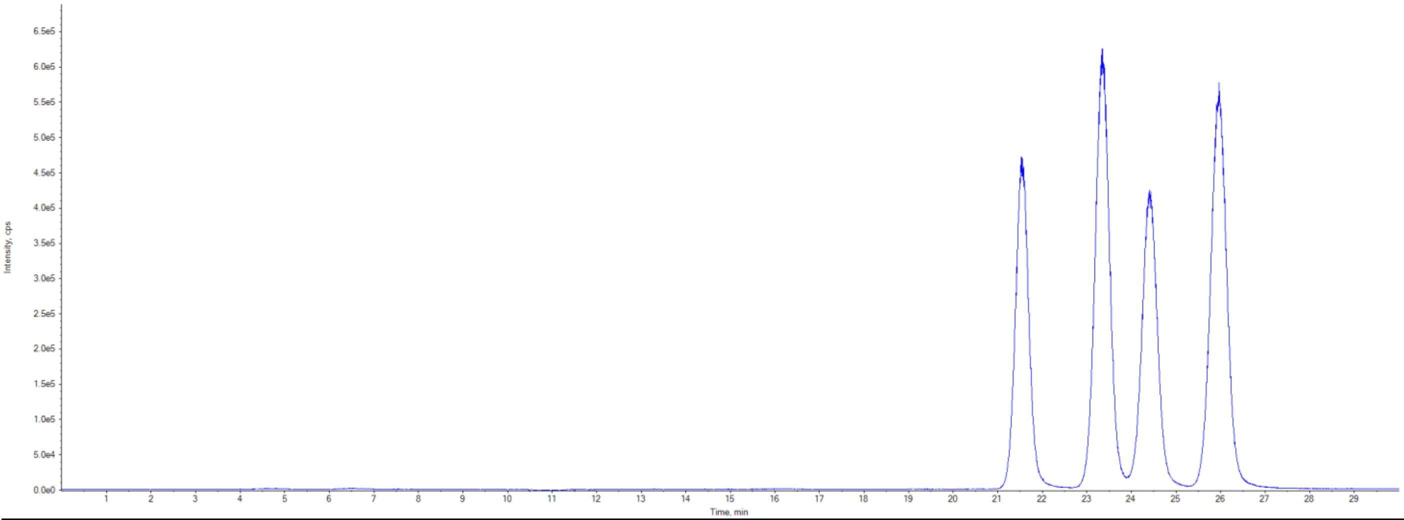


| | | | |
|---|---|---|---|
| **Sample Name** | 0.02-10d-3 | **Injection Vial** | 102 |
| **Data File** | 20220822 BHZ-YP-4du.wiff | **Injection Volume** | 10 |
| **Acquisition Date** | 8/22/2022 2:41:55 PM | **Algorithm Used** | MQL |
| **Acquisition Method** | BHZ method.dam | **Sample Type** | Unknown |
| **Instrument Name** | AB SCIEX Triple Quad 4500 | **Result Table** | 4-0.02-10d-3.rdb |
| **Sample ID** | No data for Sample ID | **Dilution Factor** | 1.00 |
| **Sample Comment** | No data for Sample Comment | **Weight to Volume** | 0.00 |

Approved By (Date and Initials):________________________________________________.

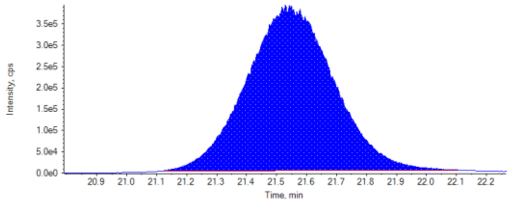


| | |
|---|---|
| **Compound Name:** | F1 **(**342.000/159.100 Da**)** |
| Expected RT: | 21.5 |
| Actual RT: | 21.5 |
| Equation: | At least 2 points are required to calculate regression. |
| Area Counts: | 8.14e+006 |
| ISTD Area Counts: | N/A |
| Amount: | 0.00 (ng/mL) |

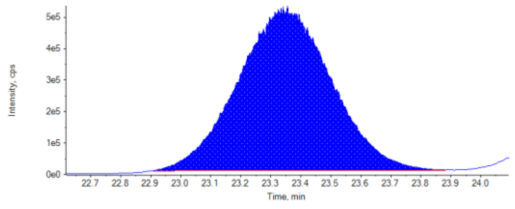


| **Compound Name:** | F2 **(**342.000/159.100 Da**)** |
|---|---|
| Expected RT: | 23.4 |
| Actual RT: | 23.4 |
| Equation: | At least 2 points are required to calculate regression. |
| Area Counts: | 1.14e+007 |
| ISTD Area Counts: | N/A |
| Amount: | 0.00 (ng/mL) |

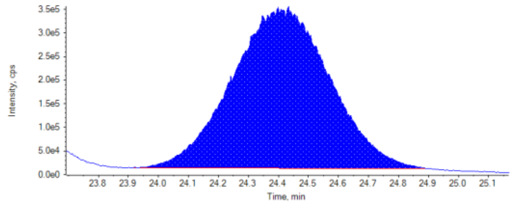


| **Compound Name:** | F3 **(**342.000/159.100 Da**)** |
|---|---|
| Expected RT: | 24.4 |
| Actual RT: | 24.4 |
| Equation: | At least 2 points are required to calculate regression. |
| Area Counts: | 7.96e+006 |
| ISTD Area Counts: | N/A |
| Amount: | 0.00 (ng/mL) |

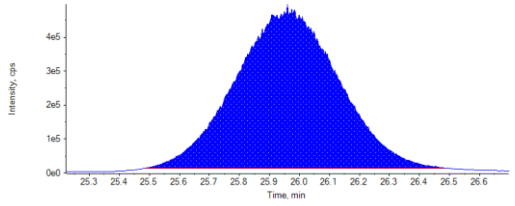


| **Compound Name:** | F4 **(**342.000/159.100 Da**)** |
|---|---|
| Expected RT: | 26.0 |
| Actual RT: | 26.0 |
| Equation: | At least 2 points are required to calculate regression. |
| Area Counts: | 1.19e+007 |
| ISTD Area Counts: | N/A |
| Amount: | 0.00 (ng/mL) |

Figure S26 The LC chromatogram of plums treated with 0.020 a.i. g/L propiconazole at 10 d (4℃)

Figure Caption
Figure_27_SuppInfo.

| | | | |
|---|---|---|---|
| **Data File** | 20220822 BHZ-YP-4du.wiff | **Result Table** | 4-0.02-15d-2.rdb |
| **Acquisition Date** | 8/22/2022 12:36:07 PM | **Algorithm Used** | MQL |
| **Acquisition Method** | BHZ  method.dam | **Instrument Name** | AB SCIEX Triple Quad 4500 |
| **Project** | DPY | **Processing Method** | No data for Processing Method |

**Sample Name:** 0.02-15d-2 **Vial #:** 98

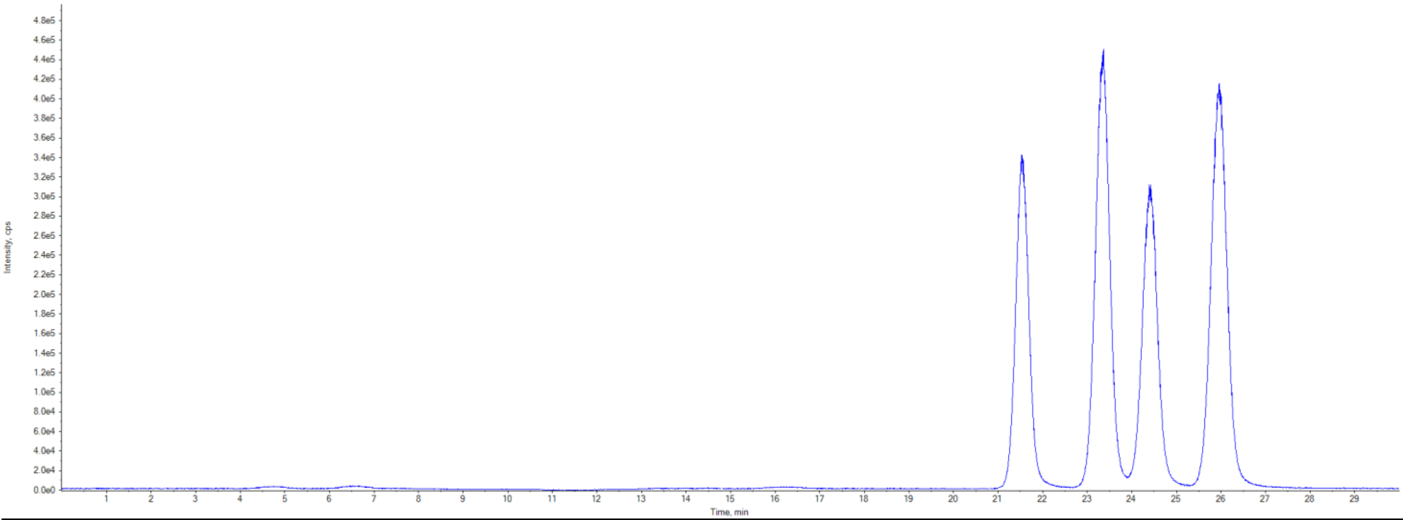


| | | | |
|---|---|---|---|
| **Sample Name** | 0.02-15d-2 | **Injection Vial** | 98 |
| **Data File** | 20220822 BHZ-YP-4du.wiff | **Injection Volume** | 10 |
| **Acquisition Date** | 8/22/2022 12:36:07 PM | **Algorithm Used** | MQL |
| **Acquisition Method** | BHZ  method.dam | **Sample Type** | Unknown |
| **Instrument Name** | AB SCIEX Triple Quad 4500 | **Result Table** | 4-0.02-15d-2.rdb |
| **Sample ID** | No data for Sample ID | **Dilution Factor** | 1.00 |
| **Sample Comment** | No data for Sample Comment | **Weight to Volume** | 0.00 |

Approved By (Date and Initials):______________________________________________.

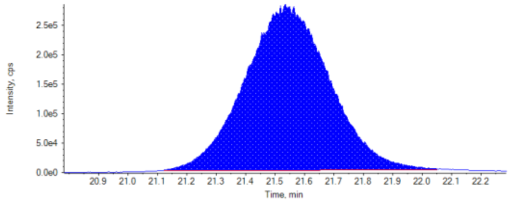


| **Compound Name:** | F1 **(**342.000/159.100 Da**)** |
|---|---|
| Expected RT: | 21.5 |
| Actual RT: | 21.5 |
| Equation: | At least 2 points are required to calculate regression. |
| Area Counts: | 5.87e+006 |
| ISTD Area Counts: | N/A |
| Amount: | 0.00 (ng/mL) |

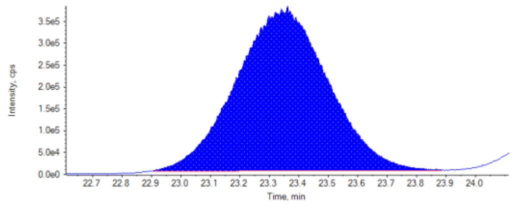


| **Compound Name:** | F2 **(**342.000/159.100 Da**)** |
|---|---|
| Expected RT: | 23.4 |
| Actual RT: | 23.4 |
| Equation: | At least 2 points are required to calculate regression. |
| Area Counts: | 8.22e+006 |
| ISTD Area Counts: | N/A |
| Amount: | 0.00 (ng/mL) |

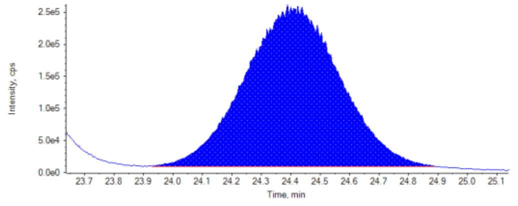


| **Compound Name:** | F3 **(**342.000/159.100 Da**)** |
|---|---|
| Expected RT: | 24.4 |
| Actual RT: | 24.4 |
| Equation: | At least 2 points are required to calculate regression. |
| Area Counts: | 5.78e+006 |
| ISTD Area Counts: | N/A |
| Amount: | 0.00 (ng/mL) |

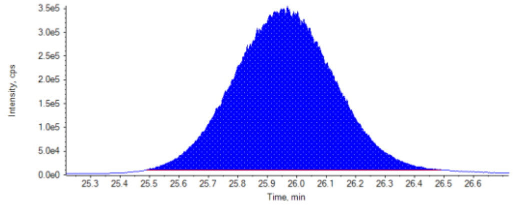


| **Compound Name:** | F4 **(**342.000/159.100 Da**)** |
|---|---|
| Expected RT: | 26.0 |
| Actual RT: | 26.0 |
| Equation: | At least 2 points are required to calculate regression. |
| Area Counts: | 8.74e+006 |
| ISTD Area Counts: | N/A |
| Amount: | 0.00 (ng/mL) |

Figure S27 The LC chromatogram of plums treated with 0.020 a.i. g/L propiconazole at 15 d (4℃)

Created with No data for MQ Created With
Printed:No data for MQ Report Generation Date

Figure Caption
Figure_28_SuppInfo.

| | | | |
|---|---|---|---|
| **Data File** | 20220822 BHZ-YP-4du.wiff | **Result Table** | 4-0.02-20d-1.rdb |
| **Acquisition Date** | 8/22/2022 10:30:23 AM | **Algorithm Used** | MQL |
| **Acquisition Method** | BHZ  method.dam | **Instrument Name** | AB SCIEX Triple Quad 4500 |
| **Project** | DPY | **Processing Method** | No data for Processing Method |

**Sample Name:** 0.02-20d-1 **Vial #:** 94

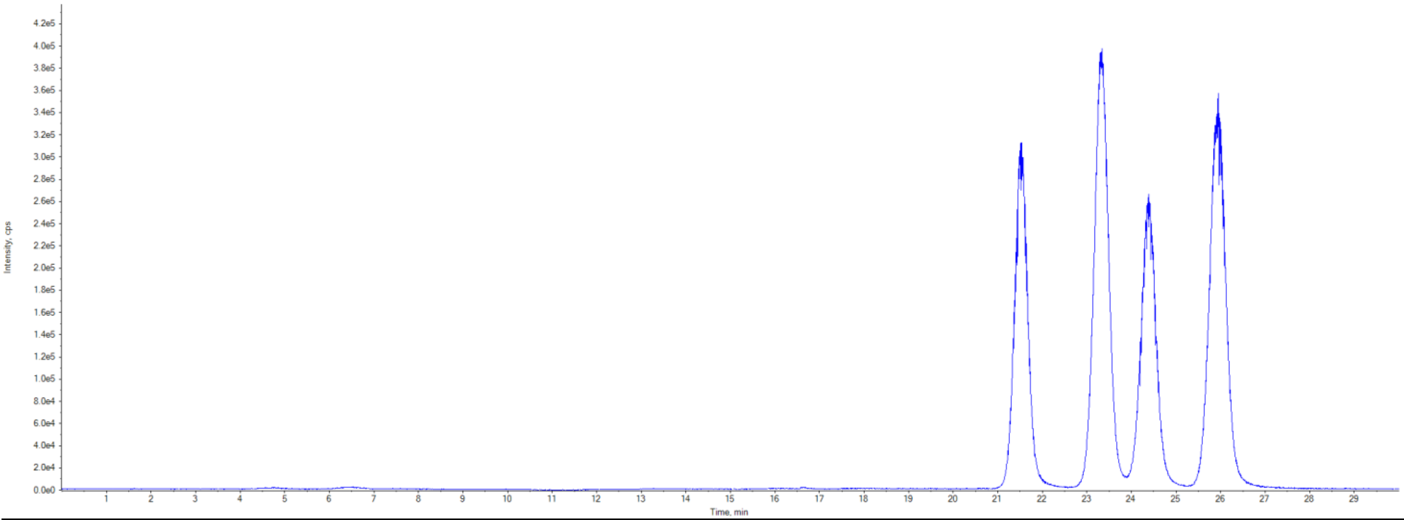


| | | | |
|---|---|---|---|
| **Sample Name** | 0.02-20d-1 | **Injection Vial** | 94 |
| **Data File** | 20220822 BHZ-YP-4du.wiff | **Injection Volume** | 10 |
| **Acquisition Date** | 8/22/2022 10:30:23 AM | **Algorithm Used** | MQL |
| **Acquisition Method** | BHZ  method.dam | **Sample Type** | Unknown |
| **Instrument Name** | AB SCIEX Triple Quad 4500 | **Result Table** | 4-0.02-20d-1.rdb |
| **Sample ID** | No data for Sample ID | **Dilution Factor** | 1.00 |
| **Sample Comment** | No data for Sample Comment | **Weight to Volume** | 0.00 |

Approved By (Date and Initials):______________________________________________.

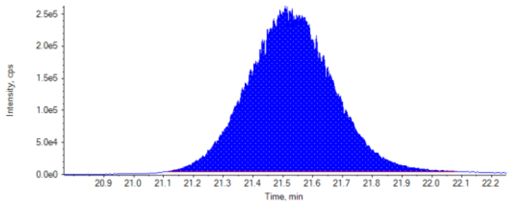


| | |
|---|---|
| **Compound Name:** | F1 **(**342.000/159.100 Da**)** |
| Expected RT: | 21.5 |
| Actual RT: | 21.5 |
| Equation: | At least 2 points are required to calculate regression. |
| Area Counts: | 5.28e+006 |
| ISTD Area Counts: | N/A |
| Amount: | 0.00 (ng/mL) |

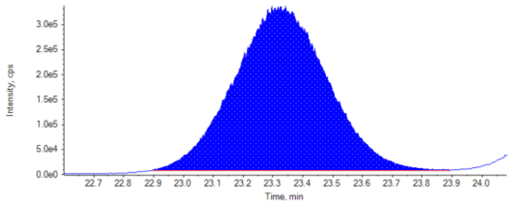


| **Compound Name:** | F2 **(**342.000/159.100 Da**)** |
|---|---|
| Expected RT: | 23.3 |
| Actual RT: | 23.3 |
| Equation: | At least 2 points are required to calculate regression. |
| Area Counts: | 7.32e+006 |
| ISTD Area Counts: | N/A |
| Amount: | 0.00 (ng/mL) |

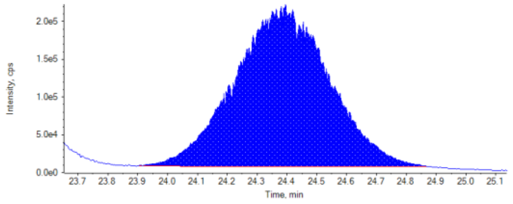


| **Compound Name:** | F3 **(**342.000/159.100 Da**)** |
|---|---|
| Expected RT: | 24.4 |
| Actual RT: | 24.4 |
| Equation: | At least 2 points are required to calculate regression. |
| Area Counts: | 4.75e+006 |
| ISTD Area Counts: | N/A |
| Amount: | 0.00 (ng/mL) |

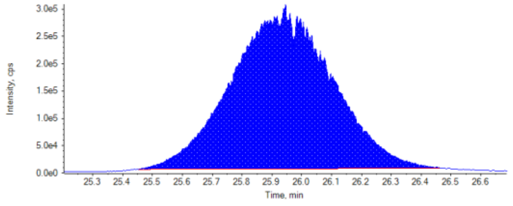


| **Compound Name:** | F4 **(**342.000/159.100 Da**)** |
|---|---|
| Expected RT: | 25.9 |
| Actual RT: | 25.9 |
| Equation: | At least 2 points are required to calculate regression. |
| Area Counts: | 7.30e+006 |
| ISTD Area Counts: | N/A |
| Amount: | 0.00 (ng/mL) |

Figure S28 The LC chromatogram of plums treated with 0.020 a.i. g/L propiconazole at 20 d (4℃)

Figure Caption
Figure_29_SuppInfo.

| | | | |
|---|---|---|---|
| **Data File** | 20220822 BHZ-YP-4du.wiff | **Result Table** | 4-0.02-25d-2.rdb |
| **Acquisition Date** | 8/22/2022 9:27:31 AM | **Algorithm Used** | MQL |
| **Acquisition Method** | BHZ method.dam | **Instrument Name** | AB SCIEX Triple Quad 4500 |
| **Project** | DPY | **Processing Method** | No data for Processing Method |

**Sample Name:** 0.02-25d-2 **Vial #:** 92

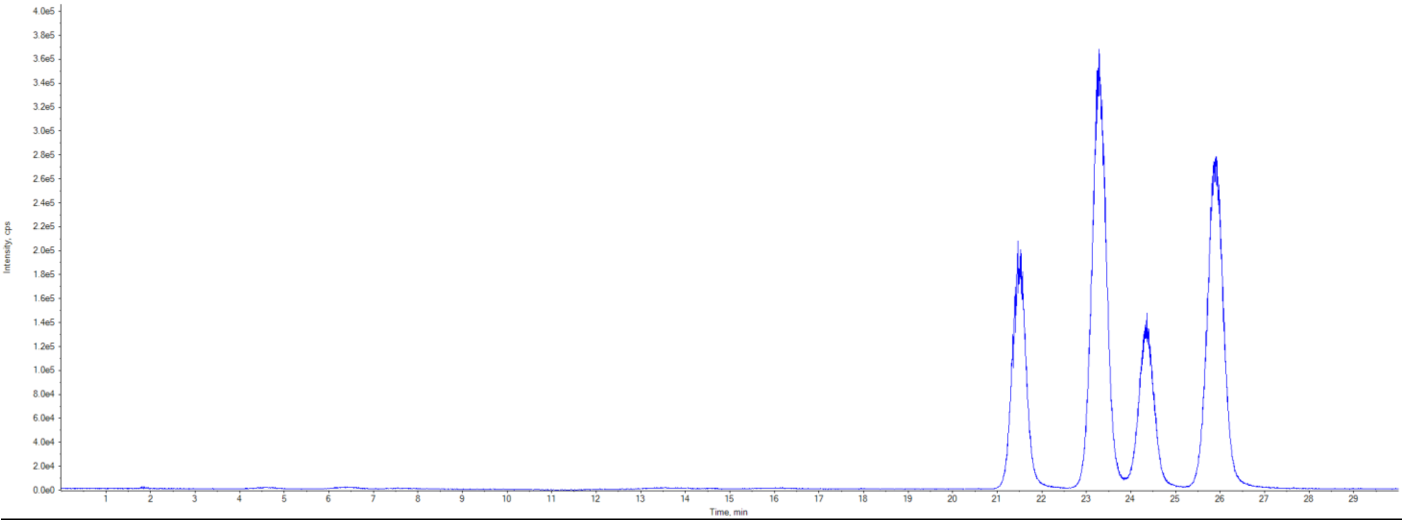


| | | | |
|---|---|---|---|
| **Sample Name** | 0.02-25d-2 | **Injection Vial** | 92 |
| **Data File** | 20220822 BHZ-YP-4du.wiff | **Injection Volume** | 10 |
| **Acquisition Date** | 8/22/2022 9:27:31 AM | **Algorithm Used** | MQL |
| **Acquisition Method** | BHZ method.dam | **Sample Type** | Unknown |
| **Instrument Name** | AB SCIEX Triple Quad 4500 | **Result Table** | 4-0.02-25d-2.rdb |
| **Sample ID** | No data for Sample ID | **Dilution Factor** | 1.00 |
| **Sample Comment** | No data for Sample Comment | **Weight to Volume** | 0.00 |

Approved By (Date and Initials):______________________________________________.

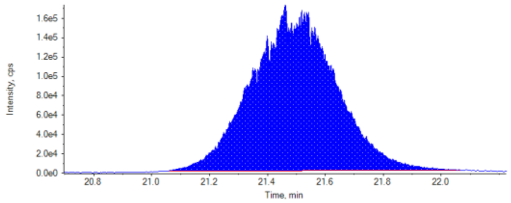


| | |
|---|---|
| **Compound Name:** | F1 (342.000/159.100 Da) |
| Expected RT: | 21.5 |
| Actual RT: | 21.5 |
| Equation: | At least 2 points are required to calculate regression. |
| Area Counts: | 3.44e+006 |
| ISTD Area Counts: | N/A |
| Amount: | 0.00 (ng/mL) |

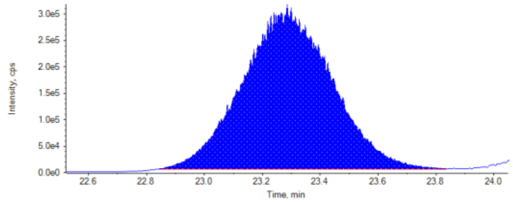


| **Compound Name:** | F2 **(**342.000/159.100 Da**)** |
|---|---|
| Expected RT: | 23.3 |
| Actual RT: | 23.3 |
| Equation: | At least 2 points are required to calculate regression. |
| Area Counts: | 6.55e+006 |
| ISTD Area Counts: | N/A |
| Amount: | 0.00 (ng/mL) |

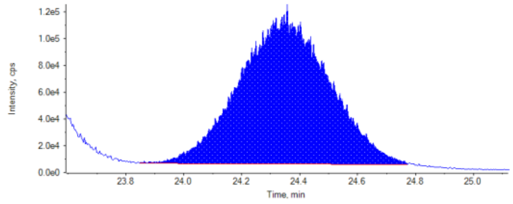


| **Compound Name:** | F3 **(**342.000/159.100 Da**)** |
|---|---|
| Expected RT: | 24.4 |
| Actual RT: | 24.4 |
| Equation: | At least 2 points are required to calculate regression. |
| Area Counts: | 2.44e+006 |
| ISTD Area Counts: | N/A |
| Amount: | 0.00 (ng/mL) |

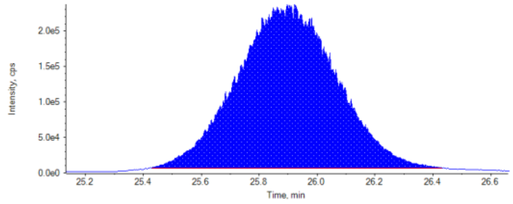


| **Compound Name:** | F4 **(**342.000/159.100 Da**)** |
|---|---|
| Expected RT: | 25.9 |
| Actual RT: | 25.9 |
| Equation: | At least 2 points are required to calculate regression. |
| Area Counts: | 5.89e+006 |
| ISTD Area Counts: | N/A |
| Amount: | 0.00 (ng/mL) |

Figure S29 The LC chromatogram of plums treated with 0.020 a.i. g/L propiconazole at 25 d (4℃)

Created with *No data for MQ Created With*
Printed:*No data for MQ Report Generation Date*

Figure Caption
Figure_30_SuppInfo.

| | | | |
|---|---|---|---|
| **Data File** | 20220822 BHZ-YP-4du.wiff | **Result Table** | 4-0.02-35d-2.rdb |
| **Acquisition Date** | 8/22/2022 6:18:45 AM | **Algorithm Used** | MQL |
| **Acquisition Method** | BHZ method.dam | **Instrument Name** | AB SCIEX Triple Quad 4500 |
| **Project** | DPY | **Processing Method** | *No data for Processing Method* |

**Sample Name:** 0.02-35d-2 **Vial #:** 86

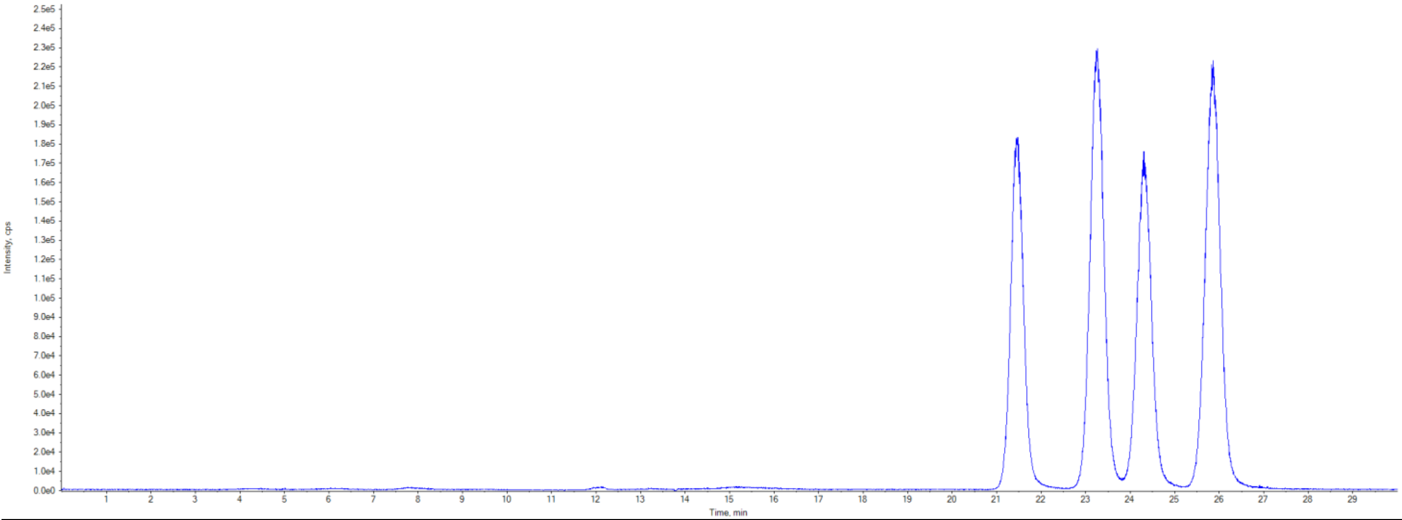

| | | | |
|---|---|---|---|
| **Sample Name** | 0.02-35d-2 | **Injection Vial** | 86 |
| **Data File** | 20220822 BHZ-YP-4du.wiff | **Injection Volume** | 10 |
| **Acquisition Date** | 8/22/2022 6:18:45 AM | **Algorithm Used** | MQL |
| **Acquisition Method** | BHZ method.dam | **Sample Type** | Unknown |
| **Instrument Name** | AB SCIEX Triple Quad 4500 | **Result Table** | 4-0.02-35d-2.rdb |
| **Sample ID** | *No data for Sample ID* | **Dilution Factor** | 1.00 |
| **Sample Comment** | *No data for Sample Comment* | **Weight to Volume** | 0.00 |

Approved By (Date and Initials):________________________________________________.

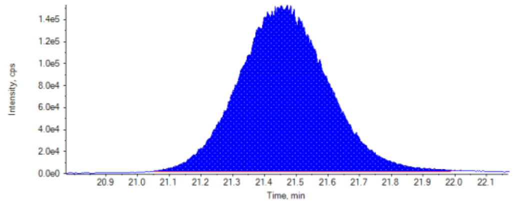

| | |
|---|---|
| **Compound Name:** | F1 **(**342.000/159.100 Da**)** |
| Expected RT: | 21.5 |
| Actual RT: | 21.5 |
| Equation: | At least 2 points are required to calculate regression. |
| Area Counts: | 3.11e+006 |
| ISTD Area Counts: | N/A |
| Amount: | 0.00 (ng/mL) |

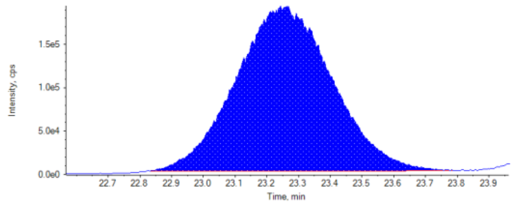


| Compound Name: | F2 (342.000/159.100 Da) |
|---|---|
| Expected RT: | 23.3 |
| Actual RT: | 23.3 |
| Equation: | At least 2 points are required to calculate regression. |
| Area Counts: | 4.11e+006 |
| ISTD Area Counts: | N/A |
| Amount: | 0.00 (ng/mL) |

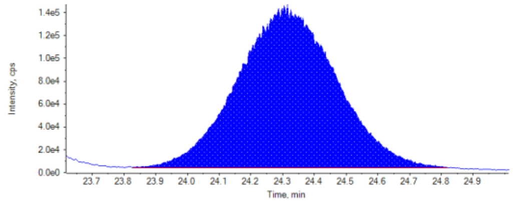


| Compound Name: | F3 (342.000/159.100 Da) |
|---|---|
| Expected RT: | 24.3 |
| Actual RT: | 24.3 |
| Equation: | At least 2 points are required to calculate regression. |
| Area Counts: | 3.06e+006 |
| ISTD Area Counts: | N/A |
| Amount: | 0.00 (ng/mL) |

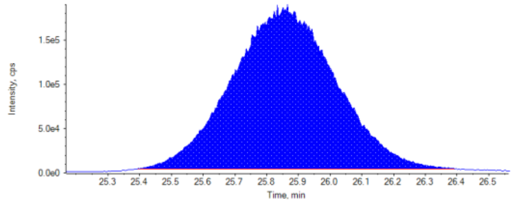


| Compound Name: | F4 (342.000/159.100 Da) |
|---|---|
| Expected RT: | 25.9 |
| Actual RT: | 25.9 |
| Equation: | At least 2 points are required to calculate regression. |
| Area Counts: | 4.23e+006 |
| ISTD Area Counts: | N/A |
| Amount: | 0.00 (ng/mL) |

Figure S30 The LC chromatogram of plums treated with 0.020 a.i. g/L propiconazole at 35 d (4°C)

Figure Caption
Figure_31_SuppInfo.

| | | | |
|---|---|---|---|
| **Data File** | BHZ 20220911.wiff | **Result Table** | 4-0.02-45d-3.rdb |
| **Acquisition Date** | 9/11/2022 5:09:17 PM | **Algorithm Used** | MQL |
| **Acquisition Method** | BHZ method.dam | **Instrument Name** | AB SCIEX Triple Quad 4500 |
| **Project** | DPY | **Processing Method** | No data for Processing Method |

**Sample Name:** 4-0.02-45d-3 **Vial #:** 17

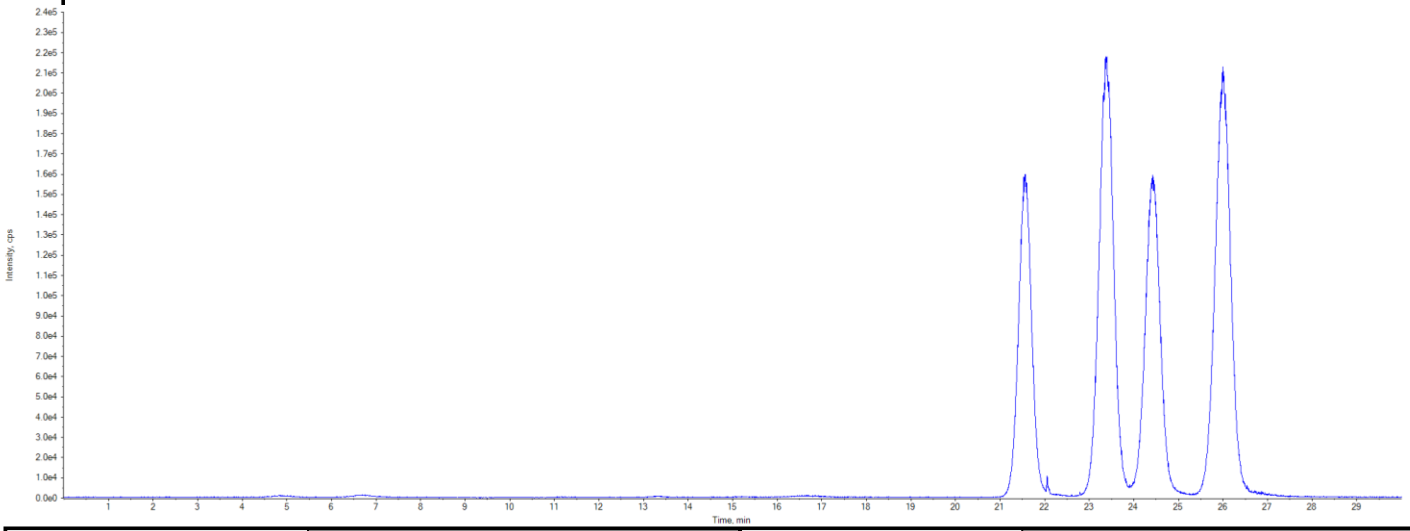


| | | | |
|---|---|---|---|
| **Sample Name** | 4-0.02-45d-3 | **Injection Vial** | 17 |
| **Data File** | BHZ 20220911.wiff | **Injection Volume** | 10 |
| **Acquisition Date** | 9/11/2022 5:09:17 PM | **Algorithm Used** | MQL |
| **Acquisition Method** | BHZ method.dam | **Sample Type** | Unknown |
| **Instrument Name** | AB SCIEX Triple Quad 4500 | **Result Table** | 4-0.02-45d-3.rdb |
| **Sample ID** | No data for Sample ID | **Dilution Factor** | 1.00 |
| **Sample Comment** | No data for Sample Comment | **Weight to Volume** | 0.00 |

Approved By (Date and Initials):________________________________________.

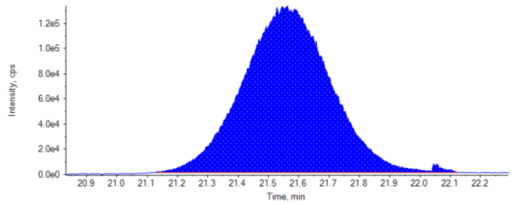


| | |
|---|---|
| **Compound Name:** | F1 **(**342.000/159.100 Da**)** |
| Expected RT: | 21.6 |
| Actual RT: | 21.6 |
| Equation: | At least 2 points are required to calculate regression. |
| Area Counts: | 2.71e+006 |
| ISTD Area Counts: | N/A |
| Amount: | 0.00 (ng/mL) |

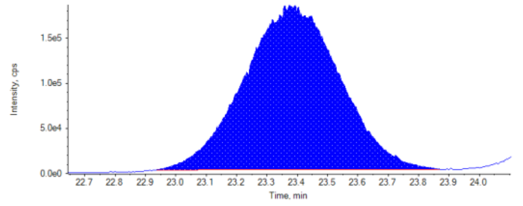


| | |
|---|---|
| **Compound Name:** | F2 **(**342.000/159.100 Da**)** |
| Expected RT: | 23.4 |
| Actual RT: | 23.4 |
| Equation: | At least 2 points are required to calculate regression. |
| Area Counts: | 4.08e+006 |
| ISTD Area Counts: | N/A |
| Amount: | 0.00 (ng/mL) |

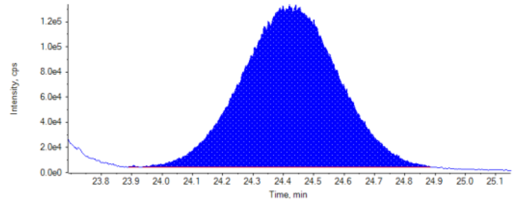


| | |
|---|---|
| **Compound Name:** | F3 **(**342.000/159.100 Da**)** |
| Expected RT: | 24.4 |
| Actual RT: | 24.4 |
| Equation: | At least 2 points are required to calculate regression. |
| Area Counts: | 2.95e+006 |
| ISTD Area Counts: | N/A |
| Amount: | 0.00 (ng/mL) |

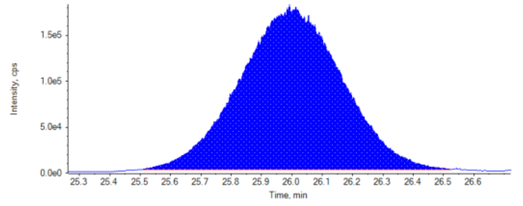


| | |
|---|---|
| **Compound Name:** | F4 **(**342.000/159.100 Da**)** |
| Expected RT: | 26.0 |
| Actual RT: | 26.0 |
| Equation: | At least 2 points are required to calculate regression. |
| Area Counts: | 4.30e+006 |
| ISTD Area Counts: | N/A |
| Amount: | 0.00 (ng/mL) |

Figure S31 The LC chromatogram of plums treated with 0.020 a.i. g/L propiconazole at 45 d (4℃)

Created with *No data for MQ Created With*
Printed:*No data for MQ Report Generation Date*

Figure Caption
Figure_32_SuppInfo.

| | | | |
|---|---|---|---|
| **Data File** | BHZ 20220911.wiff | **Result Table** | 4-0.02-55d-3.rdb |
| **Acquisition Date** | 9/11/2022 3:34:54 PM | **Algorithm Used** | MQL |
| **Acquisition Method** | BHZ method.dam | **Instrument Name** | AB SCIEX Triple Quad 4500 |
| **Project** | DPY | **Processing Method** | *No data for Processing Method* |

**Sample Name:** 4-0.02-55d-3 **Vial #:** 14

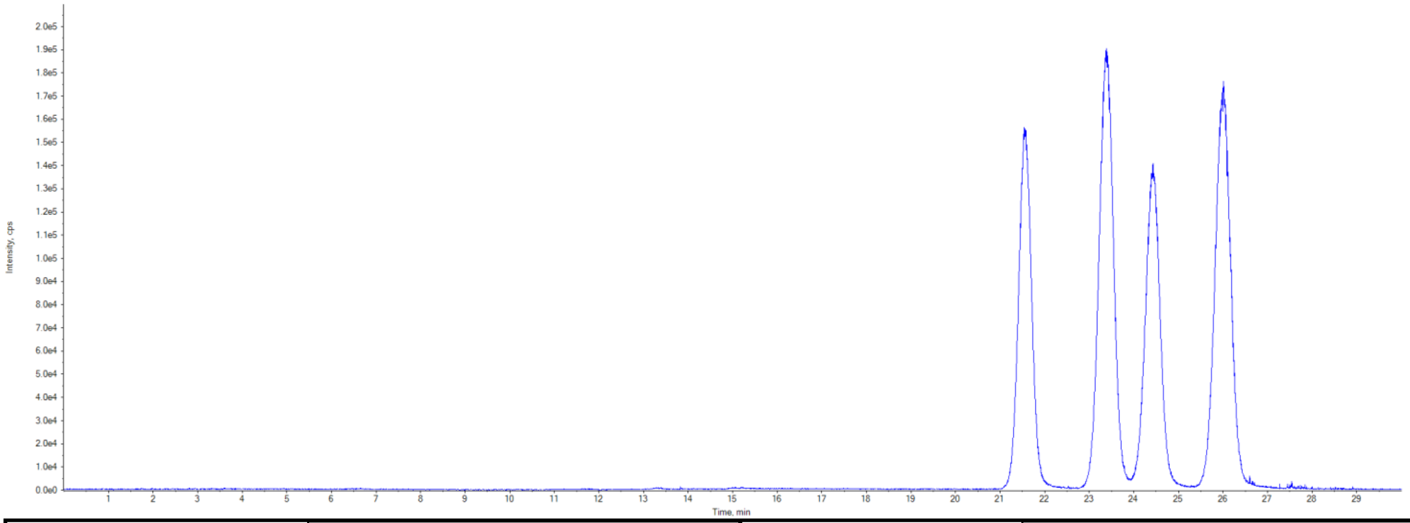


| | | | |
|---|---|---|---|
| **Sample Name** | 4-0.02-55d-3 | **Injection Vial** | 14 |
| **Data File** | BHZ 20220911.wiff | **Injection Volume** | 10 |
| **Acquisition Date** | 9/11/2022 3:34:54 PM | **Algorithm Used** | MQL |
| **Acquisition Method** | BHZ method.dam | **Sample Type** | Unknown |
| **Instrument Name** | AB SCIEX Triple Quad 4500 | **Result Table** | 4-0.02-55d-3.rdb |
| **Sample ID** | *No data for Sample ID* | **Dilution Factor** | 1.00 |
| **Sample Comment** | *No data for Sample Comment* | **Weight to Volume** | 0.00 |

Approved By (Date and Initials):__________________________________________________.

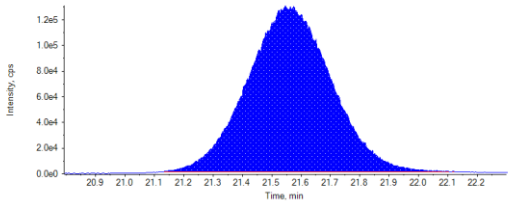


| **Compound Name:** | F1 **(**342.000/159.100 Da**)** |
|---|---|
| Expected RT: | 21.5 |
| Actual RT: | 21.5 |
| Equation: | At least 2 points are required to calculate regression. |
| Area Counts: | 2.64e+006 |
| ISTD Area Counts: | N/A |
| Amount: | 0.00 (ng/mL) |

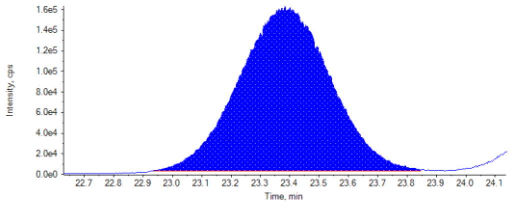


| Compound Name: | F2 (342.000/159.100 Da) |
|---|---|
| Expected RT: | 23.4 |
| Actual RT: | 23.4 |
| Equation: | At least 2 points are required to calculate regression. |
| Area Counts: | 3.54e+006 |
| ISTD Area Counts: | N/A |
| Amount: | 0.00 (ng/mL) |

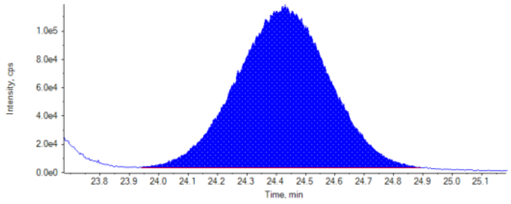


| Compound Name: | F3 (342.000/159.100 Da) |
|---|---|
| Expected RT: | 24.4 |
| Actual RT: | 24.4 |
| Equation: | At least 2 points are required to calculate regression. |
| Area Counts: | 2.56e+006 |
| ISTD Area Counts: | N/A |
| Amount: | 0.00 (ng/mL) |

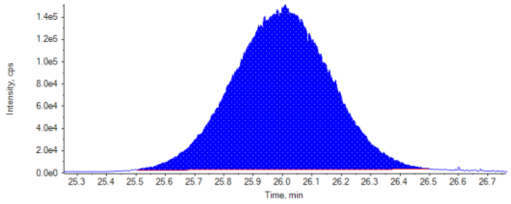


| Compound Name: | F4 (342.000/159.100 Da) |
|---|---|
| Expected RT: | 26.0 |
| Actual RT: | 26.0 |
| Equation: | At least 2 points are required to calculate regression. |
| Area Counts: | 3.53e+006 |
| ISTD Area Counts: | N/A |
| Amount: | 0.00 (ng/mL) |

Figure S32 The LC chromatogram of plums treated with 0.020 a.i. g/L propiconazole at 55 d (4℃)

Created with No data for MQ Created With
Printed:No data for MQ Report Generation Date

Figure Caption
Figure_33_SuppInfo.

| | | | |
|---|---|---|---|
| **Data File** | BHZ 20220911.wiff | **Result Table** | QX-0.004-55d-2.rdb |
| **Acquisition Date** | 9/12/2022 7:50:26 AM | **Algorithm Used** | MQL |
| **Acquisition Method** | BHZ  method.dam | **Instrument Name** | AB SCIEX Triple Quad 4500 |
| **Project** | DPY | **Processing Method** | No data for Processing Method |

**Sample Name:** 4-0.004-55d-QX-2 **Vial #:** 43

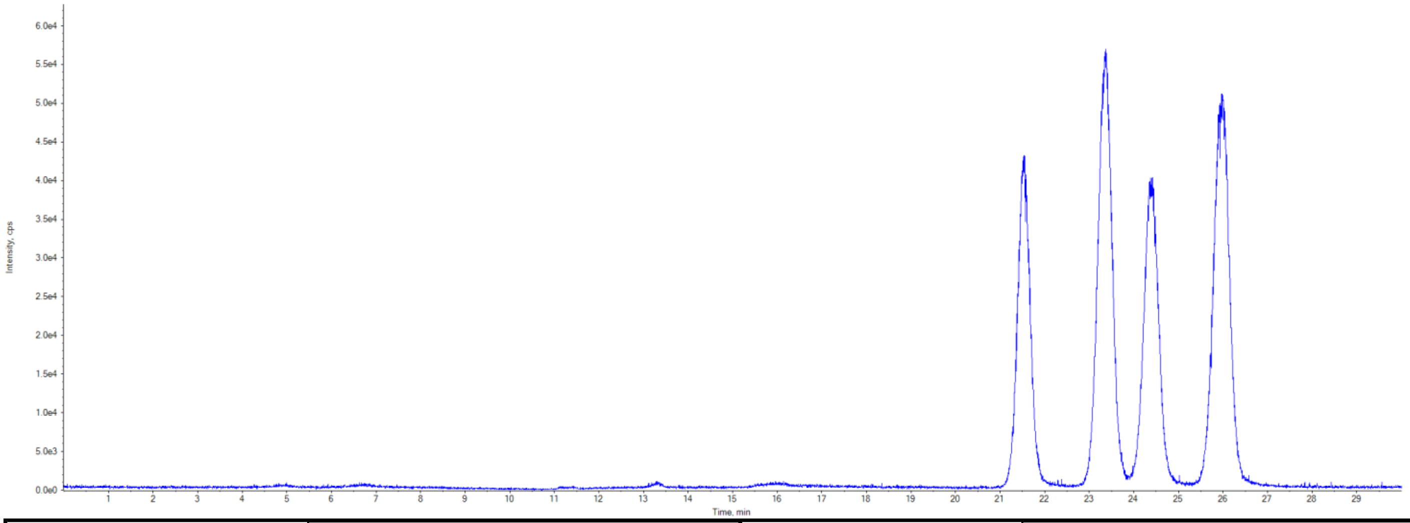


| | | | |
|---|---|---|---|
| **Sample Name** | 4-0.004-55d-QX-2 | **Injection Vial** | 43 |
| **Data File** | BHZ 20220911.wiff | **Injection Volume** | 10 |
| **Acquisition Date** | 9/12/2022 7:50:26 AM | **Algorithm Used** | MQL |
| **Acquisition Method** | BHZ  method.dam | **Sample Type** | Unknown |
| **Instrument Name** | AB SCIEX Triple Quad 4500 | **Result Table** | QX-0.004-55d-2.rdb |
| **Sample ID** | No data for Sample ID | **Dilution Factor** | 1.00 |
| **Sample Comment** | No data for Sample Comment | **Weight to Volume** | 0.00 |

Approved By (Date and Initials):_______________________________________________.

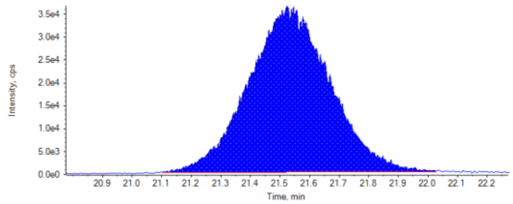


| | |
|---|---|
| **Compound Name:** | F1 **(**342.000/159.100 Da**)** |
| Expected RT: | 21.5 |
| Actual RT: | 21.5 |
| Equation: | At least 2 points are required to calculate regression. |
| Area Counts: | 7.07e+005 |
| ISTD Area Counts: | N/A |
| Amount: | 0.00 (ng/mL) |

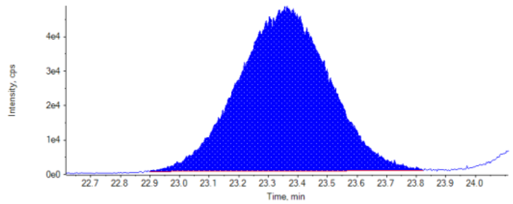


| Compound Name: | F2 (342.000/159.100 Da) |
|---|---|
| Expected RT: | 23.4 |
| Actual RT: | 23.4 |
| Equation: | At least 2 points are required to calculate regression. |
| Area Counts: | 1.04e+006 |
| ISTD Area Counts: | N/A |
| Amount: | 0.00 (ng/mL) |

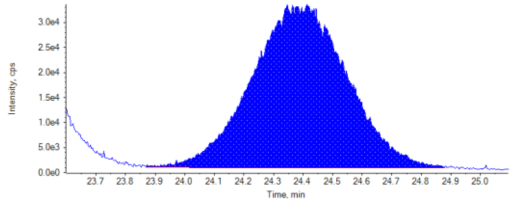


| Compound Name: | F3 (342.000/159.100 Da) |
|---|---|
| Expected RT: | 24.3 |
| Actual RT: | 24.3 |
| Equation: | At least 2 points are required to calculate regression. |
| Area Counts: | 7.41e+005 |
| ISTD Area Counts: | N/A |
| Amount: | 0.00 (ng/mL) |

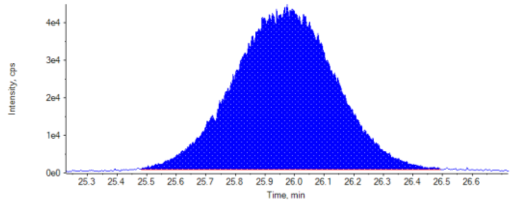


| Compound Name: | F4 (342.000/159.100 Da) |
|---|---|
| Expected RT: | 26.0 |
| Actual RT: | 26.0 |
| Equation: | At least 2 points are required to calculate regression. |
| Area Counts: | 1.05e+006 |
| ISTD Area Counts: | N/A |
| Amount: | 0.00 (ng/mL) |

Figure S33 The LC chromatogram of plums treated with 0.04 a.i. g/L propiconazole were cleaned

Figure Caption
Figure_34_SuppInfo.

| | | | |
|---|---|---|---|
| **Data File** | BHZ 20220911.wiff | **Result Table** | QX-0.02-55d-2.rdb |
| **Acquisition Date** | 9/12/2022 6:16:00 AM | **Algorithm Used** | MQL |
| **Acquisition Method** | BHZ method.dam | **Instrument Name** | AB SCIEX Triple Quad 4500 |
| **Project** | DPY | **Processing Method** | No data for Processing Method |

**Sample Name:** 4-0.02-55d-QX-2 **Vial #:** 40

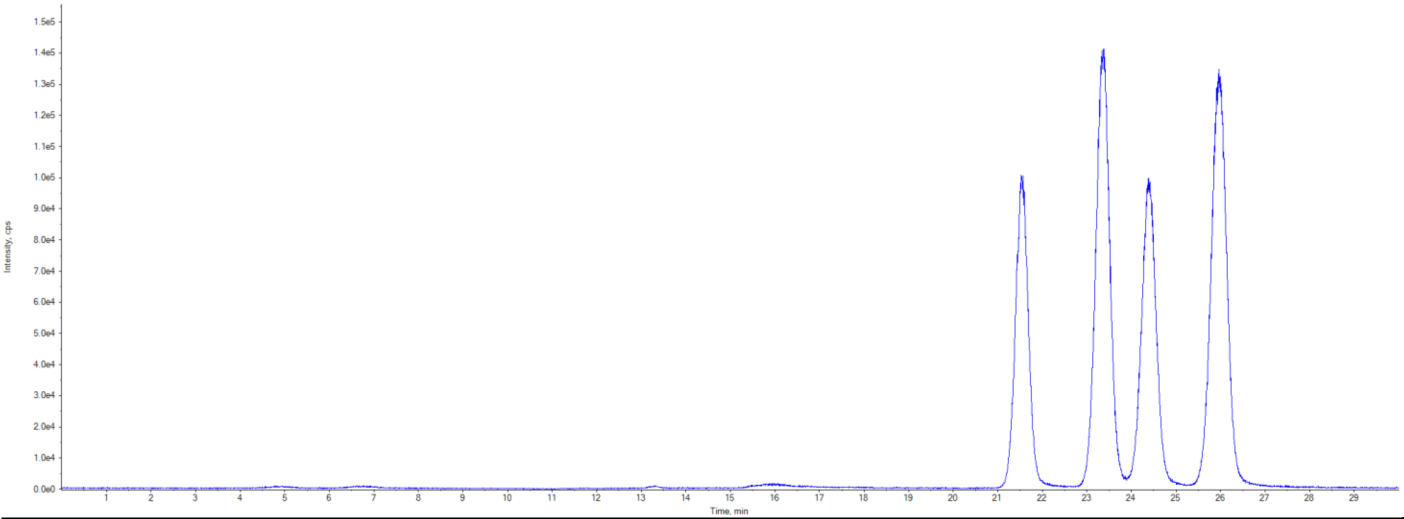


| | | | |
|---|---|---|---|
| **Sample Name** | 4-0.02-55d-QX-2 | **Injection Vial** | 40 |
| **Data File** | BHZ 20220911.wiff | **Injection Volume** | 10 |
| **Acquisition Date** | 9/12/2022 6:16:00 AM | **Algorithm Used** | MQL |
| **Acquisition Method** | BHZ method.dam | **Sample Type** | Unknown |
| **Instrument Name** | AB SCIEX Triple Quad 4500 | **Result Table** | QX-0.02-55d-2.rdb |
| **Sample ID** | No data for Sample ID | **Dilution Factor** | 1.00 |
| **Sample Comment** | No data for Sample Comment | **Weight to Volume** | 0.00 |

Approved By (Date and Initials):______________________________________________.

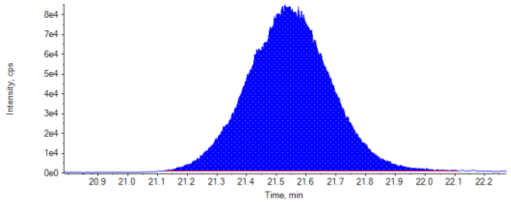


| | |
|---|---|
| **Compound Name:** | F1 **(**342.000/159.100 Da**)** |
| Expected RT: | 21.5 |
| Actual RT: | 21.5 |
| Equation: | At least 2 points are required to calculate regression. |
| Area Counts: | 1.67e+006 |
| ISTD Area Counts: | N/A |
| Amount: | 0.00 (ng/mL) |

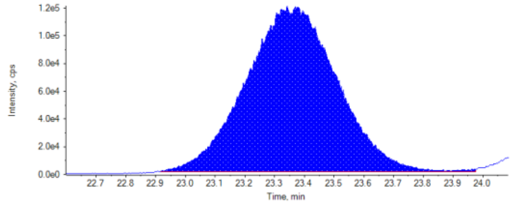


| | |
|---|---|
| **Compound Name:** | F2 **(**342.000/159.100 Da**)** |
| Expected RT: | 23.3 |
| Actual RT: | 23.3 |
| Equation: | At least 2 points are required to calculate regression. |
| Area Counts: | 2.57e+006 |
| ISTD Area Counts: | N/A |
| Amount: | 0.00 (ng/mL) |

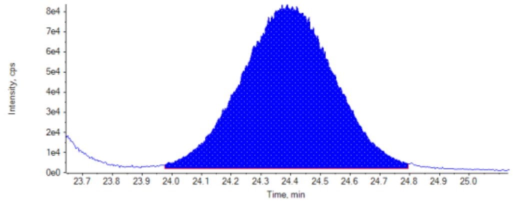


| | |
|---|---|
| **Compound Name:** | F3 **(**342.000/159.100 Da**)** |
| Expected RT: | 24.4 |
| Actual RT: | 24.4 |
| Equation: | At least 2 points are required to calculate regression. |
| Area Counts: | 1.81e+006 |
| ISTD Area Counts: | N/A |
| Amount: | 0.00 (ng/mL) |

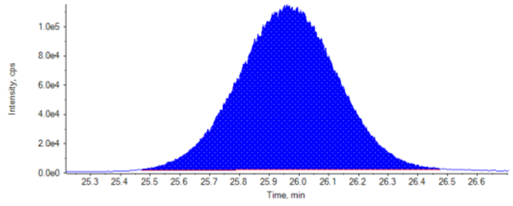


| | |
|---|---|
| **Compound Name:** | F4 **(**342.000/159.100 Da**)** |
| Expected RT: | 26.0 |
| Actual RT: | 26.0 |
| Equation: | At least 2 points are required to calculate regression. |
| Area Counts: | 2.75e+006 |
| ISTD Area Counts: | N/A |
| Amount: | 0.00 (ng/mL) |

Figure S34 The LC chromatogram of plums treated with 0.020 a.i. g/L propiconazole were cleaned
